# Redesigning oxazolidinones as carbonic anhydrase inhibitors against vancomycin-resistant enterococci

**DOI:** 10.1016/j.ejmech.2025.117620

**Published:** 2025-04-11

**Authors:** Andrea Ammara, Simone Giovannuzzi, Alessandro Bonardi, Nader S. Abutaleb, Ahmed A. Abouelkhair, Daniel P. Flaherty, Mohamed N. Seleem, Clemente Capasso, Paola Gratteri, Alessio Nocentini, Claudiu T. Supuran

**Affiliations:** a Neurofarba Department, Pharmaceutical and Nutraceutical Section, University of Florence, Sesto Fiorentino, Italy; b NEUROFARBA Department, Laboratory of Molecular Modeling, Cheminformatics & QSAR, University of Florence, Firenze, Italy; c Department of Biomedical Sciences and Pathobiology, Virginia-Maryland College of Veterinary Medicine, Virginia Polytechnic Institute and State University, Blacksburg, VA, USA; d Center for One Health Research, Virginia Polytechnic Institute and State University, Blacksburg, VA, USA; e Department of Medicinal Chemistry and Molecular Pharmacology, College of Pharmacy, Purdue University, West Lafayette, IN, USA; f Purdue Institute for Drug Discovery, West Lafayette, IN, USA; g Purdue Institute of Inflammation, Immunology and Infectious Disease, West Lafayette, IN, USA; h Istituto di Bioscienze e Biorisorse, CNR, 80131, Naples, Italy

**Keywords:** oxazolidinone, Tedizolid, Carbonic anhydrase inhibition, VRE infections, Bacterial resistance

## Abstract

The rise of vancomycin-resistant enterococci (VRE) as a leading cause of hospital-acquired infections underscores the urgent need for new treatment strategies. In fact, resistance has developed not only to vancomycin but also to other clinically used agents, such as daptomycin and linezolid. We propose a novel drug design approach merging tedizolid, a second-generation oxazolidinone used as an unapproved salvage therapy in clinical settings, with carbonic anhydrase inhibitors (CAIs) recently validated as functioning decolonization agents. These sulfonamide derivatives showed potent inhibition of the carbonic anhydrases from *Enterococcus faecium*, with *K*_I_ values in the range of 14.6–598 nM and 63.2–798 nM against EfCAα and EfCAγ. Computational simulations elucidated the binding mode of these dual-action antibiotics to the peptidyl transferase center (PTC) of the 50S ribosome subunit and bacterial CAs. A subset of six derivatives showed potent PTC-related anti-enterococcal effects against multidrug-resistant *E. faecalis* and *E. faecium* strains with some compounds outperforming both the oxazolidinone and CA inhibitor drugs (MIC values in the range 1–4 μg/mL).

## 1. Introduction

Oxazolidinones are synthetic antibiotics primarily used to treat severe Gram-positive bacterial infections, including those caused by methicillin-resistant *Staphylococcus aureus* (MRSA) and vancomycin-resistant enterococci (VRE) [1–3]. Oxazolidinones work by binding to the peptidyl transferase center in the 50S ribosome subunit, preventing bacterial protein synthesis [4]. Linezolid, the first oxazolidinone, was introduced in 2000 and marked a significant advancement in treatment of Gram-positive bacterial infections [2,5]. Tedizolid, a second-generation oxazolidinone, was approved in 2014 for acute bacterial skin infections and is in Phase III clinical trials for hospital-acquired bacterial pneumonia and ventilated nosocomial pneumonia [6–10]. Importantly, as compared to linezolid, tedizolid showed enhanced potency against MRSA and VRE strains, and improved pharmacokinetics, such as high bioavailability and once-daily dosing. Consequently, this enhanced activity could lead to reducing side effects associated with linezolid like thrombocytopenia [11,12]. *Enterococcus* species, specifically vancomycin-resistant *Enterococcus faecalis* and *Enterococcus faecium*, are the third most commonly isolated Gram-positive pathogens among hospital-acquired infections. Vancomycin-resistant enterococci (VRE) induce a wide array of infections such as endocarditis, urinary tract infections, sepsis, wound infections, skin and skin structure infections, diabetic foot ulcers, and surgical site infections, and pose significant challenges due to the limited treatment options [13,14]. The growing problem of VRE in the healthcare setting is exacerbated by the lack of effective treatments. Currently, linezolid is the only Food and Drug Administration (FDA)-approved therapeutic option for the treatment of VRE [15]. Daptomycin is instead commonly used in this setting in clinical practice, but it lacks an FDA indication for VRE [16]. Linezolid is marginally effective in the treatment of VRE bloodstream infections with a mortality rate of about 30 % and in VRE decolonization [17], and it is also associated with toxic side effects such as myelosuppression, neuropathy, and lactic acidosis [18]. Additionally, resistance has emerged to both linezolid and daptomycin [19,20]. Tedizolid has been clinically used as a salvage therapy though unapproved yet [21]. The rise of VRE as a leading cause of hospital-acquired infections as well as the increased resistance to the current therapeutic options, underscore the urgent need for new treatment strategies [13].

**Fig. 1.**
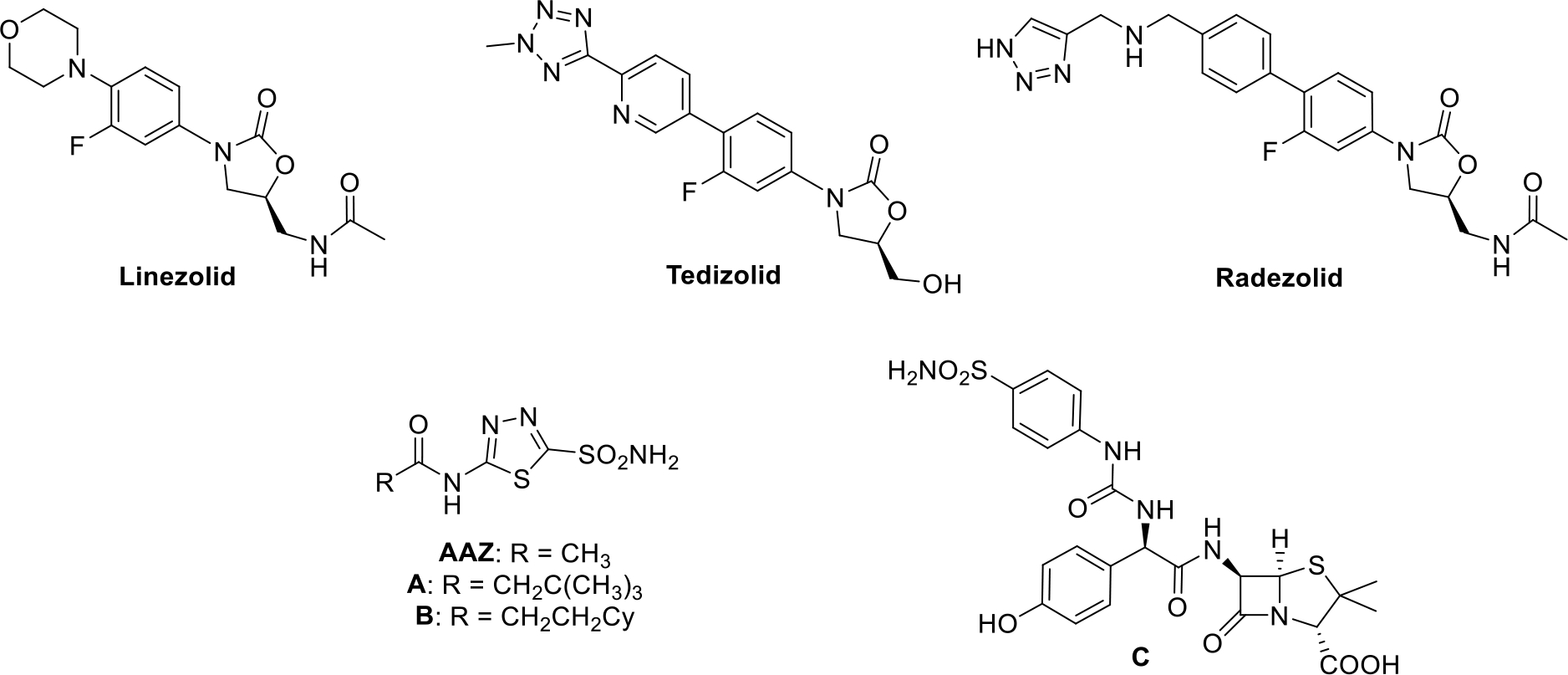
Structures of oxazolidinones and carbonic anhydrase inhibitors discussed here.

Flaherty and colleagues identified α- and γ-class carbonic anhydrases (CAIs) as potential antimicrobial targets to treat VRE infections [22–26]. The CA inhibitor acetazolamide (AAZ, Fig. 1) and a series of its derivatives (*e.g.* **A** and **B** in Fig. 1) showed strong CA inhibition profiles and potent MIC values against *E. faecium* strains, outperforming linezolid in several cases [23]. Moreover, the *in vivo* efficacy of some CA inhibitors such as AAZ, dorzolamide, CAI0019, CAI0028 and CAI0031 was evaluated against VRE in a VRE colonization reduction mouse model and those agents significantly reduced the burden of VRE outperforming the activity of the drug of choice linezolid [24]. This suggests that sulfonamide CAIs show promise to be utilized as effective decolonization agents.

In a scenario of resistance to novel and single-target antibiotics typically developing within a few years of their commercialization [27], combination therapies and multi-targeting drugs emerge as a promising strategy to reduce the trigger of rapid resistance by targeting multiple bacterial pathways [28]. We recently proposed multi-targeting antibiotic compounds incorporating a benzenesulfonamide CA inhibitory scaffold with the β-lactam amoxicillin and ampicillin (*e.g.* compound **C** in Fig. 1) against *N. gonorrhoeae* [29]. Similarly, a series of ciprofloxacin derivatives including CA inhibitory chemotypes was described by some of us as agents for the treatment of *Pseudomonas aeruginosa* [30]. Moreover, the hybrid dual-action strategy (*e.g.* merged with quinolone antibiotics) was also shown to be valuable for discovering novel oxazolidinone candidates [31]. Here, we propose instead the design, synthesis and biological evaluation of novel oxazolidinone-based CA inhibitors against VRE infections.

## 2. Results and discussion

### Design.

**Fig. 2.**
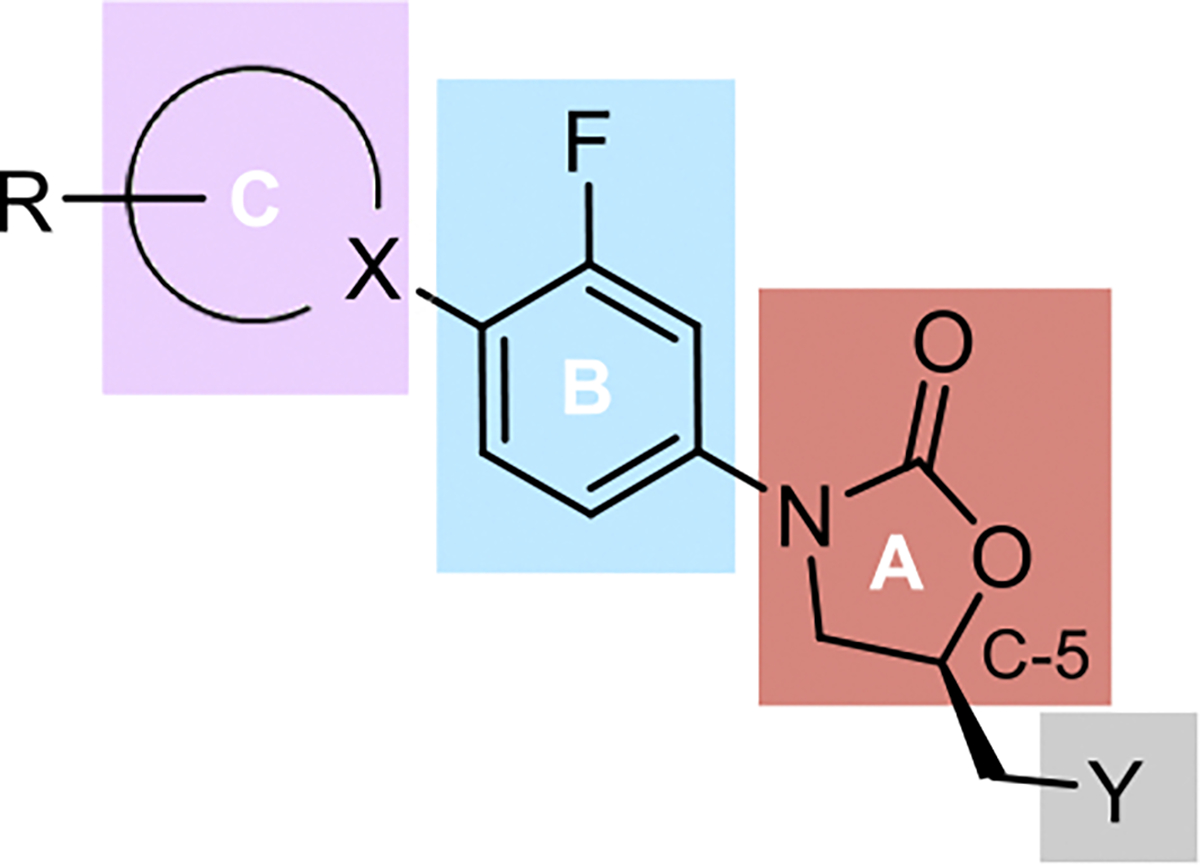
Structure activity relationship of oxazolidinones. **Ring A:** oxazolidinone. The (*S*)-configuration at the C-5 position is essential for antibacterial activity; N-aryl is necessary. **Y group:** optimal potency is achieved with NHCOCH_3_ and small heteroaryl groups; the presence of an OH group reduces susceptibility to resistance mediated by cfr-methyltransferase. **B-Ring substituent:** incorporating fluorine typically enhances activity by 2–8 fold. **X (B–C ring attachment):** an electron-rich nitrogen is well tolerated; aryl or heteroaryl C–C linkages improve potency. **C-ring and R substituents:** there is broad tolerance for structural changes; aryl and heteroaryl rings improve potency and reduce the risk of resistance due to ribosomal mutations.

Oxazolidinones show relatively flexible structure-activity relationships with even minor modifications to their core resulting in significant alterations in biological activity (Fig. 2) [3,31–33]. Briefly, the pharmacophoric core includes the oxazolidinone ring (ring A) with the *S* configuration of the substituent at C-5 and an N-arylsubstituent (ring B), with a *m*-fluoro substitution increasing the biological activity, and a *p*-substitution (ring C), including the R tail, to additionally expand the antibacterial spectrum. The Y group can be an acetamide portion (*e.g.* linezolid and congeners and radezolid), a OH (*e.g.* tedizolid) or small heteroaryl groups (*e.g.* contezolid). An electron-rich nitrogen is well tolerated as X group in the B–C ring attachment, while aryl or heteroaryl C–C linkages improve potency. Aryl (*e.g.* radezolid) and heteroaryl (*e.g.* tedizolid) scaffolds as C-ring improve potency and reduce the risk of resistance due to ribosomal mutations. There is broad tolerance for structural changes at the R group.

**Fig. 3.**
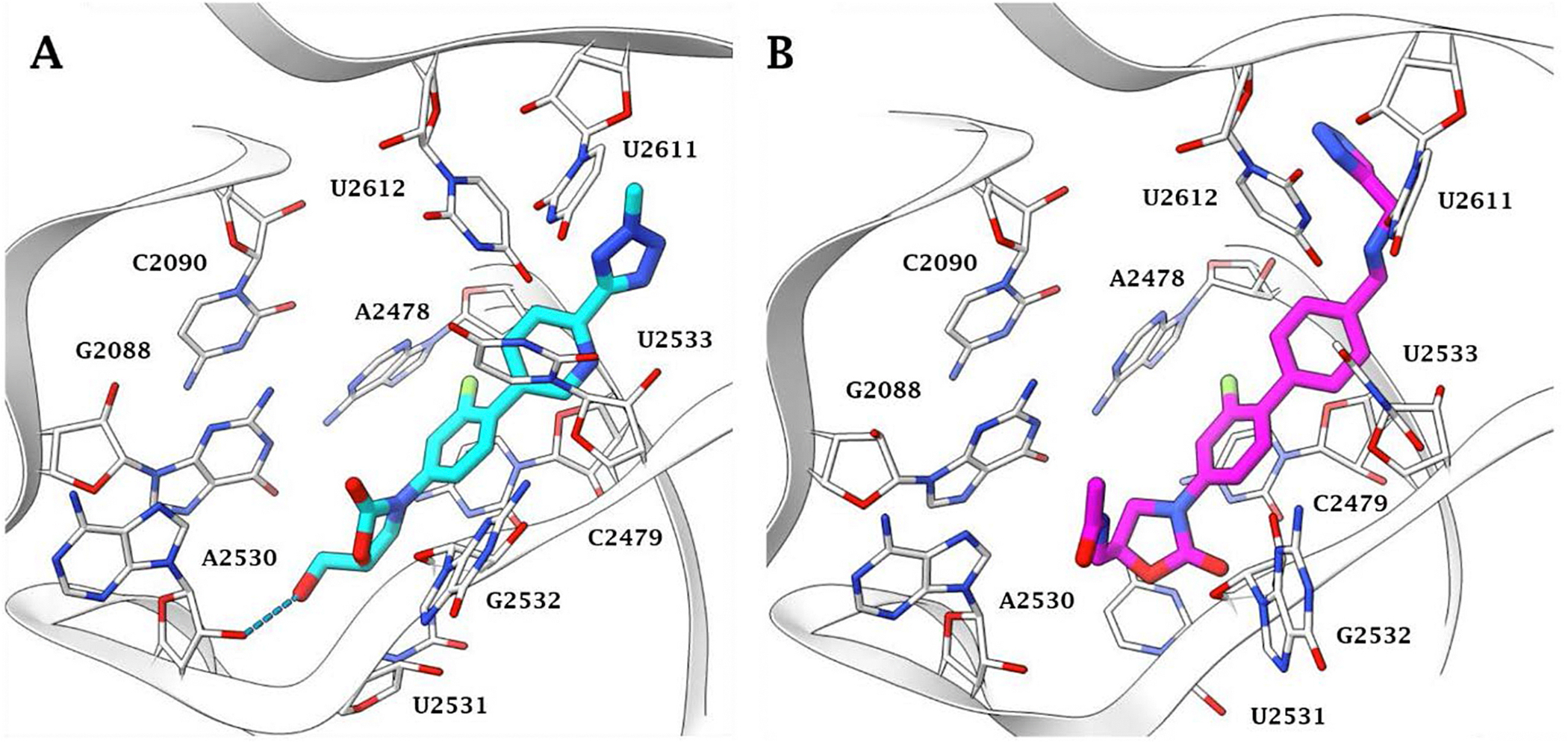
Cryo-EM of A) tedizolid and B) radezolid bound in the PTC of the 50S subunit of the MRSA ribosome (PDB 6WRS and 6WQQ, respectively).

Several oxazolidinones have been co-crystalized with 50S ribosome subunit from different species such as linezolid to *Haloarcula marismortui* [34]*, Escherichia coli* [35], and *S. aureus* [36]. Lately cryo-electron microscopy shed light on the binding mode of ligands such as tedizolid and radezolid to the peptidyl transferase center (PTC) of the 50S ribosomal subunit from methicillin-resistant *S. aureus* (MRSA) [37]. This structural information clearly showed how the presence of the acetamido or heteroaryl groups in the Y oxazolidinone portion (such as linezolid or radezolid in Fig. 3B) is detrimental to the antibacterial activity in presence of the *cfr* gene [38]. The latter encodes a methyltransferase acting on adenine at position 2530 (A2530, Fig. 3) of the 23S rRNA, altering the ribosomal binding site and interaction of the acetamido group with the ribosome. The presence of an OH group (as in tedizolid, Fig. 3A) reduces susceptibility to resistance mediated by cfr-methyltransferase.

**Fig. 4.**
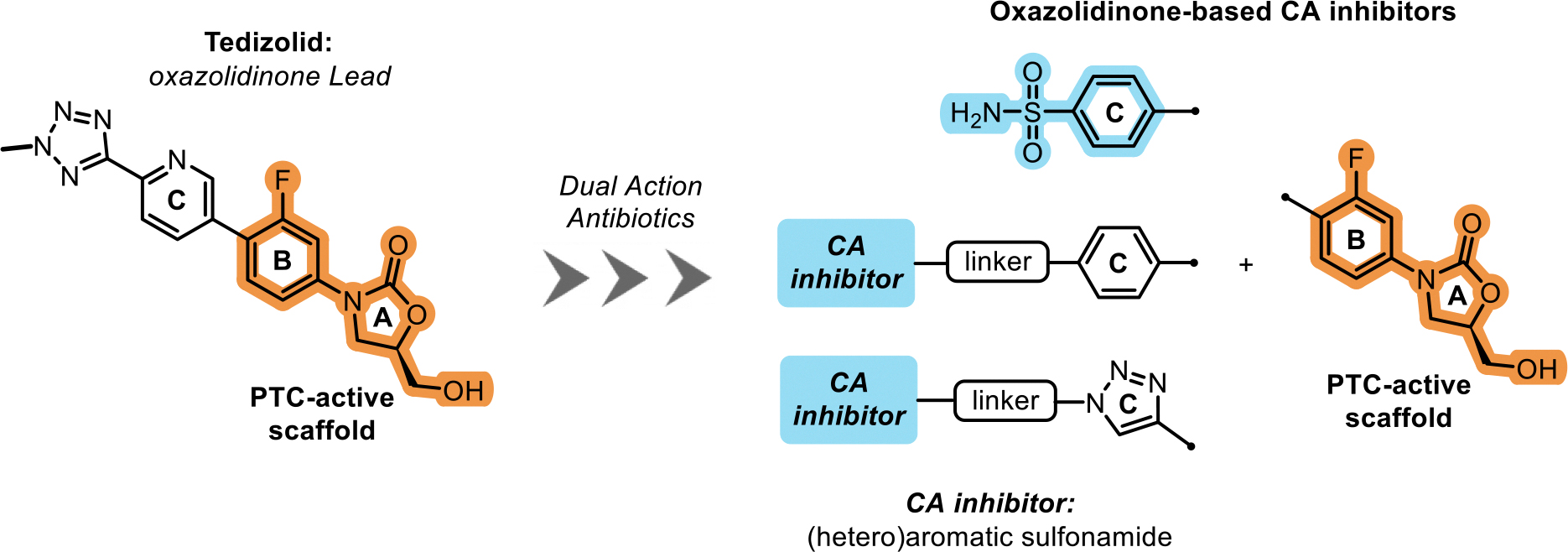
Drug design of oxazolidinone-based CA inhibitors.

Considering the aforementioned information and SAR of oxazolidinones, we selected the tedizolid framework to design hybrid derivatives that incorporate (hetero)aromatic sulfonamides with CA inhibitory properties (Fig. 4). The CA inhibitory portion was incorporated in the oxazolidinone scaffold by replacing the pyridine C ring of tedizolid with: (i) a benzenesulfonamide; (ii) a triazole to append a (hetero)aromatic sulfonamide via an aliphatic or acetamide linker; (iii) a phenyl ring to append a (hetero)aromatic sulfonamide via different type and length linkers.

#### Chemistry.

**Scheme 1.**
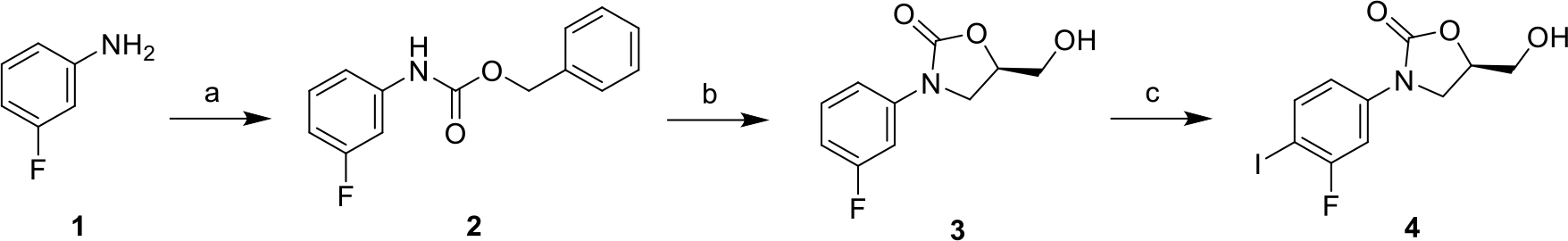
Synthetic pathway to yield the oxazolidinone intermediate **4**. Reagents and reaction conditions: a) phenyl chloroformate, K_2_CO_3_, acetone, 0 °C to rt, 3h; b) (*R*)-glycidyl butyrate, LiHMDS, anhydrous THF, −78 °C to rt, overnight (on); c) NIS, TFA, 0 °C, 3 h.

The *p*-iodinated *N*-(3-fluorophenyl)oxazolidinone derivative **4**, bearing a hydroxymethyl group at the C-5 position as in tedizolid, served as the key intermediate to promote the inclusion of the CA inhibitory portion. It was synthesized *ex-novo* following literature methods (Scheme 1) [39]. Briefly, 3-fluoroaniline **1** was reacted with phenyl chloroformate and K_2_CO_3_ to prepare the carbamate **2** for the subsequent cyclization. The latter was conducted with (*R*)-glycidyl butyrate in presence of LiHMDS as a base, at −78 °C in anhydrous THF to form the oxazolidinone **3**. The selective aromatic iodination was performed with NIS in TFA to give intermediate **4**.

**Scheme 2.**
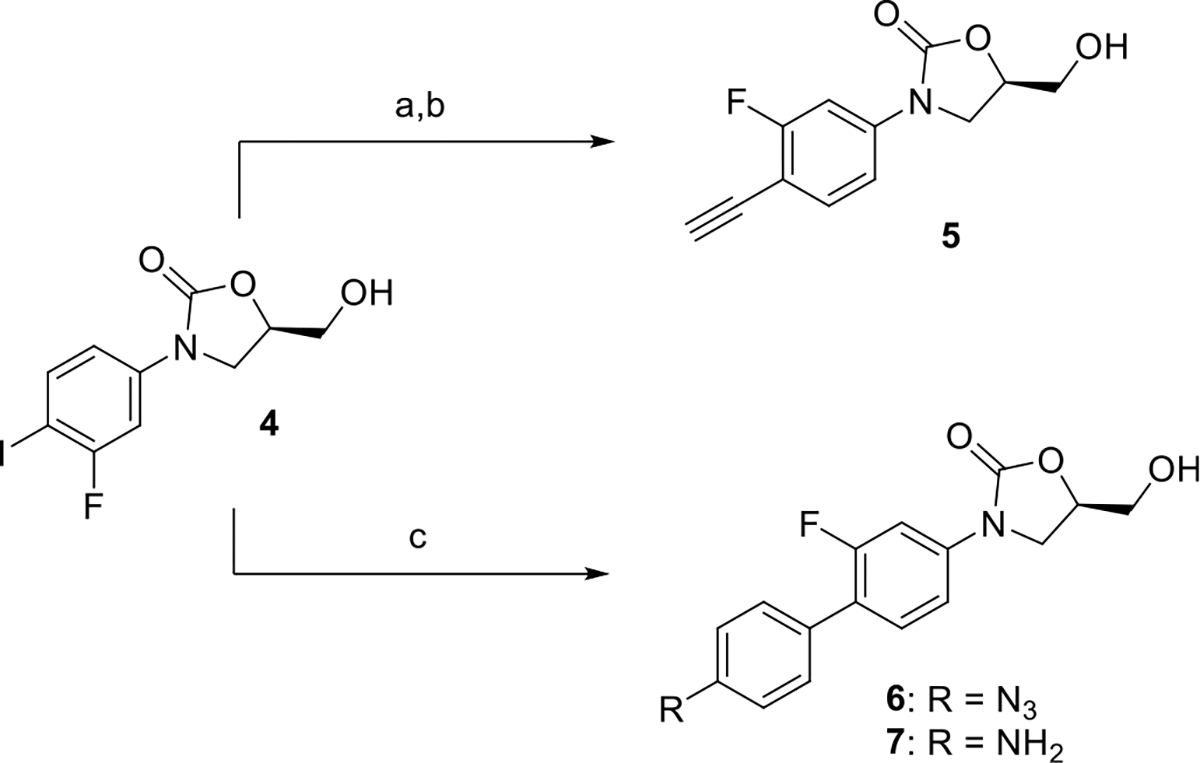
Synthesis of intermediates **4**–**7**. Reagents and reaction conditions: a) TMSA, Et_3_N, Pd(PPh_3_)_4_, CuI, anhydrous DMF, 50 °C, 48 h; b) TBAF, THF, 0 °C to rt, 1 h; c) 4-azidophenylboronic acid pinacol ester or 4-aminophenylboronic acid pinacol ester, K_2_CO_3_, Pd(dppf)Cl_2_, dioxane/EtOH/H_2_O, 90 °C, 4 h.

Thus, the latter was converted to oxazolidinone derivatives **5**–**7** by a Sonogashira or Suzuki reaction for the successive coupling to the CA inhibitor fragments. In detail, alkyne **5** was achieved by treating **4** with trimethylsilylacetylene (TMSA) and Et_3_N in presence of Pd(PPh_3_)_4_ and CuI as catalysts in anhydrous DMF. Compound **4** was reacted with 4-aminophenylboronic acid pinacol ester and 4-azidophenylboronic acid pinacol ester in presence of K_2_CO_3_ and Pd(dppf)Cl_2_ to give intermediates **6** and **7**, respectively (Scheme 2).

**Scheme 3.**
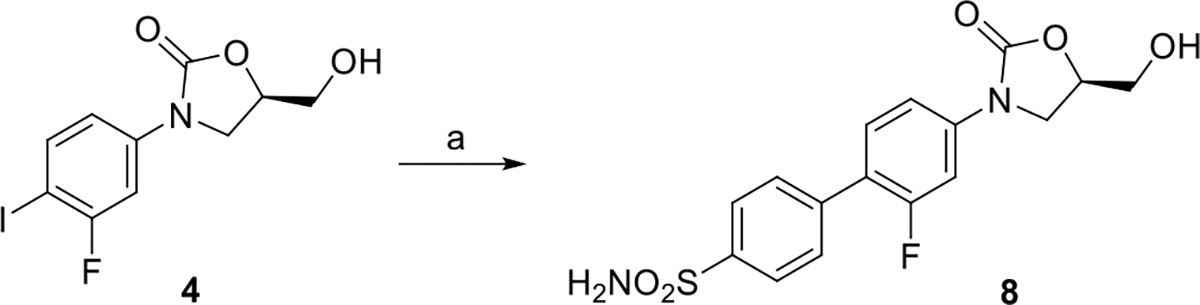
Synthesis of compound **8**. Reagents and reaction conditions: a) **A**, K_2_CO_3_, Pd(dppf)Cl_2_, dioxane/EtOH/H_2_O, 90 °C, 4 h.

**Scheme 4.**
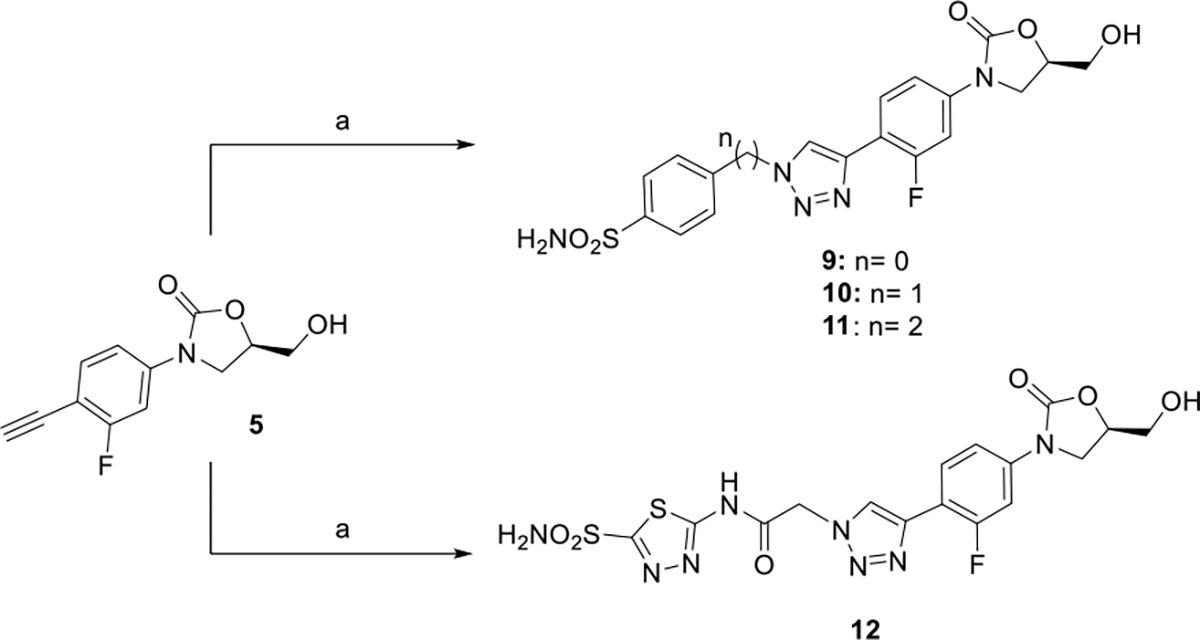
Synthesis of compounds **9–12**. Reagents and reaction conditions: a) **B-E**, Cu(Ac)_2_, sodium ascorbate, MeOH/THF, 40 °C, on.

**Scheme 5.**
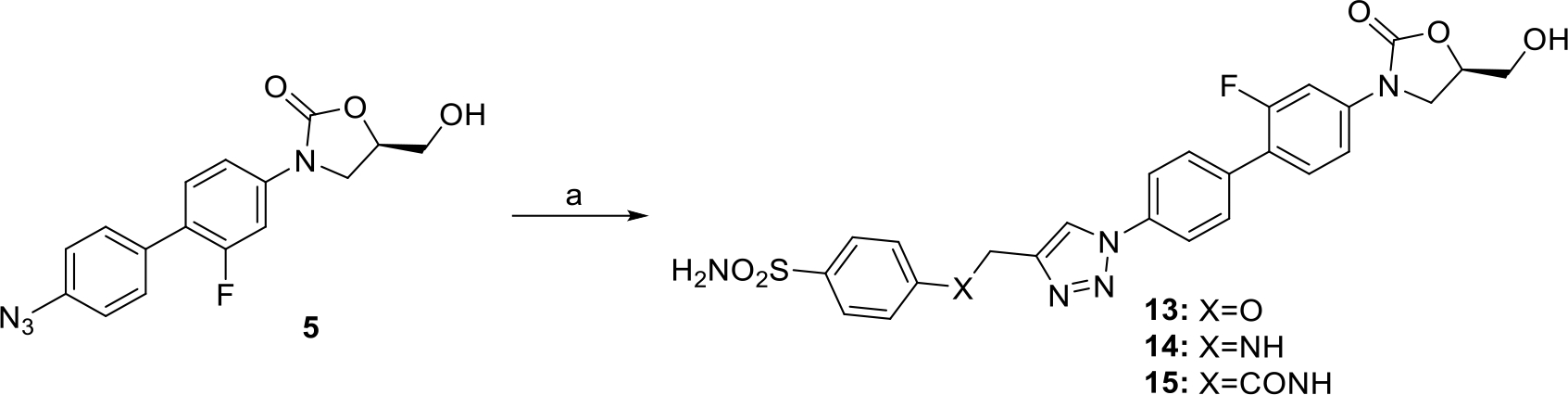
Synthesis of compounds **13**–**15**. Reagents and reaction conditions: a) **F–H**, Cu(Ac)_2_, sodium ascorbate, MeOH/THF, 40 °C, on.

**Scheme 6.**
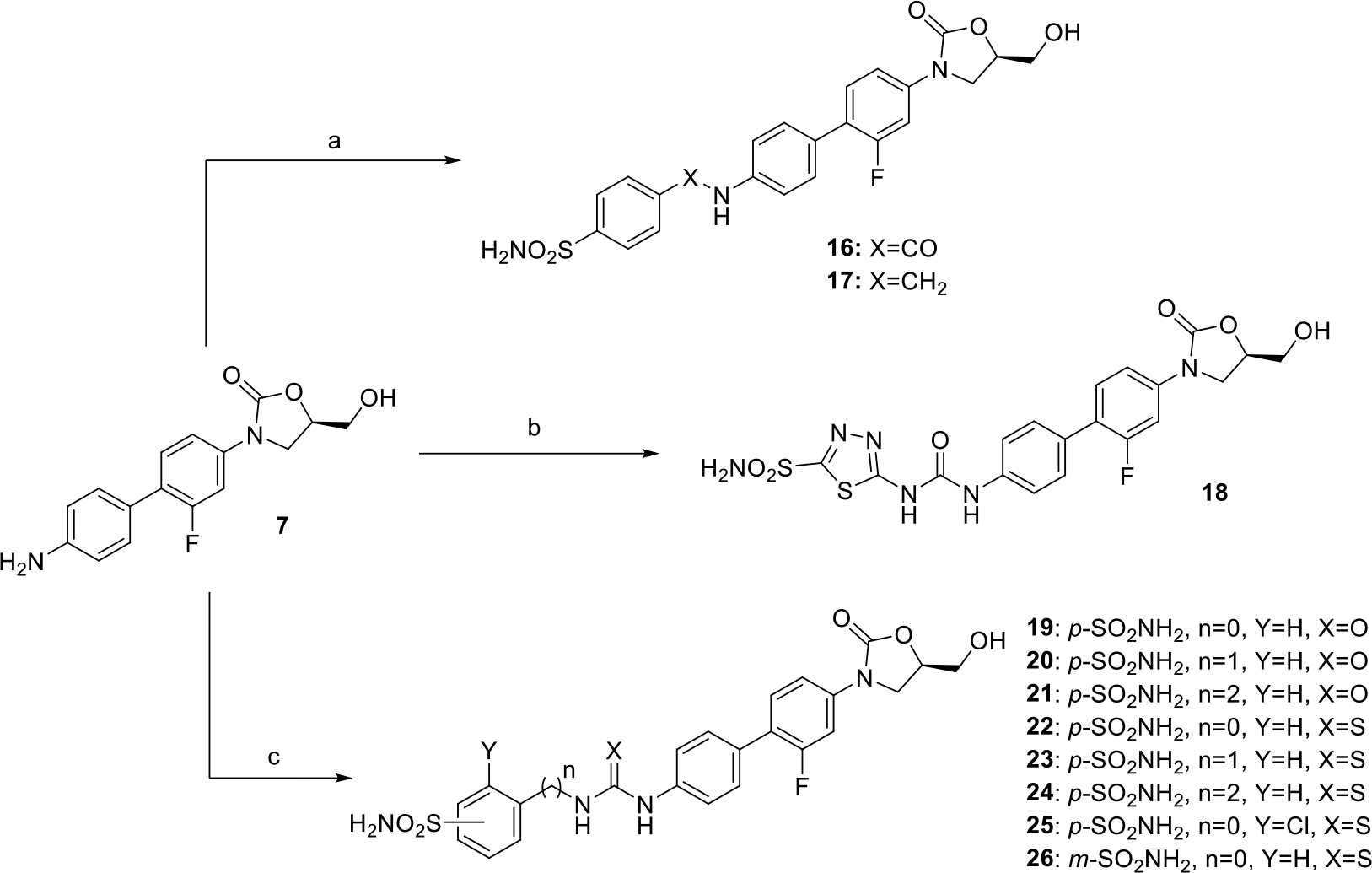
Synthesis of compounds **16**–**26**. Reagents and reaction conditions: a) **I**, pyridine, anhydrous DMF, rt, on; b) **J**, K_2_CO_3_, anhydrous DMF, 60 °C, on; c) **K–R**, Et_3_N, anhydrous ACN, rt or reflux, on.

Oxazolidinone derivatives **4**–**7** were thus reacted with a variety of reagents bearing a (hetero)aromatic sulfonamide (**A-N**, Supporting Information) to obtain the hybrid compounds **8**–**26** (Schemes 3–6). The oxazolidinone benzenesulfonamide derivative **8** was achieved by treating the iodo compound **4** with the 4-sulfamoylphenylboronic acid pinacol ester **A** by a Suzuki reaction as described above (Scheme 3).

A subset of derivatives bearing a triazole linker substituting the tedizolid pyridine ring (**9**–**12**) was achieved by treating the oxazolidinone alkyne **6** with azide intermediates **B-E** by a Huisgen cycloaddition in presence of sodium ascorbate and Cu(Ac)_2_ as catalysts in MeOH/THF at 40 °C (Scheme 4). Derivatives **9**–**11** exhibit a benzenesulfonamide appended to the N1 atom of the triazole linker by no, a methylene or ethylene spacer. Compound **12** shows instead a thiadiazolesulfonamide attached to C-ring by an acetamido function.

Another subset of benzenesulfonamide triazole derivatives incorporating the heterocycle to the phenyl C-ring series (**9**–**11**) was obtained by treating intermediate **7** with alkyne derivatives **F–H** (O, NH or CONH propargyl benzenesulfonamide derivatives) in Huisgen cycloaddition conditions as described above.

Oxazolidinone intermediate **7** was reacted with reagents **I–R** to furnish the sulfonamide hybrid derivatives **16**–**26**. Specifically, the amide **16** was achieved by treating **7** with the freshly prepared acyl chloride **I** using pyridine as a base in anhydrous DMF at room temperature. The 4-sulfamoylbenzyl bromide **J** was reacted with derivative **7** by a nucleophilic substitution in anhydrous DMF with K_2_CO_3_ at 60 °C to yield **17**. Finally, a subset of ureido and thioureido derivatives (**18**–**26**) were obtained by treating **7** with sulfamoyl phenyl carbamates **J-M** or isothiocyanates **N–R** in the presence of K_2_CO_3_ or Et_3_N in anhydrous DMF or ACN. In the end, compounds **8**–**26** were all purified by a silica gel chromatography column using a proper mixture of solvents.

Carbonic anhydrase inhibition assay. The CA inhibition profiles of compounds **8**–**26** were then evaluated against two physiologically relevant isoforms, hCA I, II, and significant bacterial CA isoforms belonging to *E. faecium* (EfCAα and EfCAγ), by a Stopped-Flow CO_2_ hydration assay using **AAZ** as the reference inhibitor (Table 1) [40].

The following structure-activity relationship (SAR) can be gathered from the inhibition data reported in Table 1.

- hCA I is a main off-target isozyme in this study, displaying a widespread expression in human tissues. Interestingly, it is the least inhibited isoform by derivatives **8**–**26**, with inhibition constants (*K*_I_s) ranging from high nanomolar to low micromolar values (298 nM–4190 nM). Among these, compound **8**, that lacks the linker between the CA inhibitor moiety and the oxazolidinone portion, demonstrated one of the most effective inhibitory activities against, with a *K*_I_ of 750 nM. In contrast, the addition of a linker or spacer between the portions generally reduced activity. This trend is evident with derivatives **13**–**26**, whose *K*_I_s ranged from 814 nM to 4190 nM. Notably, ureidic-based derivatives (**18**–**21**) exhibited weaker inhibition, with *K*_I_s between 1810 nM and 4190 nM. Similarly, their thioureidic analogs, **22**, **23**, and **26**, were also less effective, with *K*_I_s of 3050 nM, 2010 nM, and 3620 nM, respectively. However, increased potency was observed in thioureidic compounds **24** and **25**, which displayed *K*_I_s in the high nanomolar range (979 nM and 814 nM, respectively), comparably to compound **8.** Additionally, compounds **9**–**12**, that feature a triazole group instead of a phenyl moiety, showed the most effective inhibition with compound **11** emerging as the most potent hCA I inhibitor (*K*_I_ of 298 nM). It can be assumed that while phenyl and triazole rings are both involved in π-π interactions, only the second one works as a hydrogen bond acceptor, increasing the interactions with polar residues in the active site. Compounds **9**, **10** and **12** were less effective, with *K*_I_s in the range 845–1900 nM. Among these, the type of spacer between the triazole and CAI chemotype played a crucial role in modulating the inhibitory efficacy, as demonstrated by the varying effectiveness of compounds **9**–**11.**
- hCA II, another off-target isozyme, is generally well-inhibited by most compounds tested. The *K*_I_s against hCA II are within the medium-to-high nanomolar range, spanning from 19.4 nM to 262 nM. Compound **8** exhibited the weakest inhibition against hCA II, with a *K*_I_ of 262 nM. A similar pattern was observed for the thioureidic compounds **24** and **25**, that showed *K*_I_s in the high nanomolar range, *i.e.* 121 nM and 103 nM, respectively. It can be noted that compound **25** features a chlorine substituent on the benzene bearing the ZBG which can enhance steric hindrance and affect the interaction with Zn(II). In contrast, the other thioureidic analogs **22** and **23** emerged as potent inhibitors of hCA II, with *K*_I_s in the medium nanomolar range (19.4 nM and 36.1 nM, respectively). As observed with hCA I, the length of the spacer between the two pharmacophores significantly influenced potency. For instance, compound **22**, that lacks a spacer, was the most potent inhibitor against hCA II, with a *K*_I_ of 19.4 nM. This potency decreased 6-fold in compound **24**, that incorporates an ethyl spacer, resulting in a *K*_I_ of 121 nM. Indeed, the spacer can play a crucial role in positioning the thiourea linker optimally to facilitate hydrogen bonds or other interactions with nearby residues within the cavity. The ureidic analogue of compound **22**, namely compound **19**, was 2-fold less potent, with a *K*_I_ of 32.6 nM, though remaining the most effective ureidic inhibitor within subset **18**–**21.** The thioureudic groups is assumed to better fill the cavity and form lipophilic interactions compared to the ureidic moiety. Interestingly, switching the CAI aromatic core did not significantly alter activity, as evidenced by compound **18** (*K*_I_ of 40.6 nM) and compound **19** (*K*_I_ of 32.6 nM). Furthermore, the replacement of the (thio)ureido linker with a triazole ring generally reduced the effectiveness of compounds **13**–**15**, which exhibited *K*_I_s of 175 nM, 89.7 nM, and 139 nM, respectively. Likely, this substitution results in a loss of polar interactions mediated by the thiourea group, which can act as both a hydrogen bond acceptor and donor, in contrast to the triazole, which can only participate in hydrogen bonds as an acceptor. Additionally, the absence of π-π interactions between the triazole ring and nearby residues may further contribute to this effect. However, substituting the phenyl ring C with a triazole generally led to a good inhibitory activity, as with compounds **9**–**12**, that displayed *K*_I_s in the medium-to-high nanomolar range, from 54.9 nM to 117 nM.
- EfCAα is effectively inhibited by all derivatives (**8**–**26**), with *K*_I_ values ranging from medium to high nanomolar levels (14.6–409 nM). Compound **8** demonstrated particularly effective inhibition against this isoform, with a *K*_I_ of 17.5 nM, approximately 14- and 40-fold more potent compared to hCA II and hCA I, respectively. The most potent inhibitor was compound **17**, that features a methylamine linker between fragments and a *K*_I_ of 14.6 nM, that is similar to compound **8.** Converting the amine in compound **17** into an amide reduced its potency by 20-fold, as with compound **16**, that showed a *K*_I_ of 329 nM. While both functional groups can participate in hydrogen bonding, the amide’s planar geometry contrasts with the amine’s tetrahedral configuration, potentially altering spatial alignment with the target binding site. In fact, a similar trend was observed with the switch from amine to (thio)ureido or triazole linker. Specifically, compounds **18**–**26** displayed *K*_I_s in the medium-to-high nanomolar range (58.3–409 nM). Among these, compound **21**, with an ethylureidic linker, was the most effective, showing a *K*_I_ of 58.3 nM. In contrast, congeners **19** and **20**, having different spacer lengths, were less effective inhibitors with *K*_I_s of 169 nM and 94.8 nM, respectively. It can be assumed that compound **21** better positions the oxazolidinone tail within the active site, maximizing interactions with nearby residues more effectively than its congeners, due to its longer spacer. The substitution of the urea with a thiourea also influenced potency, as observed with compound **24** (*K*_I_ of 87.2 nM). Among the thioureidic derivatives, **22**–**24**, the presence or absence of a spacer affected effectiveness, with compound **22** (no spacer, *K*_I_ of 94.8 nM) and compound **24** (ethyl spacer, *K*_I_ of 87.2 nM) showing increased potency. The positioning of the sulfonamide group on the aromatic core, moving from the *para* to *meta* position, did not significantly impact inhibition potency, as observed in compound **26** (*K*_I_ of 71.0 nM). However, adding a halogen atom to the phenyl ring in compound **25** decreased its potency (*K*_I_ of 148 nM). Again, the inclusion of a chlorine atom in the benzene bearing the ZBG can increase the steric hindrance and affect the binding to the Zn(II) ion. Finally, the presence of a triazole ring as a linker generally reduced the inhibitory efficacy of the compounds, as with compounds **9**–**12** and **14**, which exhibited *K*_I_s in the range 136–598 nM. However, effective activity was observed for triazole derivatives **13** and **15**, with *K*_I_s of 91.6 nM and 76.2 nM, respectively.
- EfCAγ, another targeted isozyme in this study, belongs to a distinct CA class compared to those discussed above. Generally, EfCAγ exhibits good to medium susceptibility to inhibition by all derivatives **8**–**26**, with *K*_I_ values ranging from 63.2 nM to 798 nM. Compound **8** was the most potent inhibitor, with a *K*_I_ of 63.2 nM, 5-fold more effective than the reference drug **AAZ** (*K*_I_ of 323 nM). Other derivatives, such as **13**, **17**, **19**, **21**, and **25**, also showed promising inhibition with *K*_I_ values in the medium nanomolar range, spanning between 80.1 nM and 99.1 nM. Among the triazole-based derivatives, compounds **13** and **15** were the most effective, with *K*_I_s of 81.1 nM and 112 nM, respectively, being 2-fold more potent than compound **14**, (*K*_I_ of 216 nM). The substitution of the phenyl ring in C with a triazole in compounds **9**–**12** significantly reduced their inhibition potency, as evidenced by *K*_I_ values in the medium-high nanomolar range (199–686 nM). In contrast, the(thio)ureidic derivatives generally exhibited greater effectiveness. Compounds **18**–**21**, **24**, and **25** showed *K*_I_s ranging from 91.3 nM to 141 nM. Among these, compounds **19** and **21** were the most potent ureidic inhibitors, with *K*_I_s of 91.3 nM and 99.1 nM, respectively. The thioureidic analogue of compound **21**, namely **24**, maintained comparable potency with a *K*_I_ of 112 nM. Unlike EfCAα, compound **25**, that bears a halogen atom in the CAI scaffold, exhibited significant inhibitory activity with a *K*_I_ of 92.4 nM, approximately 4-fold more effective than that of the reference drug **AAZ.** The observed activity difference of compound **25** between EfCAα and EfCAγ likely stems from structural variations in their active sites, particularly in the residues surrounding the Zn(II) ion. These differences make the chlorine substitution uniquely advantageous in EfCAγ′s active site. Compounds **23** and **26** were instead among the weakest inhibitors of this isoform, with *K*_I_ values of 500 nM and 541 nM, respectively. Finally, converting a (thio)ureidic linker into an amide generally reduced inhibition potency, as with compound **16**, that led to the least effective inhibition (*K*_I_ of 798 nM). Conversely, replacing an amide group with an amine significantly enhanced the inhibitor effectiveness, as observed with compound **17** (*K*_I_ of 86.9 nM).

#### Antibacterial activity.

Next, the antibacterial activity of derivatives **8**–**26** was evaluated against a panel of oxazolidinone-sensitive VRE strains (*E. faecalis* NR 31971 and NR 31972, *E. faecium* HM-965). This assessment aimed to elucidate the potential of these compounds as antibacterial agents against VRE, thus proving to point out the contribution and/or involvement of the carbonic anhydrase inhibition into the total antibacterial activity.

Except for several compounds, such as **9**–**12**, **16** and **23** showing no antibacterial activity, all the rest exhibited a potent and promising activity against the tested VRE strains with MICs between 0.125 μg/mL and 8 μg/mL (Table 2). Particularly, derivatives **8** and **13** were detected as the most intriguing inhibitors, exhibiting MIC values within the submicromolar range against all three strains (0.125–0.25 μg/mL). These were followed by derivatives **17**, **20** and **21** which inhibited the tested strains at concentrations ranging from 0.5 to 1 μg/mL. Interestingly, those compounds outperformed **AAZ** and **LNZ**.

Indeed, based on the MICs detected against three oxazolidinone-sensitive VRE strains (MIC linezolid 1 μg/mL), we initiated a comprehensive evaluation focusing on the most potent and promising derivatives. This evaluation was specifically targeted towards assessing their efficacy against an oxazolidinone-resistant VRE strain (*E. faecalis* NR 31903, MIC of linezolid = 16 μg/mL). Compounds **9**–**12**, **16** and **23**, ineffective inhibitors against the oxazolidinone-sensitive strains, were excluded.

Derivatives **13**, **14**, **15**, **18**–**20** and **22** showed no activity against the tested strain (MIC >64 μg/mL), while compounds **8**, **17**, **21**, and **24**–**26** exhibited a very potent activity, inhibiting such resistant strain at concentrations ranging from 1 to 4 μg/mL. This activity was comparable to or notably even exceeds that of **TDZ** (MIC = 4 μg/mL) used as a positive control. The results show that these dual compounds exhibit enhanced antibacterial activity compared to that of the single-target drugs **AAZ**, **LNZ**, and **TDZ**.

#### Cytoxocity toward human cells.

**Fig. 5.**
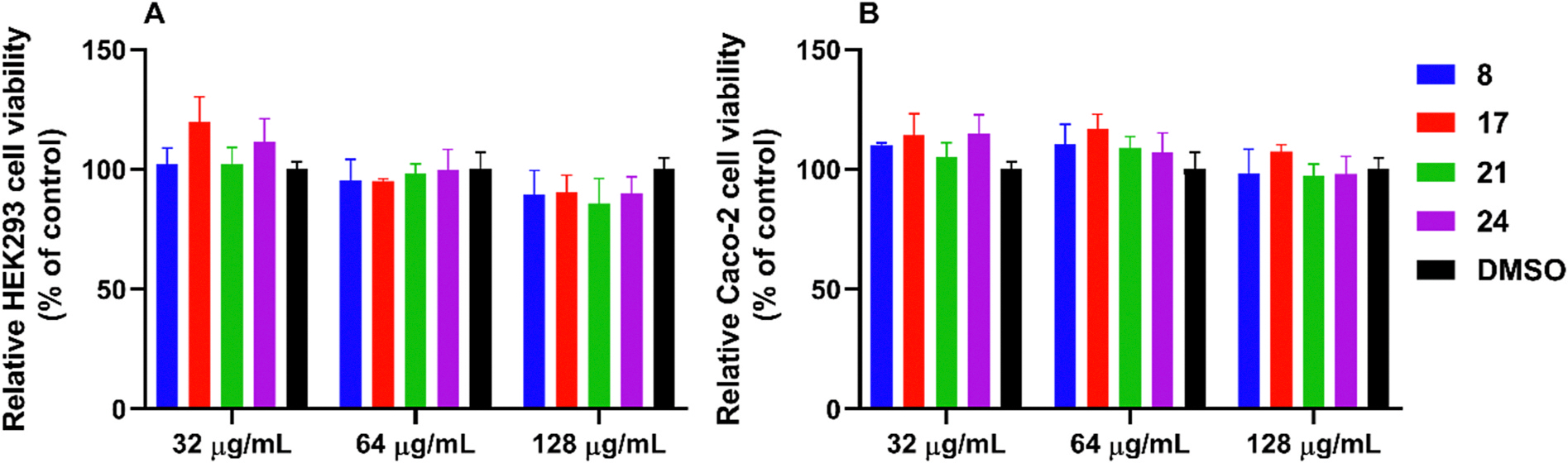
Cell viability for oxazolidinone-sulfonamide hybrids **8**, **17**, **21** and **24** tested at 32, 64, and 128 μg/mL against HEK293 cells (**A**) and Caco-2 cells (**B**) measured via the MTS assay. The results are compared to DMSO (negative control). Absorbance values are calculated as an average of three replicates. Error bars represent standard deviation values. A two-way ANOVA with post hoc Dunnett’s test for multiple comparisons determined non-significant differences for compounds-treated cells as compared to DMSO.

The most effective antibacterial compounds (**8**, **17**, **21** and **24**) were evaluated for cytotoxicity using human epithelial kidney (HEK293) and colorectal adenocarcinoma (Caco-2) cell lines to assess their safety profile in renal and intestinal models. All four compounds exhibited no significant cytotoxicity at concentrations up to 128 μg/mL, maintaining cell viability near 100 % relative to the vehicle control (0.1 % DMSO) (Fig. 5). This high tolerability suggests favorable biocompatibility for further therapeutic exploration.

#### Computational study.

**Fig. 6.**
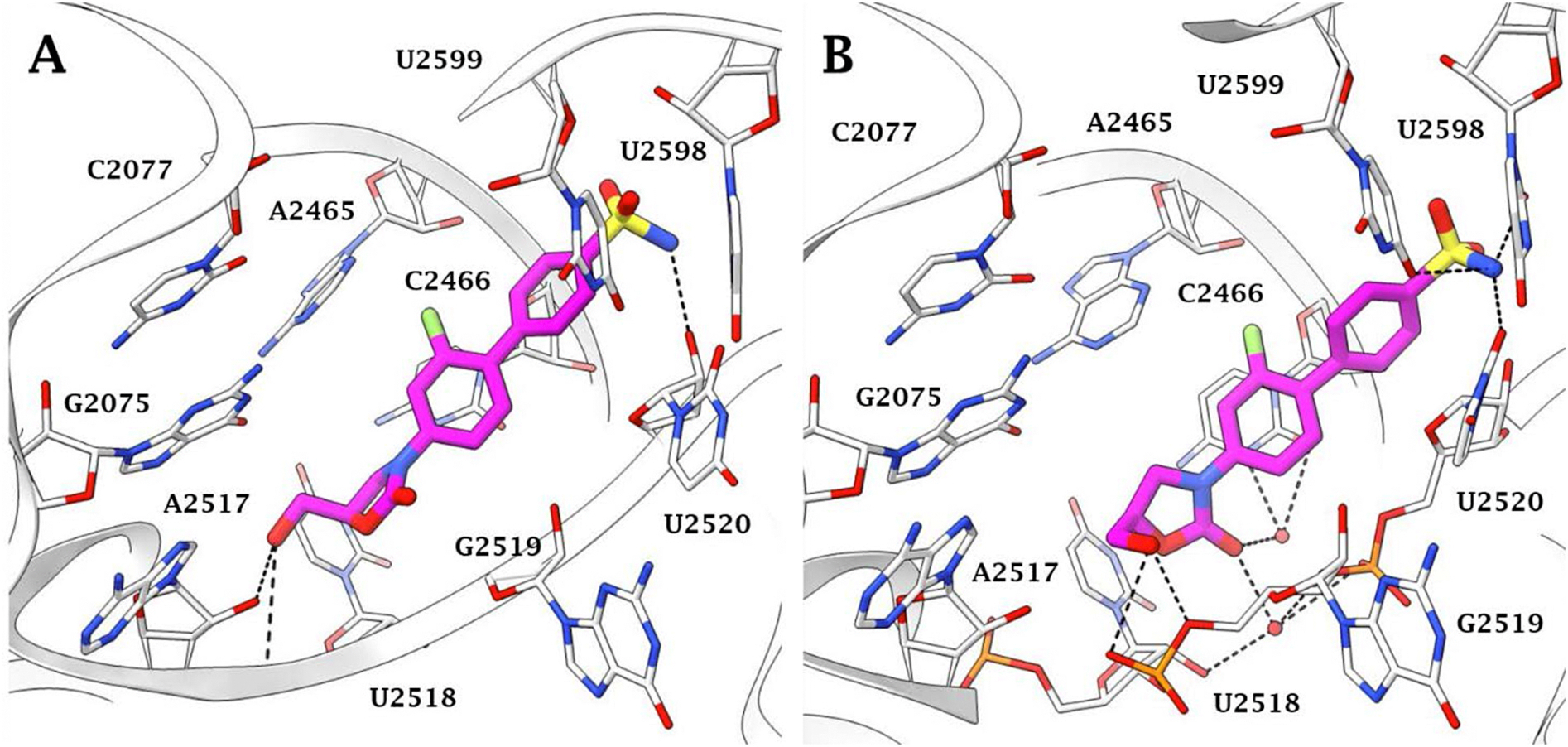
A. Binding mode of **8** in the PTC of the 50S ribosome subunit of *E. faecalis* predicted by docking/MM-GBSA. B. Active site view of the most represented ligand orientation along the 200 ns MD. Water molecules are shown as red spheres. H-bonds are depicted as black dashed lines.

Docking followed by a MM-GBSA based refinement was conducted to assess the binding mode of the compounds most active in the MIC assay (**8**, **17**, **21**) in the PTC of the 50S ribosome subunit of *E. faecalis* to demonstrate their maintained capability to bind in the oxazolidinone binding pocket (Fig. 6) [41]. The structure of the *E. faecalis* 50S subunit was solved by cryo-EM, but not in adduct with an oxazolidinone ligand. However, the high sequence and structural identity shared by the *E. faecalis* 50S subunit with those of *E. coli* and *S. aureus* allowed to identify the phylogenetically conserved nucleotides encompassing the oxazolidinone binding site within the PTC region [42].

**Fig. 7.**
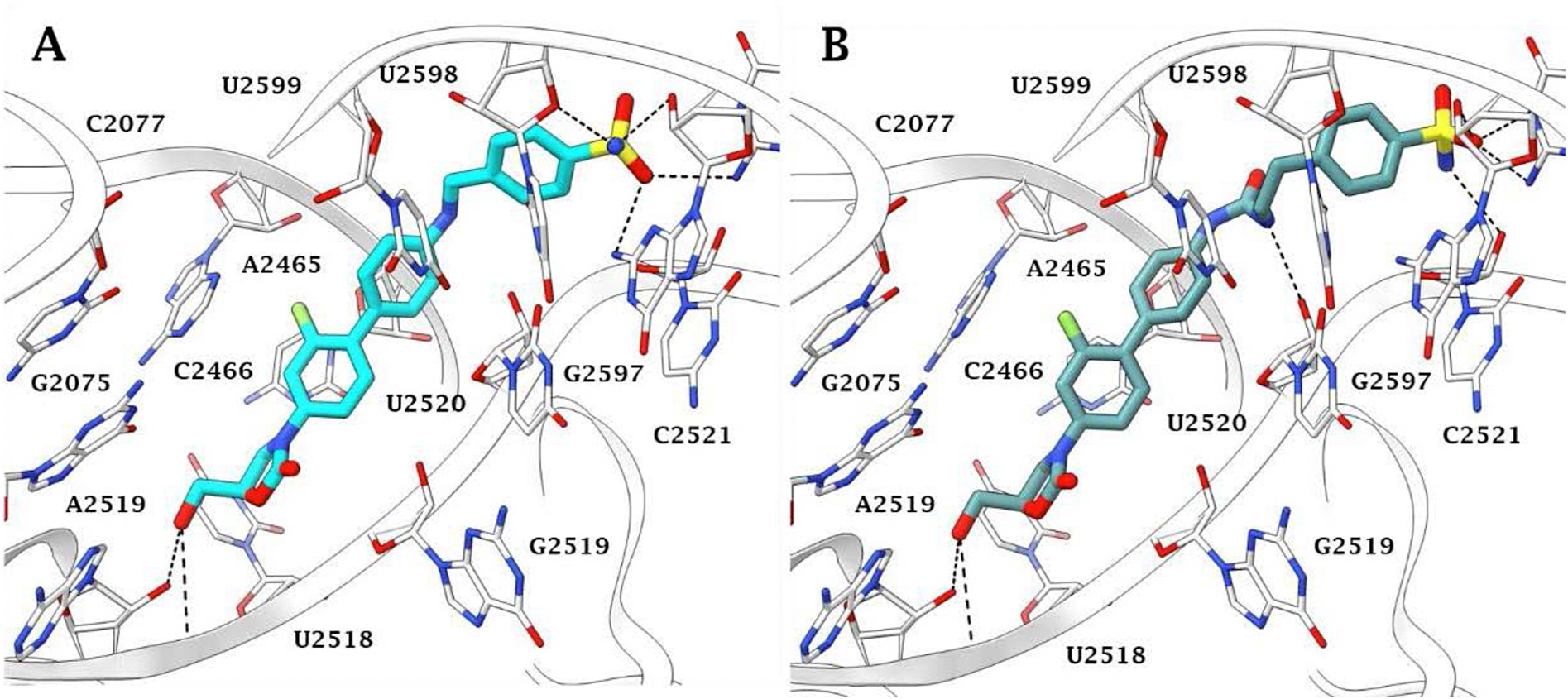
Binding mode of A) **17** (cyan) and B) **21** (green) in the PTC of the 50S ribosome subunit of *E. faecalis* predicted by docking/MM-GBSA. H-bonds are depicted as black dashed lines.

Derivatives **8**, **17**, **21** establish two key H-bonds between the oxazolidinone OH group and the ribose O2′ and phosphate OP2 atoms of A2517 and G2519 backbone, respectively and a π-π stacking interaction of ring B with C2466 cytosine core (Fig. 6A and B). Noteworthy, the sulfonamide group of all ligands is involved in contacts that reinforce the interaction with the PTC. Specifically, for compound **8**, the sulfonamide NH_2_ engages an H-bond with the backbone ribose O2′ of U2520 (Fig. 6A). Instead, the SO_2_NH_2_ moiety of derivative **17** establishes a H-bond network with the O4′, N2, O2′ and N2 atoms of U2598, G2567 and G2597, respectively (Fig. 7A). Similarly, the SO_2_NH_2_ group of **21** forms an H-bond network with the O2′, O2’, N1 and N2 atoms of C2521, G2597 and G2567, respectively. In addition, the urea group of compound **21** donates an H-bond to the ribose O2’ atom of U2520 (Fig. 7B).

The docking procedure was complemented with a 200 ns long molecular dynamic (MD) simulation performed on the predicted binding orientation of ligand **8** in the ribosomal target. The MD predicted the stability of the overall ligand positioning, whilst, specifically, the oxazolidinone core undergoes a 90° rotation approximately, guided by the target relaxation. As a result, increasing direct or water mediated H-bond interactions with the target stabilizes the binding (Fig. 6B). The ligand CO group forms water-bridged H-bond networks with the cytosine base of C2466 and with O2’ (U2518), and O3’ (G2519) and OP1 (U2520) atom set. The OH group therefore orients at H-bond distance with the phosphate group of G2519 (*i.e.* OP2 and O5’ atoms). The MD also increases the sulfonamide-involving H-bond network, that includes contacts between the NH_2_ and the O2 and O4 atoms of uridine residues 2520, 2598 and 2599.

**Fig. 8.**
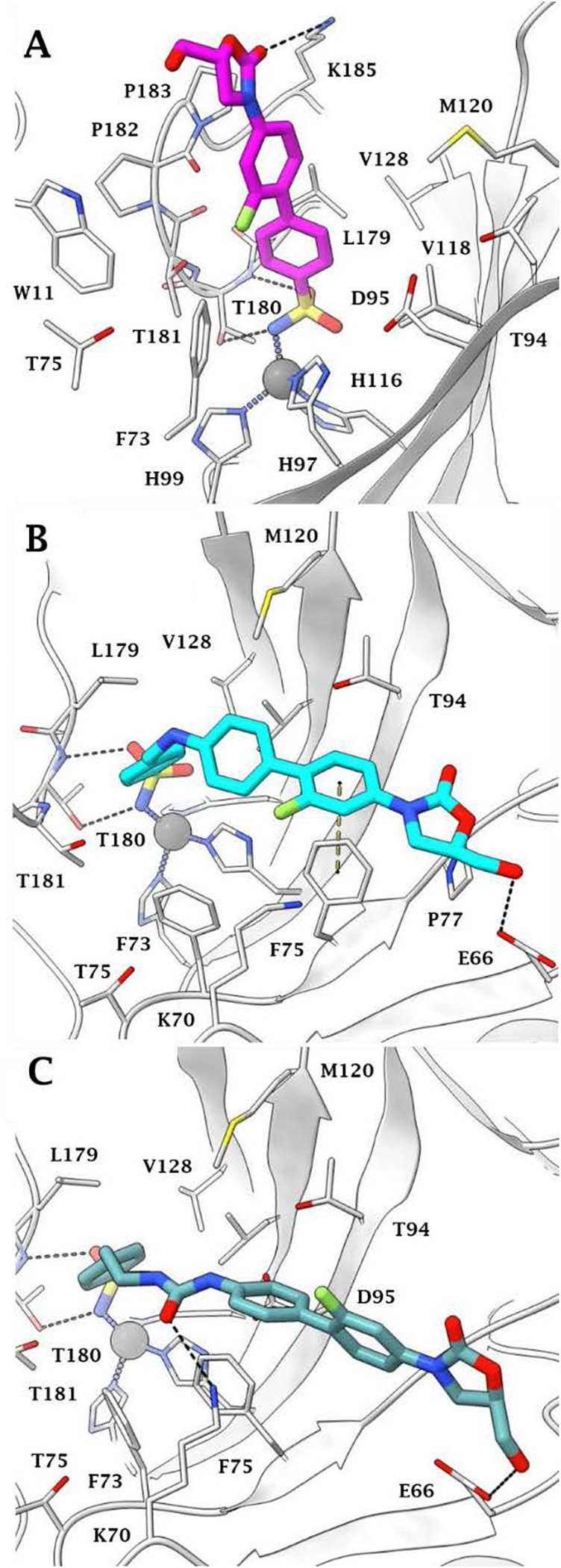
Binding mode of A) **8** (magenta), B) **17** (cyan) and C) **21** (green) in the EfCAα active site predicted by docking/MM-GBSA. H-bonds and π-contacts are depicted as black and yellow dashed lines.

**Fig. 9.**
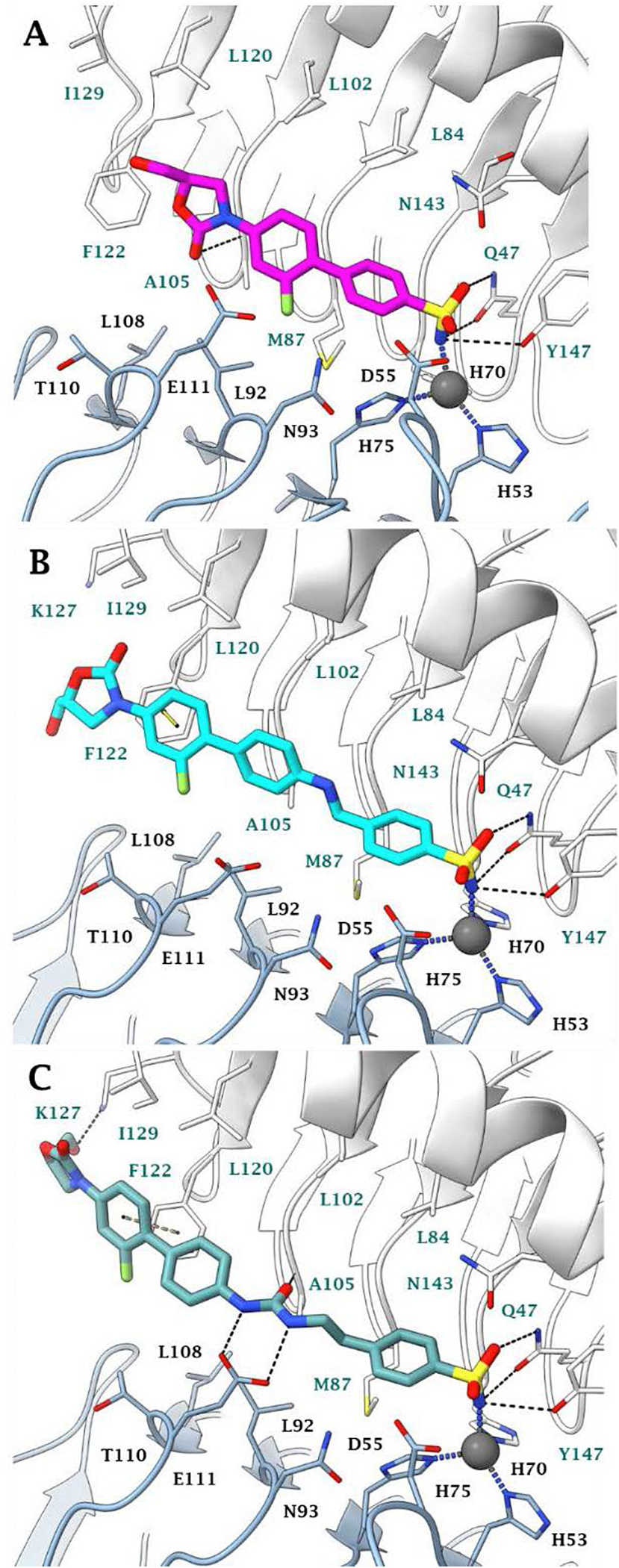
Binding mode of A) **8** (magenta), B) **17** (cyan) and C) **21** (green) in the EfCAγ active site predicted by docking/MM-GBSA. Residues of chain A and B are labeled green and black respectively. H-bonds and π-contacts are depicted as black and yellow dashed lines.

A computational simulation, based on docking followed by a MM-GBSA based refinement, was also applied to predict the binding mode of ligands **8**, **17**, **21** to the target bacterial CA isoforms belonging to *E. faecium* (EfCAα and EfCAγ) and hCA II as off-target isozyme. Being the 3D structure of EfCAα and EfCAγ not available, *ad hoc* homology models (HMs) were built and employed (Figs. S1–S6, Tables S1 and S2, Supporting Information). The ligand binding modes in bacterial CAs and hCA II showed the sulfonamide moiety bound to the zinc ion via the deprotonated nitrogen atom (SO_2_NH^−^), completing the tetragonal coordination sphere of the metal (Figs. 8 and 9 and S8, Supporting Information), which consists of three other histidine residues (H97, H99, H116 in EfCAα and H53(B), H70(A), H75(B) in EfCAγ; H94, H96, H119 in hCA II) [43]. The sulfonamide binding to the active site is further reinforced by H-bonds, respectively occurring by the S═O and NH^−^ ligand groups and the backbone NH and sidechain OH of T180 in EfCAα and T199 in hCA II. In the EfCAγ active site, the sulfonamide S═O interacts instead with the sidechain NH_2_ of Q47(A), while the NH^−^ group forms two H-bonds with the sidechain C═O group of Q47(A) and the phenolic OH of Y147(A).

The simulation conducted for ligand **8** in hCA II did not predict significant contact with the active site residues, and the fluorine atom on ring B is involved in unfavourable interactions, likely cause of its weak inhibiting potency (Fig. S8A, Supporting Information). In contrast, the hybrid compounds **17** and **21** are able to stretch out of the active site, reaching residues on the external surface (Figs. S8B–C, Supporting Information). Indeed, both derivatives establish one H-bond between the hydroxylic functionality of the oxazolidinone nucleus and the carboxylic moieties of D72 and D71, respectively. Additionally, compound **21** establishes one H-bond occurring between the urea C═O group and the sidechain NH_2_ of Q92. Nevertheless, the increased length and flexibility of the last two derivatives can possibly hamper favorable contacts with the enzyme, and moreover derivative **21** stretches towards the sterically hindered α-helix structural motif. These findings are in line with the mid-nanomolar *K*_I_ values that emerged from *in vitro* studies (262 nM, 164 nM and 63.9 nM for **8**, **17** and **21**, respectively) and could explain the lower activity of the investigated derivatives against hCA II compared to EfCAα.

The active site entrance of EfCAα is wider compared with hCA II, since the hCA II α-helix structural motif residues, ranging from 129 to 135, are replaced in EfCAα by a short coil formed by residues 121–125. The predicted pose of derivative **8** in EfCAα shows the fluorine atom on ring B oriented towards residues F73 and F75, possibly engaging hydrophobic interactions (Fig. 8A). Instead, the oxazolidinone ring is positioned close to a hydrophilic region and its C═O group establishes one H-bond with K185 (absent in hCA II). The EfCAα-**17** interaction is reinforced by π-π stacking and π-cation interactions of the ring B with F75 and K70 (absent in hCA II), respectively, and by an H-bond between the oxazolidinone OH group and the sidechain COO^−^ of E66 (Fig. 8B). This may in part rationalize its potent inhibitory activity against this bacterial isoform (*K*_I_ = 14.6 nM). Again, the binding mode of compound **21** is similar to that of derivative **17**, but its greater length also allows the ureidic linker to locate at H-bond distance with K70 (Fig. 8C).

Finally, all derivatives are able to accommodate within the wide cylindrical active site of EfCAγ extending the oxazolidinone tail towards the top of the cavity entrance. Specifically, compound **8** completely accommodates within the EfCAγ active site, establishing van der Waals (vdW) interactions between the biphenyl moiety and the lipophilic residues M87(A), L84(A), L92(B), L102(A), and A105(A) (Fig. 9A). Moreover, the oxazolidinone carbonyl group forms an H-bond with the backbone NH of A105(A). Similar hydrophobic interactions are observed for compounds **17** and **21**. For derivative **17** these contacts are reinforced by a π-π stacking between ring B and F122(A) (Fig. 9B), while compound **21** forms two such π-π stacking interactions to the same residue (Fig. 9C). Compound **21** also forms a trifurcated H-bond system occurring between the ureidic linker and the backbone NH of A105(A) and the side chain carboxylate of E111(B), whilst the ligand outer OH donates a H-bond to K127(A).

Derivatives **8**, **17**, **21**, and **24** were submitted to ADME properties calculation to investigate their drug-likeness behavior (Table 3). All compounds exhibit favorable water solubility (LogS values range from −3.54 to −5.61), with the exception of derivative **24**, which displays a LogS value of −6.90, likely due to the thioureidic moiety. In terms of human oral absorption (hOA%) and skin permeability (LogK_p_), the predictions are generally positive, with values ranging from 36.72 % to 64.89 % for hOA% and from −4.31 to 5.17 for LogKp. Notably, compounds **17** and **8** are predicted to have the highest oral absorption rates. Additionally, the passive transport across the gut-blood barrier was predicted, indicating that derivatives **8** and **17** are capable of reaching the bloodstream from enterocytes with a medium permeation level. However, compounds **21** and **24** exhibit poor permeation across this membrane, due to the presence of a higher number of rotatable bonds (n = 2).

Once entered the blood, all derivatives are easily transported to the human serum albumin with a LogK_hsa_ in the range of −0.04 to −0.52. The compound potentials to cross the blood-brain barrier and access the central nervous system are estimated by the LogBB and PMDCK descriptors, respectively. Derivatives **8** and **17** exhibits the most favorable blood/brain partition coefficient (LogBB = −2.12 and −2.80, respectively). According to the PMDCK descriptor, all compounds display poor permeability, with derivative **8** emerging as the most permeable. All compounds appear to be inactive in the central nervous system, as indicated by a CNS descriptor value of −2, which suggests a lack of psychotropic activity or CNS toxicity.The Metab descriptor revealed that all compounds are likely to undergo a single or double metabolic reaction before excretion (indicating a rapid effect).

## 3. Conclusions

The study proposes for the first time the development of oxazolidinone derivatives as inhibitors of bacterial CA isoforms to achieve new antibiotics for treating VRE infections. Dual-acting compounds are more effective against resistant strains compared to single-target drugs due to their ability to target multiple biological pathways simultaneously. This multitarget approach reduces the likelihood of developing resistance, as bacteria must adapt to multiple stressors rather than a single point of attack. Unlike single-target drugs, which often face rapid resistance development, dual-acting compounds require bacteria to undergo multiple mutations to evade their effects, making resistance less likely and slower to emerge. The class of compounds here reported merges the anti-infective mechanism of inhibiting enterococcal CAs with the well-established action of oxazolidinones (such as linezolid and congeners), which inhibit bacterial protein synthesis by binding to the peptidyl transferase center (PTC) of the ribosome. The inhibitory profile of the hybrid derivatives inspired by tedizolid and aromatic sulfonamide compounds was evaluated by a stopped-flow kinetic assay on the bacterial CAs of *E. faecalis*, using acetazolamide as a reference drug. In particular, derivatives **8** and **17** were the most potent EfCA inhibitors (*K*_I_ of 17.5 and 14.6 nM, and 63.2 and 86.9 nM, respectively) and selective compound (SI hCA I/EfCAα = 42.9 and 145; SI hCA II/EfCAα = 14.9 and 11.2; SI hCA I/EfCAγ = 11.9 and 24.9; SI hCA II/EfCAγ = 4.1 and 1.9). The compound anti-enterococcal activity was assessed against various multidrug-resistant strains of *E. faecalis*, including those oxazolidinone-resistant. Notably, compounds **8**, **17**, **21**, **24**, **25** and **26** showed 4–16-fold improved activity compared to linezolid against the oxazolidinone-resistant *E. faecalis* NR31903. Compounds **8** and **17** even outperformed the standard drugs tedizolid and acetazolamide in inhibiting the tested strains. The most promising antibiotic derivatives exhibited no significant cytotoxicity against human cell lines at concentrations up to 128 μg/mL. The ability of hybrids **8**, **17,** and **21** to bind the PTC of the *E. faecalis* 50S ribosome subunit was computationally investigated, suggesting an analog binding mode with the parent oxazolidinone drugs. Finally, a 200 ns-long MD simulation was conducted on the predicted binding orientation of compound **8** in the *E. faecalis* ribosome, revealing the overall stability of the ligand-target complex throughout the simulation, with substantial mutual adaptations and the consequent establishment of enhanced interactions. As a result, we established here a synergistic dual-target mechanistic plausibility through: (1) *in vitro* CA inhibition profiles against EfCAs; (2) joint docking/molecular dynamics simulations confirming stable binding to the PTC of the 50S subunit, consistent with known oxazolidinones. Moreover, the observed MIC values, often significantly exceeding AAZ, strongly suggests synergistic dual-target activity, as CA inhibition alone shows limited antimicrobial effects. Future research by development, and optimization of these compounds using alternative CA inhibitor (CAI) scaffolds, such as 5-substituted-1,3,4-thiadiazole-2-sulfonamides and 6-substituted-benzothiazole-2-sulfonamides may enhance our understanding of the mechanisms underlying their action and improve their efficacy against bacterial CA targets.

## 4. Materials and methods

### Chemistry.

Anhydrous solvents and all reagents were purchased from Merck Sigma, TCI and Fluorochem. All reactions involving air- or moisture-sensitive compounds were performed under a nitrogen atmosphere using dried glassware and syringes techniques to transfer solutions. Nuclear magnetic resonance (^1^H NMR, ^13^C NMR, ^19^F NMR) spectra were recorded using a Bruker Advance III 400 MHz spectrometer in DMSO‑*d*_6_. Chemical shifts are reported in parts per million (ppm) and the coupling constants (*J*) are expressed in Hertz (Hz). Splitting patterns are designated as follows: s, singlet; d, doublet; t, triplet; q, quartet; m, multiplet; dd, doublet of doublets. The assignment of exchangeable protons (O*H* and N*H*) was confirmed by the addition of D_2_O. Analytical thin-layer chromatography (TLC) was carried out on Merck silica gel F-254 plates. Flash chromatography purifications were performed on Merck Silica gel 60 (230–400 mesh ASTM) as the stationary phase and ethyl acetate/*n*-hexane or methanol/dichloromethane were used as eluents. Melting points (mp) were measured in open capillary tubes with a Gallenkamp MPD350.BM3.5 appa-ratus and are uncorrected. HRMS analysis confirmed the molecular formula of each analyte with a mass error lower than 2 ppm: a mother solution of each analyte was prepared at 1 mg/mL in methanol containing 10 % DMSO. A 20 ng/μL solution was then prepared diluting the mother solution in ACN/water containing 0.1 % formic acid and was infused in the ESI interface of the high-resolution mass spectrometer. The solution of each analyte was analyzed using a Thermo Scientific LTQ Orbitrap mass spectrometer equipped with an IonMax Electrospray interface. The high-resolution mass spectrometer operated in positive ion mode at 60000 mass resolution (at 400 *m*/*z*). Source and interface parameters were optimized on the protonated ion of each analyte: ESI voltage was 4 kV, capillary voltage 13 V, tube lens voltage 60 V; capillary temperature was 290 °C. Sheath, auxiliary and sweep gas was nitrogen, set at 15, 8, and 0 (arbitrary units), respectively. The solution was infused at 7 μL/min.

#### Synthesis of (R)-3-(4-ethynyl-3-fluorophenyl)-5-(hydroxymethyl)oxazolidin-2-one (5).

A solution of (R)-3-(3-fluoro-4-iodophenyl)-5-(hydroxymethyl)oxazolidin-2-one (**4**) (1 g, 1 eq.) in anhydrous DMF (3 ml), under nitrogen atmosphere was treated with ethynyltrimethylsilane (1.2 eq.), triethylamine (5 eq.), tetrakis(triphenylphosphine)palladium(0) (0.1 eq.) and CuI (0.1 eq.). The reaction mixture was stirred at 50 °C for 48h. After TLC monitoring, the reaction mixture was quenched with slush and extracted with EtOAc (3 × 15 mL). The combined organic layers were washed with brine (3 × 30 mL), filtered on a Celite plug, dried over anhydrous Na_2_SO_4_ and evaporated to dryness to provide a brown oil which was treated with a cold solution of TBAF 1.0 M in THF and stirred at room temperature for 1 h. After TLC monitoring, the solvent was evaporated and the residual solid treated with slush and extracted with EtOAc (3 × 15 mL). The combined organic layers were washed with brine (3 × 30 mL), dried over anhydrous Na_2_SO_4_ and evaporated to dryness to provide a light brown solid which was purified by silica gel column chromatography (EtOAc/n-hexane 50 % v/v) to afford (**5**) as an off-white solid. Yield 43 %; silica gel TLC R_f_ (EtOAc/n-hexane 70 % v/v) 0.51; *δ*_H_ (400 MHz, DMSO‑*d*_6_): 7.63 (m, 2H, Ar–H), 7.43 (m, 1H, Ar–H), 5.26 (t, *J* = 5.2 Hz, 1H, exchange with D_2_O, OH), 4.77 (m, 1H, CH), 4.46 (s, 1H, CH), 4.14 (t, *J* = 9.1 Hz, 1H, CH), 3.88 (m, 1H, CH), 3.72 (m, 1H, CH), 3.60 (m, 1H, CH); *δ*_C_ (400 MHz, DMSO‑*d*_6_): 162.40 (d, $J_{C^{-}F}^{1}=239.8\;Hz$), 154.23, 140.56 (d, ${\;J}_{C^{-}F}^{3}=10.4\;Hz$), 139.07 (d, ${\;4}_{C^{-}F}^{1}=3.8\;Hz$), 115.63, 115.21, 105.28 (d, ${\;J}_{C^{-}F}^{2}=30.3\;Hz$), 74.02, 73.77, 73.40, 61.55, 45.90; δ_f_ (400 MHz, DMSO‑*d*_6_): −111.9; HRMS (*m*/*z*) calcd. for C_12_H_10_FNO_3_ ([M+H]^+^): 236.0645, found 236.0641.

#### General procedure for the synthesis of intermediates 6, 7 and hybrid compound 8.

A solution of (R)-3-(3-fluoro-4-iodophenyl)-5-(hydroxymethyl)oxazolidin-2-one (**4**) (200 mg, 1.2 eq.) and the appropriate aryl dioxaborolane derivative (4-azidophenylboronic acid pinacol ester, 4-aminophenylboronic acid pinacol ester, **A**) (1 eq.) in 20 mL dioxane/ethanol/water (2:1:1) was treated with K_2_CO_3_ (4 eq.) and [1,1′-Bis(diphenylphosphino)ferrocene]dichloropalladium(II) (0.1 eq.). The reaction mixture was stirred at 90 °C for 5h. The solvents were removed under vacuum and the residue was treated with slush to afford a solid which was recovered by filtration and purified by silica gel chromatography, to give compounds **6**, **7**, **8**.

#### (R)-3-(4′-Azido-2-fluoro-[1,1′-biphenyl]-4-yl)-5-(hydroxymethyl)oxazolidin-2-one (6).

Compound **6** was obtained following the general procedure reported above using (R)-3-(3-fluoro-4-iodophenyl)-5-(hydroxymethyl)oxazolidin-2-one (**4**) and 2-(4-azidophenyl)-4,4,5,5-tetramethyl-1,3,2-dioxaborolane as starting materials. Pale brown solid; yield 61 %; silica gel TLC R_f_ (MeOH/DCM 5 % v/v) 0.40; *δ*_H_ (400 MHz, DMSO‑*d*_6_): 7.62 (m, 4H, Ar–H), 7.50 (m, 1H, Ar–H), 7.24 (d, *J* = 8.2 Hz, 2H, Ar–H), 5.24 (t, *J* = 5.2 Hz, 1H, exchange with D_2_O, OH), 4.76 (m, 1H, CH), 4.15 (t, *J* = 8.8 Hz, 1H, CH), 3.90 (m, 1H, CH), 3.72 (m, 1H, CH), 3.60 (m, 1H, CH); *δ*_C_ (400 MHz, DMSO‑*d*_6_): 160.13 (d, ${\;J}_{C^{-}F}^{1}=245.6\;Hz$), 154.22, 143.92, 138.12 (d, ${\;J}_{C^{-}F}^{3}=11.0\;Hz$), 128.72 (d, ${\;J}_{C^{-}F}^{4}=4.9\;Hz$), 127.98 (d, ${\;J}_{C^{-}F}^{4}=3.5\;Hz$), 126.44 (d, ${\;J}_{C^{-}F}^{3}=13.5\;Hz$), 122.78, 113.54, 113.08 (d, ${\;J}_{C^{-}F}^{4}=2.6\;Hz$), 105.53 (d, ${\;J}_{C^{-}F}^{2}=28.3\;Hz$), 73.26, 61.61, 45.88; δ_f_ (400 MHz, DMSO‑*d*_6_): −116.3; HRMS (*m*/*z*) calcd. for C_16_H_13_FN_4_O_3_ ([M+H]^+^): 329.0972, found 329.0975.

#### (R)-3-(4′-Amino-2-fluoro-[1,1′-biphenyl]-4-yl)-5-(hydroxymethyl)oxazolidin-2-one (7).

Compound **7** was obtained following general procedure reported above using (R)-3-(3-fluoro-4-iodophenyl)-5-(hydroxymethyl)oxazolidin-2-one (**4**) and 4-(4,4,5,5-tetramethyl-1,3,2-dioxaborolan-2-yl)aniline as starting materials. Orange solid; yield: 87 %; silica gel TLC R_f_ (MeOH/DCM 10 % v/v) 0.36; *δ*_H_ (400 MHz, DMSO‑*d*_6_): 7.58 (m, 1H, Ar–H), 7.49 (t, *J* = 8.7 Hz, 1H, Ar–H), 7.40 (m, 1H, Ar–H), 7.26 (m, 2H, Ar–H), 6.67 (d, *J* = 8.5 Hz, 2H, Ar–H), 5.32 (s, 2H, exchange with D_2_O, Ar-NH_2_), 5.28 (t, *J* = 5.2 Hz, 1H, exchange with D_2_O, OH), 4.76 (m, 1H, CH), 4.14 (t, *J* = 9.1 Hz, 1H, CH), 3.89 (m, 1H, CH), 3.72 (m, 1H, CH), 3.60 (m, 1H, CH); *δ*_C_ (400 MHz, DMSO‑*d*_6_): 160.02 (d, ${\;J}_{C^{-}F}^{1}=244.5\;Hz$), 154.36, 148.39, 137.91 (d, ${\;J}_{C^{-}F}^{3}=10.7\;Hz$), 129.93 (d, ${\;J}_{C^{-}F}^{4}=5.4\;Hz$), 129.20 (d, ${\;J}_{C^{-}F}^{4}=3.1\;Hz$), 123.56 (d, ${\;J}_{C^{-}F}^{3}=13.1\;Hz$), 121.64, 113.82, 113.71 (d, ${\;J}_{C^{-}F}^{4}=2.8\;Hz$), 105.55 (d, ${\;J}_{C^{-}F}^{2}=28.8\;Hz$), 73.30, 61.64, 45.97; δ_f_ (400 MHz, DMSO‑*d*_6_): −116.3; HRMS (*m*/*z*) calcd. for C_16_H_15_FN_2_O_3_ ([M+H]^+^): 303.1067, found 303.1070.

#### (R)-2′-Fluoro-4’-(5-(hydroxymethyl)-2-oxooxazolidin-3-yl)-[1,1′-biphenyl]-4-sulfonamide (8).

Compound **8** was obtained following the general procedure reported above using (R)-3-(3-fluoro-4-iodophenyl)-5-(hydroxymethyl)oxazolidin-2-one (**4**) and 4-(3,3,4,4-tetramethylborolan-1-yl)benzenesulfonamide (**A**) as starting materials. Off-white solid; yield 36 %; m.p. 241–243 °C; silica gel TLC R_f_ (MeOH/DCM 10 % v/v) 0.31; *δ*_H_ (400 MHz, DMSO‑*d*_6_): 7.95 (d, *J* = 8.1 Hz, 2H, Ar–H), 7.78 (d, *J* = 8.1 Hz, 2H, Ar–H), 7.68 (m, 2H, Ar–H), 7.53 (m, 1H, Ar–H), 7.46 (s, 2H, exchange with D_2_O, SO_2_NH_2_), 5.29 (t, *J* = 5.2 Hz, 1H, exchange with D_2_O, OH), 4.80 (m, 1H, CH), 4.18 (t, *J* = 9.1 Hz, 1H, CH), 3.93 (m, 1H, CH), 3.74 (m, 1H, CH), 3.62 (m, 1H, CH); *δ*_C_ (400 MHz, DMSO‑*d*_6_): 160.31 (d, ${\;J}_{C^{-}F}^{1}=243.9\;Hz$), 154.34, 143.07, 140.25 (d, ${\;J}_{C^{-}F}^{3}=13.1\;Hz$), 137.97, 131.03, (d, ${\;J}_{C^{-}F}^{4}=4.4\;Hz$), 129.07 (d, ${\;J}_{C^{-}F}^{4}=3.1\;Hz$), 125.97, 121.30 (d, ${\;J}_{C^{-}F}^{3}=13.2\;Hz$), 113.87, 105.46 (d, ${\;J}_{C^{-}F}^{2}=28.7\;Hz$), 73.43, 61.59, 45.97; δ_f_ (400 MHz, DMSO‑*d*_6_): −116.2; HRMS (*m*/*z*) calcd. for C_16_H_15_FN_2_O_5_S ([M+H]^+^): 367.0686, found 367.0681.

#### General procedure for the synthesis of hybrid compounds 9–15.

The appropriate alkyne (**5**, **G**-**I**) (1.2 eq.), sodium ascorbate (1 eq.) and copper(II) acetate (0.1 eq.) were added to a solution of the appropriate azide (**7**, **B**, **C**, **D**, **F**) (200 mg, 1 eq) in 10 mL MeOH/THF (1:3). The reaction mixture was stirred at 40 °C overnight. After TLC monitoring, solvents were evaporated, slush was added, and the formed precipitate was collected by filtration. The residue was redissolved in 2 mL of DMF and filtered on a Celite plug. The solvent was concentrated in vacuo, then diethyl ether was added to afford a precipitate which was collected by filtration and recrystallized from isopropanol, to give compounds **9**–**15**.

#### (R)-4-(4-(2-Fluoro-4-(5-(hydroxymethyl)-2-oxooxazolidin-3-yl) phenyl)-1H-1,2,3-triazol-1-yl)benzenesulfonamide (9).

Compound **9** was obtained following the general procedure reported above using (R)-3-(4-ethynyl-3-fluorophenyl)-5-(hydroxymethyl)oxazolidin-2-one (**5**) and 4-azidobenzenesulfonamide (**B**) as starting materials. Yellow solid; yield 80 %; m.p. >300 °C; silica gel TLC R_f_ (MeOH/DCM 10 % v/v) 0.29; *δ*_H_ (400 MHz, DMSO‑*d*_6_): 9.20 (d, *J* = 2.4 Hz, 1H, Ar–H), 8.30 (d, *J* = 8.6 Hz, 2H, Ar–H), 8.24 (t, *J* = 8.5 Hz, 1H, Ar–H), 8.08 (d, *J* = 8.6 Hz, 2H, Ar–H), 7.79 (d, *J* = 13.3 Hz, 1H, Ar–H), 7.57 (m, 3H, exchange with D_2_O, Ar–H + SO_2_NH_2_), 5.29 (t, *J* = 5.2 Hz, 1H, exchange with D_2_O, OH), 4.80 (m, 1H, CH), 4.20 (t, *J* = 9.1 Hz, 1H, CH), 3.95 (m, 1H, CH), 3.75 (m, 1H, CH), 3.63 (m, 1H, CH); *δ*_C_ (400 MHz, DMSO‑*d*_6_): 159.87 (d, ${\;J}_{C^{-}F}^{1}=246.3\;Hz$), 154.36, 143.93, 141.08, 140.13 (d, ${\;J}_{C^{-}F}^{3}=11.3\;Hz$), 138.52, 128.04 (d, ${\;J}_{C^{-}F}^{4}=4.4\;Hz$), 127.47, 121.36 (d, ${\;J}_{C^{-}F}^{3}=10.2\;Hz$), 120.55, 113.84 (d, ${\;J}_{C^{-}F}^{4}=2.6\;Hz$), 112.35 (d, ${\;J}_{C^{-}F}^{3}=13.8\;Hz$), 105.30 (d, ${\;J}_{C^{-}F}^{2}=27.2\;Hz$), 73.46, 61.61, 46.00; δ_f_ (400 MHz, DMSO‑*d*_6_): −111.8; HRMS (*m*/*z*) calcd. for C_18_H_16_FN_5_O_5_S ([M+H]^+^): 434.0856, found 434.0859.

#### (R)-4-((4-(2-Fluoro-4-(5-(hydroxymethyl)-2-oxooxazolidin-3-yl) phenyl)-1H-1,2,3-triazol-1-yl)methyl)benzenesulfonamide (10).

Compound **10** was obtained following the general procedure reported above using (R)-3-(4-ethynyl-3-fluorophenyl)-5-(hydroxymethyl)oxazolidin-2-one (**5**) and 4-(azidomethyl)benzenesulfonamide (**C**) as starting materials. Pale yellow solid; yield 83 %; m.p. >300 °C; silica gel TLC R_f_ (MeOH/DCM 10 % v/v) 0.34; *δ*_H_ (400 MHz, DMSO‑*d*_6_): 8.59 (d, *J* = 3.3 Hz, 1H, Ar–H), 8.16 (t, *J* = 8.6 Hz, 1H, Ar–H), 7.87 (d, *J* = 8.0 Hz, 2H, Ar–H), 7.73 (d, *J* = 13.3 Hz, 1H, Ar–H), 7.57 (d, *J* = 8.0 Hz, 2H, Ar–H), 7.50 (d, *J* = 8.7 Hz, 1H, Ar–H), 7.41 (s, 2H, exchange with D_2_O, SO_2_NH_2_), 5.82 (s, 2H, CH_2_), 5.28 (t, *J* = 5.2 Hz, 1H, exchange with D_2_O, OH), 4.78 (m, 1H, CH), 4.17 (t, *J* = 9.1 Hz, 1H, CH), 3.92 (m, 1H, CH), 3.73 (m, 1H, CH), 3.61 (m, 1H, CH); *δ*_C_ (400 MHz, DMSO‑*d*_6_): 159.64 (d, $J_{C^{-}F}^{1}=243.4\;Hz$), 154.31, 143.82, 139.83, 139.73, 139.62 (d, ${\;J}_{C^{-}F}^{3}=11.4\;Hz$), 128.39, 127.59 (d, ${\;J}_{C^{-}F}^{4}=4.7\;Hz$), 126.14, 123.67 (d, ${\;J}_{C^{-}F}^{3}=10.7\;Hz$), 113.75 (d, ${\;J}_{C^{-}F}^{4}=2.1\;Hz$), 112.96 (d, ${\;J}_{C^{-}F}^{3}=13.8\;Hz$), 105.20 (d, ${\;J}_{C^{-}F}^{2}=27.4\;Hz$), 73.39, 61.58, 52.29, 45.96; δ_f_ (400 MHz, DMSO‑*d*_6_): −112.9; HRMS (*m*/*z*) calcd. for C_19_H_18_FN_5_O_5_S ([M+H]^+^): 448.1013, found 448.1017.

#### (R)-4-(2-(4-(2-Fluoro-4-(5-(hydroxymethyl)-2-oxooxazolidin-3-yl)phenyl)-1H-1,2,3-triazol-1-yl)ethyl)benzenesulfonamide (11).

Compound **11** was obtained following the general procedure reported above using (R)-3-(4-ethynyl-3-fluorophenyl)-5-(hydroxymethyl)oxazolidin-2-one (**5**) and 4-(2-azidoethyl)benzenesulfonamide (**D**) as starting materials. Pale yellow solid; yield 88 %; m.p. >300 °C; silica gel TLC R_f_ (MeOH/DCM 10 % v/v) 0.39; *δ*_H_ (400 MHz, DMSO‑*d*_6_): 8.46 (s, 1H, Ar–H), 8.14 (t, *J* = 7.9 Hz, 1H, Ar–H), 7.77 (d, *J* = 7.2 Hz, 2H, Ar–H), 7.72 (d, *J* = 13.6 Hz, 1H, Ar–H), 7.48 (m, 3H, Ar–H), 7.35 (s, 2H, exchange with D_2_O, SO_2_NH_2_), 5.29 (t, *J* = 5.2 Hz, 1H, exchange with D_2_O, OH), 4.77 (m, 3H, CH_2_ + CH), 4.17 (t, *J* = 9.1 Hz, 1H, CH), 3.91 (m, 1H, CH), 3.72 (m, 1H, CH), 3.63 (m, 1H, CH), 3.36 (t, *J* = 9.1 Hz, 2H, CH_2_, overlapped by H_2_O signal); *δ*_C_ (400 MHz, DMSO‑*d*_6_): 159.57 (d, ${\;J}_{C^{-}F}^{1}=245.2\;Hz$), 154.30, 142.48, 141.79, 139.49 (d, ${\;J}_{C^{-}F}^{3}=9.9\;Hz$), 139.38, 129.20, 127.52 (d, ${\;J}_{C^{-}F}^{4}=5.2\;Hz$), 125.72, 123.25 (d, ${\;J}_{C^{-}F}^{3}=10.7\;Hz$), 113.74 (d, ${\;J}_{C^{-}F}^{4}=2.1\;Hz$), 113.13 (d, ${\;J}_{C^{-}F}^{3}=13.9\;Hz$), 105.22 (d, ${\;J}_{C^{-}F}^{2}=27.1\;Hz$), 73.37, 61.57, 50.05, 45.95, 35.23; δ_f_ (400 MHz, DMSO‑*d*_6_): −113.0; HRMS (*m*/*z*) calcd. for C_20_H_20_FN_5_O_5_S ([M+H]^+^): 462.1169, found 462.1165.

#### (R)-2-(4-(2-Fluoro-4-(5-(hydroxymethyl)-2-oxooxazolidin-3-yl) phenyl)-1H-1,2,3-triazol-1-yl)-N-(5-sulfamoyl-1,3,4-thiadiazol-2-yl)acetamide (12).

Compound **12** was obtained following the general procedure reported above using (R)-3-(4-ethynyl-3-fluorophenyl)-5-(hydroxymethyl)oxazolidin-2-one (**5**) and 2-azido-N-(5-sulfamoyl-1,3,4-thiadiazol-2-yl)acetamide (**F**) as starting materials. Yellow solid; yield 75 %; m.p. >300 °C; silica gel TLC R_f_ (MeOH/DCM 10 % v/v) 0.29; *δ*_H_ (400 MHz, DMSO‑*d*_6_): 13.73 (bs, 1H, exchange with D_2_O, CONH) 8.50 (s, 1H, Ar–H), 8.22 (m, 3H, exchange with D_2_O, Ar–H + SO_2_NH_2_), 7.74 (d, *J* = 13.8 Hz, 1H, Ar–H), 7.51 (d, *J* = 8.0 Hz, 1H, Ar–H), 5.60 (s, 2H, CH_2_), 5.30 (t, *J* = 5.2 Hz, 1H, exchange with D_2_O, OH), 4.78 (m, 1H, CH), 4.20 (t, *J* = 9.1 Hz, 1H, CH), 3.94 (m, 1H, CH), 3.75 (m, 1H, CH), 3.63 (m, 1H, CH); *δ*_C_ (400 MHz, DMSO‑*d*_6_): 167.64, 163.51, 159.64 (d, ${\;J}_{C^{-}F}^{1}=246.0\;Hz$), 154.34, 139.57, (d, ${\;J}_{C^{-}F}^{3}=11.7\;Hz$), 139.41, 137. 92, 127.53 (d, ${\;J}_{C^{-}F}^{4}=4.8\;Hz$), 124.97 (d, ${\;J}_{C^{-}F}^{3}=10.7\;Hz$), 113.82 (d, ${\;J}_{C^{-}F}^{4}=1.9\;Hz$), 113.06 (d, ${\;J}_{C^{-}F}^{3}=13.8\;Hz$), 105.26 (d, ${\;J}_{C^{-}F}^{2}=27.7\;Hz$), 73.42, 61.58, 52.46, 46.00; δ_f_ (400 MHz, DMSO‑*d*_6_): −113.1; HRMS (*m*/*z*) calcd. for C_16_H_15_FN_8_O_6_S_2_ ([M+H]^+^): 499.0540, found 499.0535.

#### (R)-4-((1-(2′-Fluoro-4’-(5-(hydroxymethyl)-2-oxooxazolidin-3-yl)-[1,1′-biphenyl]-4-yl)-1H-1,2,3-triazol-4-yl)methoxy)benzenesulfonamide (13).

Compound **13** was obtained following the general procedure reported above using (R)-3-(4′-azido-2-fluoro-[1,1′-biphenyl]-4-yl)-5-(hydroxymethyl)oxazolidin-2-one (**6**) and 4-(prop-2-yn-1-yloxy)benzenesulfonamide (**G**) as starting materials. Off-White solid; yield 72 %; m.p. >300 °C; silica gel TLC R_f_ (MeOH/DCM 10 % v/v) 0.23; *δ*_H_ (400 MHz, DMSO‑*d*_6_): 9.10 (s, 1H, Ar–H), 8.07 (d, *J* = 8.6 Hz, 2H, Ar–H), 7.83 (m, 4H, Ar–H), 7.71 (m, 2H, Ar–H), 7.54 (d, *J* = 8.3 Hz, 1H, Ar–H), 7.29 (m, 4H, exchange with D_2_O, Ar–H + SO_2_NH_2_), 5.40 (s, 1H, CH_2_), 5.29 (t, *J* = 5.2 Hz, 1H, exchange with D_2_O, OH), 4.80 (m, 1H, CH), 4.19 (t, *J* = 9.1 Hz, 1H, CH), 3.94 (m, 1H, CH), 3.75 (m, 1H, CH), 3.63 (m, 1H, CH); *δ*_C_ (400 MHz, DMSO‑*d*_6_): 160.27 (d, ${\;J}_{C^{-}F}^{1}=243.2\;Hz$), 154.33, 143.40, 139.96 (d, ${\;J}_{C^{-}F}^{3}=11.2\;Hz$), 136.63, 135.68, 135.00, 130.87 (d, ${\;J}_{C^{-}F}^{4}=4.9\;Hz$), 129.99 (d, ${\;J}_{C^{-}F}^{4}=3.5\;Hz$), 127.68, 123.06, 121.34 (d, ${\;J}_{C^{-}F}^{3}=13.7\;Hz$), 121.18, 120.32, 114.81, 113.87 (d, ${\;J}_{C^{-}F}^{4}=2.6\;Hz$), 105.50 (d, ${\;J}_{C^{-}F}^{2}=28.8\;Hz$), 73.41, 61.59, 61.31, 45.97; δ_f_ (400 MHz, DMSO‑*d*_6_): −116.2; HRMS (*m*/*z*) calcd. for C_25_H_22_FN_5_O_6_S ([M+H]^+^): 540.1275, found 540.1270.

#### (R)-4-(((1-(2′-Fluoro-4’-(5-(hydroxymethyl)-2-oxooxazolidin-3-yl)-[1,1′-biphenyl]-4-yl)-1H-1,2,3-triazol-4-yl)methyl)amino)benzenesulfonamide (14).

Compound **14** was obtained following the general procedure reported above using (R)-3-(4′-azido-2-fluoro-[1,1′-biphenyl]-4-yl)-5-(hydroxymethyl)oxazolidin-2-one (**6**) and 4-(prop-2-yn-1-yl amino)benzenesulfonamide (**H**) as starting materials. Off-White solid; yield 88 %; m.p. >300 °C; silica gel TLC R_f_ (MeOH/DCM 10 % v/v) 0.18; *δ*_H_ (400 MHz, DMSO‑*d*_6_): 8.85 (s, 1H, Ar–H), 8.04 (d, *J* = 8.3 Hz, 2H, Ar–H), 7.81 (d, *J* = 8.3 Hz, 2H, Ar–H), 7.70 (m, 2H, Ar–H), 7.57 (d, *J* = 8.5 Hz, 2H, Ar–H), 7.53 (d, *J* = 8.3 Hz, 1H, Ar–H), 7.29 (m, 3H, exchange with D_2_O, NH + SO_2_NH_2_), 6.79 (d, *J* = 8.5 Hz, 2H, Ar–H), 5.31 (t, *J* = 5.2 Hz, 1H, exchange with D_2_O, OH), 4.80 (m, 1H, CH), 4.50 (d, *J* = 5.4 Hz, 2H, CH_2_), 4.19 (t, *J* = 9.1 Hz, 1H, CH), 3.94 (m, 1H, CH), 3.74 (m, 1H, CH), 3.61 (m, 1H, CH); *δ*_C_ (400 MHz, DMSO‑*d*_6_): 160.28 (d, ${\;J}_{C^{-}F}^{3}=244.1\;Hz$), 154.33, 150.88, 146.09, 139.92 (d, ${\;J}_{C^{-}F}^{3}=11.4\;Hz$), 135.80, 134.77, 130.83 (d, ${\;J}_{C^{-}F}^{4}=4.4\;Hz$), 130.69, 129.96 (d, ${\;J}_{C^{-}F}^{4}=3.1\;Hz$), 127.29, 121.36 (d, ${\;J}_{C^{-}F}^{3}=11.4\;Hz$), 121.23, 120.08, 113.86 (d, ${\;J}_{C^{-}F}^{4}=2.3\;Hz$), 111.20, 105.50 (d, ${\;J}_{C^{-}F}^{2}=28.8\;Hz$), 73.41, 61.60, 45.97; 37.99; δ_f_ (400 MHz, DMSO‑*d*_6_): −116.2; HRMS (*m*/*z*) calcd. for C_25_H_23_FN_6_O_5_S ([M+H]^+^): 539.1435, found 539.1430.

#### (R)-N-((1-(2′-Fluoro-4’-(5-(hydroxymethyl)-2-oxooxazolidin-3-yl)-[1,1′-biphenyl]-4-yl)-1H-1,2,3-triazol-4-yl)methyl)-4-sulfamoylbenzamide (15).

Compound **15** was obtained following the general procedure reported above using (R)-3-(4′-azido-2-fluoro-[1,1′-biphenyl]-4-yl)-5-(hydroxymethyl)oxazolidin-2-one (**6**) and N-(prop-2-yn-1-yl)-4-sulfamoylbenzamide (**I**) as starting materials. Off-White solid; yield 74 %; m.p. >300 °C; silica gel TLC R_f_ (MeOH/DCM 10 % v/v) 0.24; *δ*_H_ (400 MHz, DMSO‑*d*_6_): 9.35 (t, *J* = 5.3 Hz, 1H, exchange with D_2_O, CONH), 8.83 (s, 1H, Ar–H), 8.09 (m, 4H, Ar–H), 7.95 (d, *J* = 8.5 Hz, 2H, Ar–H), 7.81 (d, *J* = 7.9 Hz, 2H, Ar–H), 7.70 (m, 2H, Ar–H), 7.54 (m, 3H, exchange with D_2_O, Ar–H + SO_2_NH_2_), 5.31 (t, *J* = 5.2 Hz, 1H, exchange with D_2_O, OH), 4.80 (m, 1H, CH), 4.69 (d, *J* = 5.3 Hz, 2H, CH_2_), 4.19 (t, *J* = 9.1 Hz, 1H, CH), 3.93 (m, 1H, CH), 3.74 (m, 1H, CH), 3.63 (m, 1H, CH); *δ*_C_ (400 MHz, DMSO‑*d*_6_): 165.24, 160.29 (d, ${\;J}_{C^{-}F}^{1}=246.1\;Hz$), 154.34, 146.35, 146.04, 139.92 (d, ${\;J}_{C^{-}F}^{3}=11.1\;Hz$), 137.02, 135.83, 134.74, 130.85 (d, ${\;J}_{C^{-}F}^{4}=5.0\;Hz$), 129.94 (d, ${\;J}_{C^{-}F}^{4}=3.3\;Hz$), 128.04, 125.59, 121.37 (d, ${\;J}_{C^{-}F}^{3}=12.8\;Hz$), 121.21, 120.07, 113.87 (d, ${\;J}_{C^{-}F}^{4}=3.0\;Hz$), 105.50 (d, ${\;J}_{C^{-}F}^{2}=28.1\;Hz$), 73.41, 61.60, 45.97; 34.95; δ_f_ (400 MHz, DMSO‑*d*_6_): −116.2; HRMS (*m*/*z*) calcd. for C_26_H_23_FN_6_O_6_S ([M+H]^+^): 567.1384, found 567.1388.

#### (R)-N-(2′-Fluoro-4’-(5-(hydroxymethyl)-2-oxooxazolidin-3-yl)-[1,1′-biphenyl]-4-yl)-4-sulfamoylbenzamide (16).

A solution of (R)-3-(4′-amino-2-fluoro-[1,1′-biphenyl]-4-yl)-5-(hydroxymethyl)oxazolidin-2-one (**7**) (200 mg, 1.2 eq.) in anhydrous DMF (2 mL), under nitrogen atmosphere, was treated with pyridine (2 eq.), 4-sulfamoylbenzoyl chloride (**J**) (1 eq.) and stirred at room temperature overnight. After TLC monitoring, the reaction was quenched with slush and HCl 1 M to afford a precipitate that was collected by filtration, washed with diethyl ether and purified by silica gel column chromatography (MeOH/DCM 10 % v/v). White solid; yield 55 %; m.p. 254–256 °C; silica gel TLC R_f_ (MeOH/DCM 10 % v/v) 0.30; *δ*_H_ (400 MHz, DMSO‑*d*_6_): 10.58 (s, 1H, exchange with D_2_O, CONH), 8.16 (d, *J* = 8.0 Hz, 2H, Ar–H), 8.00 (d, *J* = 8.0 Hz, 2H, Ar–H), 7.93 (d, *J* = 8.2 Hz, 2H, Ar–H), 7.60 (m, 6H, exchange with D_2_O, Ar–H + SO_2_NH_2_), 7.48 (d, *J* = 8.3 Hz, 1H, Ar–H), 5.27 (t, *J* = 5.2 Hz, 1H, exchange with D_2_O, OH), 4.78 (m, 1H, CH), 4.17 (t, *J* = 8.8 Hz, 1H, CH), 3.92 (m, 1H, CH), 3.72 (m, 1H, CH), 3.61 (m, 1H, CH); *δ*_C_ (400 MHz, DMSO‑*d*_6_): 164.58, 160.23 (d, ${\;J}_{C^{-}F}^{1}=244.9\;Hz$), 154.35, 146.58, 139.22 (d, ${\;J}_{C^{-}F}^{3}=11.7\;Hz$), 138.39, 137.74, 130.62 (d, ${\;J}_{C^{-}F}^{4}=5.0\;Hz$), 130.11, 128.88 (d, ${\;J}_{C^{-}F}^{4}=2.7\;Hz$), 128.39, 125.68, 122.34 (d, ${\;J}_{C^{-}F}^{3}=13.3\;Hz$), 120.40, 113.81 (d, ${\;J}_{C^{-}F}^{4}=2.7\;Hz$), 105.51 (d, ${\;J}_{C^{-}F}^{2}=29.0\;Hz$), 73.37, 61.61, 45.98; δ_f_ (400 MHz, DMSO‑*d*_6_): −116.4; HRMS (*m*/*z*) calcd. for C_23_H_20_FN_3_O_6_S ([M+H]^+^): 486.1057, found 486.1061.

#### (R)-4-(((2′-Fluoro-4’-(5-(hydroxymethyl)-2-oxooxazolidin-3-yl)-[1,1′-biphenyl]-4-yl)amino)methyl)benzenesulfonamide (17).

A solution of (R)-3-(4′-amino-2-fluoro-[1,1′-biphenyl]-4-yl)-5-(hydroxymethyl)oxazolidin-2-one (**7**) (200 mg, 1 eq.) in anhydrous DMF (2 mL), under nitrogen atmosphere, was treated with K_2_CO_3_ (2 eq.), 4-(bromomethyl)benzenesulfonamide (**K**) (1.2 eq.) and stirred at 60 °C overnight. After TLC monitoring, the reaction was quenched with slush to afford a precipitate that was collected by filtration, washed with diethyl ether and purified by silica gel column chromatography (MeOH/DCM 10 % v/v). Off-White solid; yield 41 %; m.p. 236–238 °C; silica gel TLC R_f_ (MeOH/DCM 10 % v/v) 0.27; *δ*_H_ (400 MHz, DMSO‑*d*_6_): 7.83 (m, 2H, Ar–H), 7.55 (m, 4H, exchange with D_2_O, Ar–H + NH), 7.33 (m, 6H, exchange with D_2_O, Ar–H + SO_2_NH_2_), 6.67 (d, *J* = 8.0 Hz, 2H, Ar–H), 5.27 (t, *J* = 5.2 Hz, 1H, exchange with D_2_O, OH), 4.77 (m, 1H, CH), 4.44 (d, *J* = 5.4 Hz, 2H, CH_2_), 4.14 (t, *J* = 8.8 Hz, 1H, CH), 3.88 (m, 1H, CH), 3.72 (m, 1H, CH), 3.60 (m, 1H, CH); *δ*_C_ (400 MHz, DMSO‑*d*_6_): 160.04 (d, $J_{C^{-}F}^{1}=245.2\;Hz$), 154.39, 147.87, 144.58, 142.51, 138.05 (d, ${\;J}_{C^{-}F}^{3}=11.2\;Hz$), 130.02 (d, ${\;J}_{C^{-}F}^{4}=4.8\;Hz$), 129.27 (d, ${\;J}_{C^{-}F}^{4}=1.9\;Hz$), 127.38, 125.80, 123.34, (d, ${\;J}_{C^{-}F}^{3}=13.0\;Hz$), 122.08, 113.75 (d, ${\;J}_{C^{-}F}^{4}=2.8\;Hz$), 122.36, 105.55 (d, ${\;J}_{C^{-}F}^{2}=28.9\;Hz$), 73.33, 61.65, 45.98, 45.83; δ_f_ (400 MHz, DMSO‑*d*_6_): −116.7; HRMS (*m*/*z*) calcd. for C_23_H_22_FN_3_O_5_S ([M+H]^+^): 472.1264, found 472.1259.

#### General procedure for the synthesis of hybrid compounds 18–21.

The appropriate carbamate (**L**-**O**) (1 eq.) and triethylamine (0.1 eq) were added to a solution of (R)-3-(4′-amino-2-fluoro-[1,1′-biphenyl]-4-yl)-5-(hydroxymethyl)oxazolidin-2-one (**7**) (150 mg, 1.1 eq) in anhydrous acetonitrile (15 mL) under nitrogen atmosphere. The reaction mixture was stirred at reflux temperature overnight. After TLC monitoring, the reaction mixture was quenched with slush and HCl 1 M, the readily formed precipitate was collected by filtration and purified by silica gel chromatography column to afford compounds **18**–**21**.

#### (R)-5-(3-(2′-Fluoro-4’-(5-(hydroxymethyl)-2-oxooxazolidin-3-yl)-[1,1′-biphenyl]-4-yl)ureido)-1,3,4-thiadiazole-2-sulfonamide (18).

Compound **18** was obtained following the general procedure reported above using (R)-3-(4′-amino-2-fluoro-[1,1′-biphenyl]-4-yl)-5-(hydroxymethyl)oxazolidin-2-one (**7**) and phenyl (5-sulfamoyl-1,3,4-thiadiazol-2-yl)carbamate (**L**) as starting materials. Off-White solid; yield 37 %; m.p. 250–252 °C; silica gel TLC R_f_ (MeOH/DCM 10 % v/v) 0.23; *δ*_H_ (400 MHz, DMSO‑*d*_6_): 11.55 (s, 1H, exchange with D_2_O, CONH), 9.38 (s, 1H, exchange with D_2_O, CONH), 8.33 (s, 2H, exchange with D_2_O, SO_2_NH_2_), 7.65 (m, 3H, Ar–H), 7.59 (m, 3H, Ar–H), 7.48 (m, 1H, Ar–H), 5.28 (t, *J* = 5.2 Hz, 1H, exchange with D_2_O, OH), 4.79 (m, 1H, CH), 4.18 (t, *J* = 8.8 Hz, 1H, CH), 3.92 (m, 1H, CH), 3.74 (m, 1H, CH), 3.61 (m, 1H, CH); *δ*_C_ (400 MHz, DMSO‑*d*_6_): 167.74, 163.51, 160.22 (d, ${\;J}_{C^{-}F}^{1}=242.9\;Hz$), 154.39, 152.04, 139.19 (d, ${\;J}_{C^{-}F}^{3}=10.2\;Hz$), 137.83, 130.62 (d, ${\;J}_{C^{-}F}^{4}=5.2\;Hz$), 129.43, 129.15 (d, ${\;J}_{C^{-}F}^{4}=2.8\;Hz$), 122.31 (d, ${\;J}_{C^{-}F}^{3}=13.0\;Hz$), 119.13, 113.82 (d, ${\;J}_{C^{-}F}^{4}=2.8\;Hz$), 105.52 (d, ${\;J}_{C^{-}F}^{2}=29.1\;Hz$), 73.40, 61.63, 45.99; δ_f_ (400 MHz, DMSO‑*d*_6_): −116.5; HRMS (*m*/*z*) calcd. for C_19_H_17_FN_6_O_6_S_2_ ([M+H]^+^): 509.0635, found 509.0639.

#### (R)-4-(3-(2′-Fluoro-4’-(5-(hydroxymethyl)-2-oxooxazolidin-3-yl)-[1,1′-biphenyl]-4-yl)ureido)benzenesulfonamide (19).

Compound **19** was obtained following the general procedure reported above using (R)-3-(4′-amino-2-fluoro-[1,1′-biphenyl]-4-yl)-5-(hydroxymethyl) oxazolidin-2-one (**7**) and phenyl (4-sulfamoylphenyl)carbamate (**M**) as starting materials. White solid; yield 53 %; m.p. 271–273 °C; silica gel TLC R_f_ (MeOH/DCM 10 % v/v) 0.30; *δ*_H_ (400 MHz, DMSO‑*d*_6_): 9.15 (s, 1H, exchange with D_2_O, CONH), 8.99 (s, 1H, exchange with D_2_O, CONH), 7.78 (d, *J* = 8.7 Hz, 2H, Ar–H), 7.63 (m, 6H, Ar–H), 7.53 (d, *J* = 8.1 Hz, 2H, Ar–H), 7.47 (m, 1H, Ar–H), 7.25 (s, 2H, exchange with D_2_O, SO_2_NH_2_), 5.28 (t, *J* = 5.2 Hz, 1H, exchange with D_2_O, OH), 4.78 (m, 1H, CH), 4.17 (t, *J* = 9.1 Hz, 1H, CH), 3.91 (m, 1H, CH), 3.74 (m, 1H, CH), 3.62 (m, 1H, CH); *δ*_C_ (400 MHz, DMSO‑*d*_6_): 160.19 (d, ${\;J}_{C^{-}F}^{1}=243.9\;Hz$), 154.37, 152.22, 142.78, 139.01 (d, ${\;J}_{C^{-}F}^{3}=9.8\;Hz$), 138.90, 136.90, 130.53 (d, ${\;J}_{C^{-}F}^{4}=5.0\;Hz$), 129.07 (d, ${\;J}_{C^{-}F}^{4}=2.9\;Hz$), 128.33, 126.85, 122.48 (d, ${\;J}_{C^{-}F}^{3}=13.6\;Hz$), 118.41, 117.50, 113.79 (d, ${\;J}_{C^{-}F}^{4}=2.8\;Hz$), 105.51 (d, ${\;J}_{C^{-}F}^{2}=29.7\;Hz$), 73.37, 61.62, 45.97; δ_f_ (400 MHz, DMSO‑*d*_6_): −116.5; HRMS (*m*/*z*) calcd. for C_23_H_21_FN_4_O_6_S ([M+H]^+^): 501.1166, found 501.1160.

#### (R)-4-((3-(2′-Fluoro-4’-(5-(hydroxymethyl)-2-oxooxazolidin-3-yl)-[1,1′-biphenyl]-4-yl)ureido)methyl)benzenesulfonamide (20).

Compound **20** was obtained following the general procedure reported above using (R)-3-(4′-amino-2-fluoro-[1,1′-biphenyl]-4-yl)-5-(hydroxymethyl)oxazolidin-2-one (**7**) and phenyl (4-sulfamoylbenzyl)carbamate (**N**) as starting materials. Off-white solid; yield 74 %; m.p. 264–266 °C; silica gel TLC R_f_ (MeOH/DCM 10 % v/v) 0.25; *δ*_H_ (400 MHz, DMSO‑*d*_6_): 8.79 (s, 1H, exchange with D_2_O, CONH), 7.81 (d, *J* = 7.4 Hz, 2H, Ar–H), 7.52 (m, 9H, Ar–H), 7.31 (s, 2H, exchange with D_2_O, SO_2_NH_2_), 6.78 (bs, 1H, exchange with D_2_O, CONH), 5.23 (t, *J* = 5.2 Hz, 1H, exchange with D_2_O, OH), 4.75 (m, 1H, CH), 4.41 (d, *J* = 4.7 Hz, 2H, CH_2_), 4.14 (t, *J* = 9.1 Hz, 1H, CH), 3.89 (m, 1H, CH), 3.72 (m, 1H, CH), 3.59 (m, 1H, CH); *δ*_C_ (400 MHz, DMSO‑*d*_6_): 160.19 (d, ${\;J}_{C^{-}F}^{1}=245.3\;Hz$), 155.20, 154.38, 144.64, 142.54, 139.99, 138.85 (d, ${\;J}_{C^{-}F}^{3}=9.8\;Hz$), 130.46 (d, ${\;J}_{C^{-}F}^{4}=4.6\;Hz$), 128.93 (d, ${\;J}_{C^{-}F}^{4}=2.3\;Hz$), 127.34, 125.73, 122.91, 122.67 (d, ${\;J}_{C^{-}F}^{3}=13.1\;Hz$), 117.79, 113.78, (d, ${\;J}_{C^{-}F}^{4}=2.8\;Hz$), 105.52 (d, ${\;J}_{C^{-}F}^{2}=28.4\;Hz$), 73.37, 61.63, 45.98, 42.43; δ_f_ (400 MHz, DMSO‑*d*_6_): −116.5; HRMS (*m*/*z*) calcd. for C_24_H_23_FN_4_O_6_S ([M+H]^+^): 515.1322, found 515.1318.

#### (R)-4-(2-(3-(2′-Fluoro-4’-(5-(hydroxymethyl)-2-oxooxazolidin-3-yl)-[1,1′-biphenyl]-4-yl)ureido)ethyl)benzenesulfonamide (21).

Compound **21** was obtained following the general procedure reported above using (R)-3-(4′-amino-2-fluoro-[1,1′-biphenyl]-4-yl)-5-(hydroxymethyl)oxazolidin-2-one (**7**) and phenyl (4-sulfamoylphenethyl) carbamate (**O**) as starting materials. Off-white solid; yield 81 %; m.p. 258–261 °C; silica gel TLC R_f_ (MeOH/DCM 10 % v/v) 0.30; *δ*_H_ (400 MHz, DMSO‑*d*_6_): 8.67 (s, 1H, exchange with D_2_O, CONH), 7.81 (d, *J* = 8.2 Hz, 2H, Ar–H), 7.51 (m, 9H, Ar–H), 7.34 (s, 2H, exchange with D_2_O, SO_2_NH_2_), 6.23 (t, *J* = 5.2 Hz, 1H, exchange with D_2_O, CONH), 5.27 (bs, 1H, exchange with D_2_O, OH), 4.78 (m, 1H, CH), 4.16 (t, *J* = 9.1 Hz, 1H, CH), 3.91 (m, 1H, CH), 3.73 (m, 1H, CH), 3.61 (m, 1H, CH), 3.43 (m, 2H, CH_2_), 2.86 (t, *J* = 6.9 Hz, 2H, CH_2_); *δ*_C_ (400 MHz, DMSO‑*d*_6_): 160.16 (d, ${\;J}_{C^{-}F}^{1}=245.1\;Hz$), 155.06, 154.37, 143.83, 142.07, 140.08, 138.81 (d, $J_{C^{-}F}^{3}=10.5\;Hz$), 130.43 (d, ${\;J}_{C^{-}F}^{4}=5.0\;Hz$), 129.19, 128.91 (d, ${\;J}_{C^{-}F}^{4}=2.2\;Hz$), 127.15, 125.75, 122.69, (d, $J_{C^{-}F}^{3}=12.9\;Hz$), 117.60, 113.77 (d, ${\;J}_{C^{-}F}^{4}=2.4\;Hz$), 105.52 (d, ${\;J}_{C^{-}F}^{2}=28.9\;Hz$), 73.36, 61.63, 45.97, 43.59, 35.54; δ_f_ (400 MHz, DMSO‑*d*_6_): −116.6; HRMS (*m*/*z*) calcd. for C_25_H_25_FN_4_O_6_S ([M+H]^+^): 529.1479, found 529.1482.

#### General procedure for the synthesis of hybrid compounds 22–26.

The appropriate isothiocyanate (**P**-**T**) (1 eq.) and triethylamine (0.1 eq) were added to a solution of (R)-3-(4′-amino-2-fluoro-[1,1′-biphenyl]-4-yl)-5-(hydroxymethyl)oxazolidin-2-one (**7**) (150 mg, 1.1 eq) in anhydrous acetonitrile (15 mL), under nitrogen atmosphere. The reaction mixture was stirred at room temperature overnight. After TLC monitoring, the reaction mixture was quenched with slush and HCl 1 M, the readily formed precipitate was collected by filtration and purified by silica gel chromatography column to afford compounds **22**–**26**.

#### (R)-4-(3-(2′-Fluoro-4’-(5-(hydroxymethyl)-2-oxooxazolidin-3-yl)-[1,1′-biphenyl]-4-yl)thioureido)benzenesulfonamide (22).

Compound **22** was obtained following the general procedure reported above using (R)-3-(4′-amino-2-fluoro-[1,1′-biphenyl]-4-yl)-5-(hydroxymethyl)oxazolidin-2-one (**7**) and 4-isothiocyanatobenzenesulfonamide (**P**) as starting materials. White solid; yield 57 %; m.p. 248–251 °C; silica gel TLC R_f_ (MeOH/DCM 10 % v/v) 0.25; *δ*_H_ (400 MHz, DMSO‑*d*_6_): 10.21 (d, *J* = 3.0 Hz, 2H, exchange with D_2_O, 2xCSNH), 7.81 (d, *J* = 8.8 Hz, 2H, Ar–H), 7.75 (d, *J* = 8.8 Hz, 2H, Ar–H), 7.62 (m, 6H, Ar–H), 7.50 (m, 1H, Ar–H), 7.35 (s, 2H, exchange with D_2_O, SO_2_NH_2_), 5.29 (t, *J* = 5.2 Hz, 1H, exchange with D_2_O, OH), 4.79 (m, 1H, CH), 4.18 (t, *J* = 9.0 Hz, 1H, CH), 3.92 (m, 1H, CH), 3.74 (m, 1H, CH), 3.62 (m, 1H, CH); *δ*_C_ (400 MHz, DMSO‑*d*_6_): 179.51, 160.28 (d, ${\;J}_{C^{-}F}^{1}=244.9\;Hz$), 154.41, 142.72, 139.35 (d, ${\;J}_{C^{-}F}^{3}=10.3\;Hz$), 139.12, 138.74, 130.84, 130.73 (d, ${\;J}_{C^{-}F}^{4}=4.1\;Hz$), 128.79 (d, 4.1 Hz), 126.22, 123.62, 122.71, 122.30 (d, $J_{C^{-}F}^{3}=12.7\;Hz$), 113.88 (d, ${\;J}_{C^{-}F}^{4}=2.9\;Hz$), 105.56 (d, ${\;J}_{C^{-}F}^{2}=29.1\;Hz$), 73.43, 61.65, 46.00; δ_f_ (400 MHz, DMSO‑*d*_6_): −116.4; HRMS (*m*/*z*) calcd. for C_23_H_21_FN_4_O_5_S_2_ ([M+H]^+^): 517.0937, found 517.0941.

#### (R)-4-((3-(2′-Fluoro-4’-(5-(hydroxymethyl)-2-oxooxazolidin-3-yl)-[1,1′-biphenyl]-4-yl)thioureido)methyl)benzenesulfonamide (23).

Compound **23** was obtained following the general procedure reported above using (R)-3-(4′-amino-2-fluoro-[1,1′-biphenyl]-4-yl)-5-(hydroxymethyl)oxazolidin-2-one (**7**) and 4-(isothiocyanatomethyl) benzenesulfonamide (**Q**) as starting materials. White solid; yield 63 %; m.p. 234–236 °C; silica gel TLC R_f_ (MeOH/DCM 10 % v/v) 0.28; *δ*_H_ (400 MHz, DMSO‑*d*_6_): 9.90 (s, 1H, exchange with D_2_O, CSNH), 8.43 (s, 1H, exchange with D_2_O, CSNH), 7.83 (d, *J* = 8.9 Hz, 2H, Ar–H), 7.57 (m, 9H, Ar–H), 7.37 (s, 2H, exchange with D_2_O, SO_2_NH_2_), 5.29 (t, *J* = 5.2 Hz, 1H, exchange with D_2_O, OH), 4.86 (d, *J* = 5.3 Hz, 2H, CH_2_), 4.78 (m, 1H, CH), 4.17 (t, *J* = 8.4 Hz, 1H, CH), 3.92 (m, 1H, CH), 3.73 (m, 1H, CH), 3.61 (m, 1H, CH); *δ*_C_ (400 MHz, DMSO‑*d*_6_): 180.96, 160.20 (d, ${\;J}_{C^{-}F}^{1}=244.0\;Hz$), 154.32, 143.24, 142.56, 139.25 (d, ${\;J}_{C^{-}F}^{3}=11.2\;Hz$), 138.57, 130.59 (d, ${\;J}_{C^{-}F}^{4}=5.0\;Hz$), 130.46, 128.82 (d, ${\;J}_{C^{-}F}^{4}=2.4\;Hz$), 127.54, 125.60, 123.28, 122.26 (d, ${\;J}_{C^{-}F}^{3}=13.3\;Hz$), 113.82 (d, ${\;J}_{C^{-}F}^{4}=2.7\;Hz$), 105.52 (d, ${\;J}_{C^{-}F}^{2}=28.8\;Hz$), 73.35, 61.60, 46.72, 45.96; δ_f_ (400 MHz, DMSO‑*d*_6_): −116.3; HRMS (*m*/*z*) calcd. for C_24_H_23_FN_4_O_5_S_2_ ([M+H]^+^): 531.1094, found 531.1098.

#### (R)-4-(2-(3-(2′-Fluoro-4’-(5-(hydroxymethyl)-2-oxooxazolidin-3-yl)-[1,1′-biphenyl]-4-yl)thioureido)ethyl)benzenesulfonamide (24).

Compound **24** was obtained following the general procedure reported above using (R)-3-(4′-amino-2-fluoro-[1,1′-biphenyl]-4-yl)-5-(hydroxymethyl)oxazolidin-2-one (**7**) and 4-(2-isothiocyanatoethyl) benzenesulfonamide (**R**) as starting materials. Off-white solid; yield 89 %; m.p. 238–240 °C; silica gel TLC R_f_ (MeOH/DCM 10 % v/v) 0.31; *δ*_H_ (400 MHz, DMSO‑*d*_6_): 9.75 (s, 1H, exchange with D_2_O, CSNH), 7.94 (s, 1H, exchange with D_2_O, CSNH), 7.82 (d, *J* = 8.1 Hz, 2H, Ar–H), 7.56 (m, 9H, Ar–H), 7.36 (s, 2H, exchange with D_2_O, SO_2_NH_2_), 5.29 (t, *J* = 5.2 Hz, 1H, exchange with D_2_O, OH), 4.79 (m, 1H, CH), 4.17 (t, *J* = 8.4 Hz, 1H, CH), 3.92 (m, 1H, CH), 3.75 (m, 3H, CH + CH_2_), 3.62 (m, 1H, CH), 3.02 (t, *J* = 7.1 Hz, 2H, CH_2_); *δ*_C_ (400 MHz, DMSO‑*d*_6_): 180.29, 160.24 (d, ${\;J}_{C^{-}F}^{1}=243.9\;Hz$), 154.38, 143.59, 142.18, 139.25 (d, ${\;J}_{C^{-}F}^{3}=11.0\;Hz$), 138.64, 130.65 (d, ${\;J}_{C^{-}F}^{4}=4.4\;Hz$), 130.24, 129.21, 128.86 (d, ${\;J}_{C^{-}F}^{4}=2.1\;Hz$), 125.81, 122.90, 122.32, (d, $J_{C^{-}F}^{3}=13.0\;Hz$), 113.85 (d, ${\;J}_{C^{-}F}^{4}=2.3\;Hz$), 105.53 (d, ${\;J}_{C^{-}F}^{2}=28.4\;Hz$), 73.40, 61.64, 45.98, 44.96, 34.16; δ_f_ (400 MHz, DMSO‑*d*_6_): −116.4; HRMS (*m*/*z*) calcd. for C_25_H_25_FN_4_O_5_S_2_ ([M+H]^+^): 545.1250, found 545.1254.

#### (R)-3-Chloro-4-(3-(2′-fluoro-4’-(5-(hydroxymethyl)-2-oxooxazolidin-3-yl)-[1,1′-biphenyl]-4-yl)thioureido)benzenesulfonamide (25).

Compound **25** was obtained following the general procedure reported above using (R)-3-(4′-amino-2-fluoro-[1,1′-biphenyl]-4-yl)-5-(hydroxymethyl)oxazolidin-2-one (**7**) and 3-chloro-4-isothiocyanatobenzenesulfonamide (**S**) as starting materials. Yellow solid; yield 44 %; m.p. 261–264 °C; silica gel TLC R_f_ (MeOH/DCM 10 % v/v) 0.21; *δ*_H_ (400 MHz, DMSO‑*d*_6_): 10.38 (s, 1H, exchange with D_2_O, CSNH), 9.71 (s, 1H, exchange with D_2_O, CSNH), 7.93 (d, *J* = 7.8 Hz, 2H, Ar–H), 7.60 (m, 11H, exchange with D_2_O, Ar–H + SO_2_NH_2_), 5.23 (t, *J* = 5.2 Hz, 1H, exchange with D_2_O, OH), 4.76 (bs, 1H, CH), 4.15 (t, *J* = 8.8 Hz, 1H, CH), 3.90 (m, 1H, CH), 3.72 (m, 1H, CH), 3.60 (m, 1H, CH); *δ*_C_ (400 MHz, DMSO‑*d*_6_): 179.96, 160.23 (d, ${\;J}_{C^{-}F}^{1}=244.5\;Hz$), 154.33, 142.10, 139.56, 139.34 (d, ${\;J}_{C^{-}F}^{3}=11.7\;Hz$), 138.44, 131.03, 130.65 (d, ${\;J}_{C^{-}F}^{4}=4.4\;Hz$), 129.42, 129.32, 128.76 (d, ${\;J}_{C^{-}F}^{4}=2.5\;Hz$), 126.67, 124.39, 123.64, 122.20 (d, $J_{C^{-}F}^{3}=13.6\;Hz$), 113.83 (d, $J_{C^{-}F}^{4}=3.1\;Hz$), 105.52 (d, ${\;J}_{C^{-}F}^{2}=28.8\;Hz$), 73.36, 61.61, 45.96; δ_f_ (400 MHz, DMSO‑*d*_6_): −116.3; HRMS (*m*/*z*) calcd. for C_23_H_20_ClFN_4_O_5_S_2_ ([M+H]^+^): 551.0548, found 551.0545.

#### (R)-3-(3-(2′-Fluoro-4’-(5-(hydroxymethyl)-2-oxooxazolidin-3-yl)-[1,1′-biphenyl]-4-yl)thioureido)benzenesulfonamide (26).

Compound **26** was obtained following the general procedure reported above using (R)-3-(4′-amino-2-fluoro-[1,1′-biphenyl]-4-yl)-5-(hydroxymethyl)oxazolidin-2-one (**7**) and 3-isothiocyanatobenzenesulfonamide (**T**) as starting materials. White solid; yield 46 %; m.p. 233–235 °C; silica gel TLC R_f_ (MeOH/DCM 10 % v/v) 0.26; *δ*_H_ (400 MHz, DMSO‑*d*_6_): 10.10 (s, 1H, exchange with D_2_O, CSNH), 8.02 (s, 1H, exchange with D_2_O, CSNH), 7.76 (d, *J* = 7.5 Hz, 1H, Ar–H), 7.55 (m, 10H, Ar–H), 7.40 (s, 2H, exchange with D_2_O, SO_2_NH_2_), 5.24 (bs, 1H, exchange with D_2_O, OH), 4.76 (m, 1H, CH), 4.15 (t, *J* = 8.8 Hz, 1H, CH), 3.90 (m, 1H, CH), 3.71 (m, 1H, CH), 3.59 (m, 1H, CH); *δ*_C_ (400 MHz, DMSO‑*d*_6_): 179.73, 160.24 (d, ${\;J}_{C^{-}F}^{1}=244.2\;Hz$), 154.35, 144.25, 140.08, 139.31 (d, ${\;J}_{C^{-}F}^{3}=11.7\;Hz$), 138.71, 130.75, 130.67 (d, ${\;J}_{C^{-}F}^{4}=4.8\;Hz$), 128.95, 128.75 (d, ${\;J}_{C^{-}F}^{4}=2.5\;Hz$), 126.78, 123.56, 122.28 (d, ${\;J}_{C^{-}F}^{3}=13.7\;Hz$), 121.35, 120.49, 113.84 (d, ${\;J}_{C^{-}F}^{4}=2.8\;Hz$), 105.53 (d, ${\;J}_{C^{-}F}^{2}=28.9\;Hz$), 73.38, 61.62, 45.97; δ_f_ (400 MHz, DMSO‑*d*_6_): −116.4; HRMS (*m*/*z*) calcd. for C_23_H_21_FN_4_O_5_S_2_ ([M+H]^+^): 517.0937, found 517.0934.

#### Carbonic anhydrase CO_2_ hydration catalytic assay and K_I_ determination.

The assay was performed according to previously published protocols [22]. Recombinant EfCAα and EfCAγ from *E. faecium* [23] and purchased hCAs I and II (from Merck Sigma) were used in the biochemical inhibition assays. *K*_I_ values were determined from inputting the IC_50_ values into the Cheng-Prusoff equation for the *K*_I_ from catalytic inhibition constants.

#### Determination of the minimum inhibitory concentrations (MICs).

The MICs of the oxazolidinone-sulfonamide hybrid compounds and control drugs were determined using the broth microdilution method, according to guidelines outlined by the Clinical and Laboratory Standards Institute (CLSI) [43–46]. Briefly, a bacterial solution equivalent to 0.5 McFarland standard was prepared and diluted to achieve a bacterial concentration of about 5 × 10^5^ CFU/mL and seeded in 96-well well plates. Serial dilutions of test agents were then incubated with bacteria at 37 °C either aerobically or in the presence of 5 % CO_2_ for 18–20 h. MICs were recorded as the minimum concentrations of test agents that completely inhibited bacterial growth.

#### *In silico* study.

The primary sequences of EfCAα and EfCAγ were retrieved from the UniProt Consortium [47]. The crystal structures of α-CA from *Photobacterium profundum* (PDB 5HPJ; resolution 1.50 Å) [48] and γ-CA from *Escherichia coli* (PDB 3TIO; resolution 1.41 Å) [49] were used as template in the homology modeling procedure (sequence alignment is reported in Figs. S1 and S2, Supporting Information). Multiple models were generated using the Prime module of Schrödinger [50] and the SwissModel platform [51] and submitted to loop refinements and quality evaluation procedures (Figs. S3–7, Tables S1 and S2, Supporting Information). The best-scored structures of EfCAα and EfCAγ and the crystal structure of CA II (PDB 3K34) [44] and the 50S subunit of the *E. faecalis* ribosome (PDB 6WU9) [41] downloaded by Protein Data Bank (RCSB.org) [52] were prepared using the Protein Preparation module implemented in Maestro Schrödinger suite [50], assigning bond orders, adding hydrogens, deleting water molecules, and optimizing H-bonding networks. Finally, energy minimization with a Root Means Square Deviation (RMSD) value of 0.30 was applied using an Optimized Potential for Liquid Simulation (OPLS4) force field [53] The 3D ligand structures were prepared by Maestro [50] and evaluated for their ionization states at pH 7.3 ± 1.0 with Epik. The conjugate gradient method in Macromodel [50] was used for energy minimization (maximum iteration number: 2500; convergence criterion: 0.05 kcal/mol/Å^2^). Grids for docking were centered in the centroid of the complexed ligand. Docking studies were carried out with the program Glide using the standard precision (SP) mode. The best poses for each compound were re-docked and scored for its binding free energies (dG bind) with the Prime MM-GBSA protocol using a VSGB solvation model [50]. Molecular dynamics (MD) simulations were performed using Desmond Molecular Dynamics System (v.7.9) and OPLS4 force field. All systems were solvated in an orthorhombic box using simple point charge water molecules extended 15 Å away from any protein atom. The system was neutralized with 0.15 M Cl^−^ and Na^+^ ions. The simulation protocol included a starting relaxation step followed by a final production phase of 200 ns. In particular, the relaxation step comprised the following: (a) a stage of 100 ps at 10 K retaining the harmonic restraints on the solute heavy atoms (force constant of 50 kcal/mol/Å2) using the NPT ensemble with Brownian dynamics; (b) a stage of 12 ps at 10 K with harmonic restraints on the solute heavy atoms (force constant of 50 kcal/mol/Å^2^), using the NVT ensemble and Berendsen thermostat; (c) a stage of 12 ps at 10 K and 1 atm, retaining the harmonic restraints and using the NPT ensemble and Berendsen thermostat and barostat; (f) a stage of 12 ps at 300 K and 1 atm, retaining the harmonic restraints and using the NPT ensemble and Berendsen thermostat and barostat; (g) a final 24 ps stage at 300 K and 1 atm without harmonic restraints, using the NPT Berendsen thermostat and barostat. The final production phase of MD was run using a canonical NPT Berendsen ensemble at 300 K. During the MD simulation, a time step of 2 fs was used while constraining the bond lengths of H atoms with the M-SHAKE algorithm. The atomic coordinates of the system were saved every 100 ps along the MD trajectory. Protein RMSD, ligand RMSD/RMSF (Root Mean Square Fluctuation) ligand torsions evolution and occurrence of intermolecular H-bonds and hydrophobic contacts were provided by the Simulation Interaction Diagram (SID) implemented in Maestro along with the production phase of the MD simulation. The tool reads the MD trajectory file and identifies ligand/target interactions repeatedly occurring during the simulation time. Figures were generated with Chimera [54]. The compound ADMET parameters were predicted by the QikProp module implemented in Schrodinger [50].

#### In-cell study.

Compounds **8**, **17**, **21** and **24** were assayed at concentrations of 32, 64, and 128 μg/mL against human epithelial kidney (HEK293) cells and human colorectal adenocarcinoma (Caco-2) cells, as described previously [55–59]. Briefly, cells were incubated with the compounds (in triplicates) in 96-well plates at 37 °C for 24 h. Control cells received DMSO at a concentration equal to that of the compounds-treated wells. MTS (3-(4,5-dimethylthiazol-2-yl)-5-(3-carboxymethoxyphenyl)-2-(4-sulfophenyl)-2H-tetrazolium) (Promega, Madison, WI) was subsequently added and the plates were incubated for 3 h. Absorbance readings (at OD_490_) were recorded using a kinetic microplate reader. Cells viability after treatment of each compound was expressed as a percentage of the viability relative to DMSO-treated control cells. The data were analyzed via two-way ANOVA with post-hoc Dunnett’s test for multiple comparisons.

## Supplementary Material

SI

Appendix A. Supplementary data

Supplementary data to this article can be found online at.

Appendix B. Supplementary data

Supplementary data to this article can be found online at https://doi.org/10.1016/j.ejmech.2025.117620.

## Figures and Tables

**Table 1:** Inhibition data of human CA isoforms I, II, and *E. faecium* CA isoforms EfCAα and EfCAγ for compounds **8–26** by a CO_2_ hydrase stopped-flow assay using acetazolamide (AAZ) as a reference inhibitor.[40]

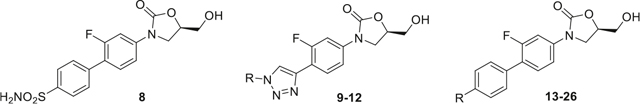					

Cmpd	R	K_I_ (nM)^a^			
	
		hCA I	hCA II	EfCAα	EfCAγ

**8**	–	750 ± 38	262 ± 16	17.5 ± 0.9	63.2 ± 4.2
**9**	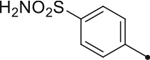	845 ± 46	98.4 ± 4.9	136 ± 7	471 ± 26
**10**	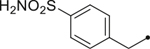	1460 ± 80	54.9 ± 3.2	200 ± 13	520 ± 28
**11**	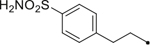	298 ± 18	73.2 ± 4.2	295 ± 15	199 ± 15
**12**	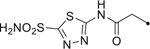	1690 ± 90	117 ± 8	598 ± 34	686 ± 33
**13**	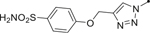	2240 ± 130	175 ± 12	91.6 ± 5.9	80.1 ± 4.5
**14**	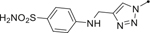	2420 ± 160	89.7 ± 6.2	270 ± 18	216 ± 14
**15**	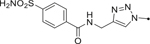	3480 ± 190	139 ± 10	76.2 ± 3.6	112 ± 10
**16**	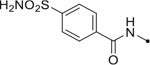	1330 ± 80	54.7 ± 2.8	329 ± 21	798 ± 38
**17**	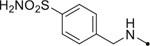	2130 ± 130	164 ± 10	14.6 ± 1.0	86.9 ± 5.5
**18**	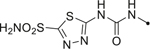	1810 ± 100	40.6 ± 2.3	409 ± 39	141 ± 11
**19**	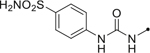	2030 ± 120	32.6 ± 1.2	169 ± 14	91.3 ± 5.6
**20**	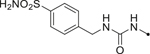	4190 ± 220	84.1 ± 4.6	94.8 ± 4.7	120 ± 9
**21**	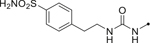	1970 ± 100	63.9 ± 3.6	58.3 ± 2.9	99.1 ± 5.7
**22**	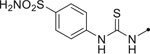	3050 ± 160	19.4 ± 0.9	82.0 ± 6.3	364 ± 26
**23**	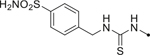	2010 ± 120	36.1 ± 2.0	280 ± 20	500 ± 42
**24**	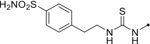	979 ± 49	121 ± 6	87.2 ± 4.2	112 ± 5
**25**	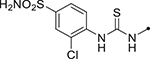	814 ± 63	103 ± 8	148 ± 11	92.4 ± 5.8
**26**	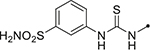	3620 ± 190	95.1 ± 5.8	71.0 ± 3.9	541 ± 28
**AAZ**	–	250 ± 16	12.0 ± 1.1	56.7 ± 3.2	323 ± 27

a Inhibition data are expressed as means ± SEM of three different assays.

**Table 2:** MICs (μg/mL) of oxazolidinone-sulfonamide hybrids against oxazolidinone-sensitive VRE strains (*E. faecalis* NR 31971, *E. faecalis* NR 31972, *E. faecium* HM-965) and the oxazolidinone-resistant VRE strain (*E. faecalis* NR 31903).

Cmpd	Enterococcal strains			
	
	*E. faecalis* NR 31971	*E. faecalis* NR 31972	*E. faecium* HM-965	*E. faecalis* NR 31903

**8**	0.25	0.25	0.125	1
**9**	>64	>64	>64	nt
**10**	>64	>64	>64	nt
**11**	32	32	>64	nt
**12**	64	32	32	nt
**13**	0.125	0.125	0.125	>64
**14**	4	4	2	>64
**15**	2	1	1	>64
**16**	64	64	32	nt
**17**	1	1	0.5	1
**18**	2	2	1	>64
**19**	1	1	1	>64
**20**	1	1	0.5	>64
**21**	1	1	0.5	2
**22**	8	8	2	>64
**23**	>64	>64	>64	nt
**24**	1	1	1	2
**25**	8	8	4	4
**26**	2	2	1	4
**AAZ**	2	2	1	2
**LNZ**	1	1	1	16
**TDZ**	0.25	0.25	1	4
**Vancomycin**	64	>64	>64	>64

AAZ: acetazolamide, LNZ: linezolid, TDZ: tedizolid; nt: not tested.

**Table 3:** QikProp predicted properties for the derivatives **8**, **17**, **21**, and **24** compared with the standard oxazolidinones, tedizolid (**TDZ**) and linezolid (**LNZ**), and acetazolamide (**AAZ**) as reference CAI.

Cmpd	LogS^a^	hOA %^b^	LogK_p_^c^	PCaco^d^	logK_hsa_^e^	LogBB^f^	PMDCK^g^	CNS^h^	Metab^i^

**8**	−3.54	57.85	−4.91	41.75	−0.52	−2.12	24.75	−2	2
**17**	−5.41	64.89	−4.31	33.57	−0.14	−2.8	19.55	−2	1
**21**	−5.61	36.72	−5.17	7.4	−0.37	−3.71	5.78	−2	2
**24**	−6.9	49.64	−4.78	17.82	−0.04	−3.41	19.48	−2	1
**AAZ**	−1.56	45.03	−5.88	37.72	−0.97	−1.73	25.23	−2	1
**TDZ**	−4.1	70.11	−4.3	111.19	−0.42	−1.65	69.67	−2	1
**LNZ**	−2.08	77.36	−3.11	456.83	−0.65	−0.56	605.27	−1	3

a LogS (predicted aqueous solubility expressed in mol/L) must be in the range of −6.5 – 0.5.

b hOA % (predicted percent human oral absorption) is poor for values < 25 % and high for values > 85 %.

c LogK_p_ (prediction skin permeability) must be in the range of −8.0 to −1.0.

d PCaco (apparent Caco-2 cell permeability in nm/sec) judges Caco-2 cells as a good model to study the passive transport across the gut-blood barrier, considering a poor permeation for values < 25 and high permeation for values > 500.

e LogK_hsa_ (prediction of binding to human serum albumin) must be in the range of −1.5 – 1.5.

f LogBB (predicted brain/blood partition coefficient) must be in the range of −3.0 – 1.2.

g PMDCK (predicted apparent MDCK cell permeability in nm/sec) judges MDCK cells as a good model to study the passive transport across the blood-brain barrier, considering a poor permeation for values < 25 and high permeation for values > 500.

h CNS (predicted central nervous system activity) ranges molecules on a scale from −2 (inactive) to +2 (active).

i Metab (number of likely metabolic reactions) must be in the range of 1–8.

## Data Availability

Data will be made available on request.
