# Supplementary material for: Redesigning oxazolidinones as carbonic anhydrase inhibitors against vancomycin-resistant enterococci
Source: Eur J Med Chem. Author manuscript; Available in PMC 2026 Jul 5. (PMC12186203; doi:10.1016/j.ejmech.2025.117620)

**Supplementary data**

**Redesigning Oxazolidinones as Carbonic Anhydrase Inhibitors against Vancomycin-Resistant Enterococci.**

Andrea Ammara,^a,b^ Simone Giovannuzzi,^a*^ Alessandro Bonardi,^b^ Nader S Abutaleb,^c,d^ Ahmed A. Abouelkhair,^c,d^ Daniel P. Flaherty,^e,f,g^ Mohamed N. Seleem,^c,d^ Clemente Capasso,^h^ Paola Gratteri,^b^ Alessio Nocentini,^a*^ Claudiu T. Supuran^a^

a. Neurofarba Department, Pharmaceutical and Nutraceutical Section, University of Florence, Sesto Fiorentino, Italy.

b. NEUROFARBA Department, Laboratory of Molecular Modeling, Cheminformatics & QSAR, University of Florence, Firenze, Italy.

c. Department of Biomedical Sciences and Pathobiology, Virginia-Maryland College of Veterinary Medicine, Virginia Polytechnic Institute and State University, Blacksburg, VA, USA.

d. Center for One Health Research, Virginia Polytechnic Institute and State University, Blacksburg, VA, USA.

e. Department of Medicinal Chemistry and Molecular Pharmacology, College of Pharmacy, Purdue University, West Lafayette, IN, USA.

f. Purdue Institute for Drug Discovery, West Lafayette, IN, USA.

g. Purdue Institute of Inflammation, Immunology and Infectious Disease, West Lafayette, IN, USA.

h. Istituto di Bioscienze e Biorisorse, CNR, 80131 Naples, Italy

email: [simone.giovannuzzi@unifi.it](mailto:simone.giovannuzzi@unifi.it); [alessio.nocentini@unifi.it](mailto:alessio.nocentini@unifi.it)

| **Table of contents** | **Page** |
| --- | --- |
|  |  |
| Structure of intermediate derivatives **A-R**. | S2 |
| Sequence alignment, 3D structures and parameters of EfCAα and EfCAγ homology-built models | S3-S6 |
| Docking figure with hCA II | S7 |
| Synthetic procedures and characterizations of intermediates | S8 |
| ^1^H, ^13^C and ^19^F spectra of derivatives **8**-**26**. | S9-S37 |

**Figure S1.** Structure of intermediate derivatives **A-R**.


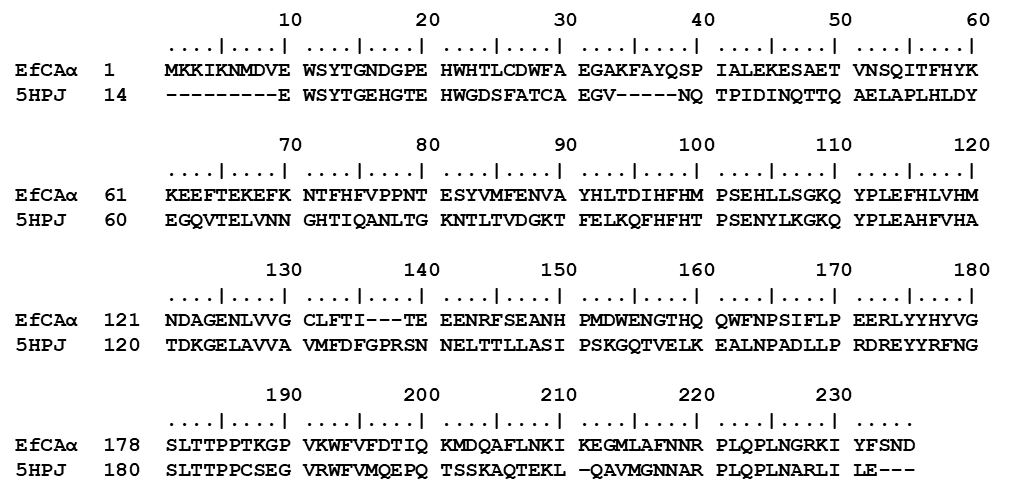


**Figure S2.** Sequence alignment of EfCAα with the template α-CA from *Photobacterium profundum* (PDB 5HPJ).


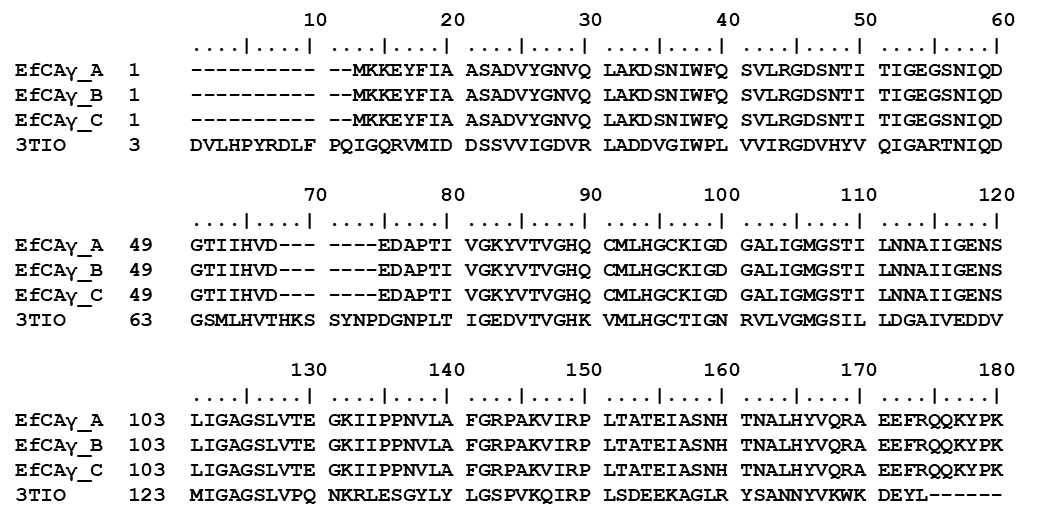


**Figure S3.** Sequence alignment of EfCAγ with the template γ-CA from *Escherichia coli* (PDB 3TIO).

**
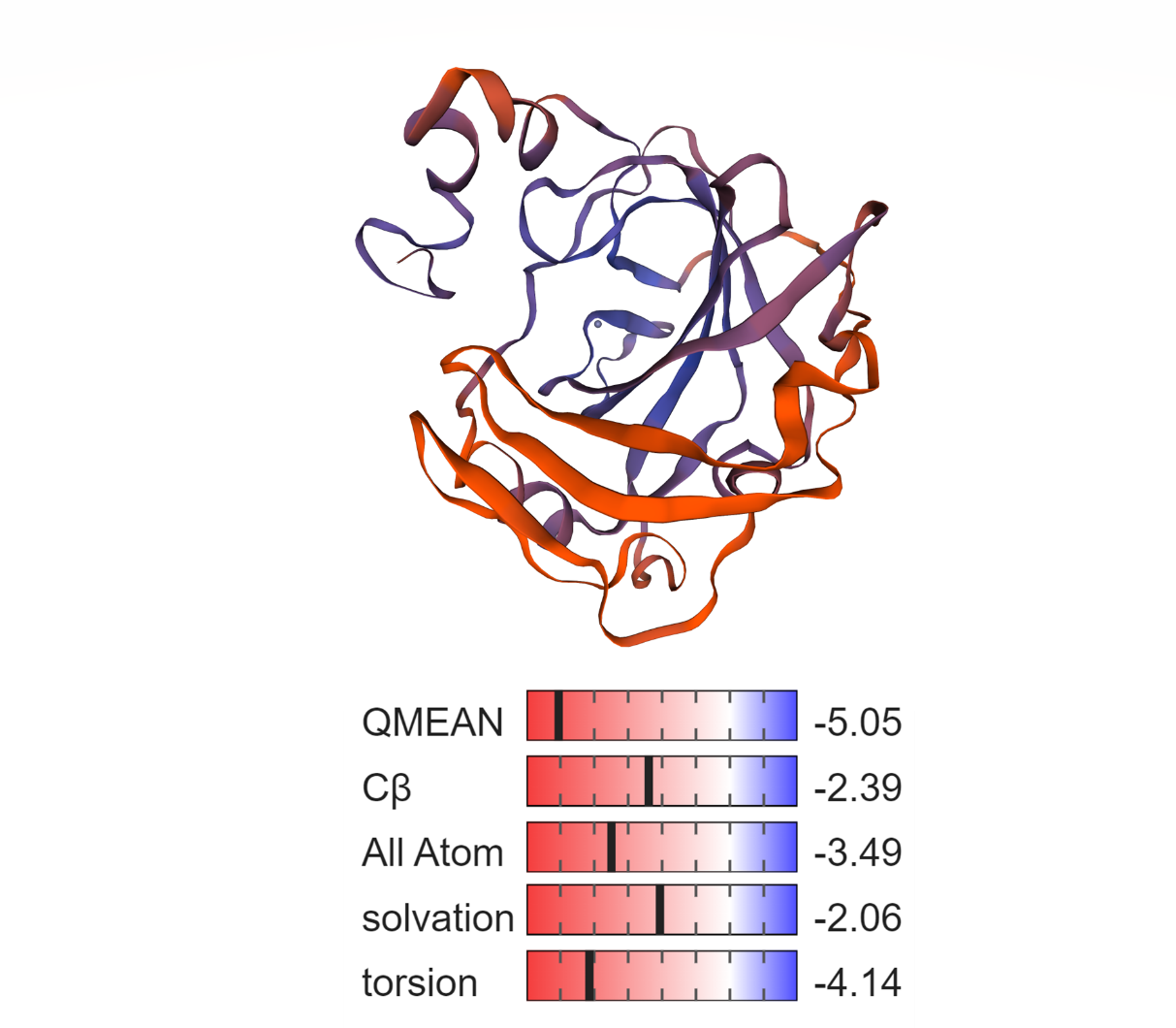

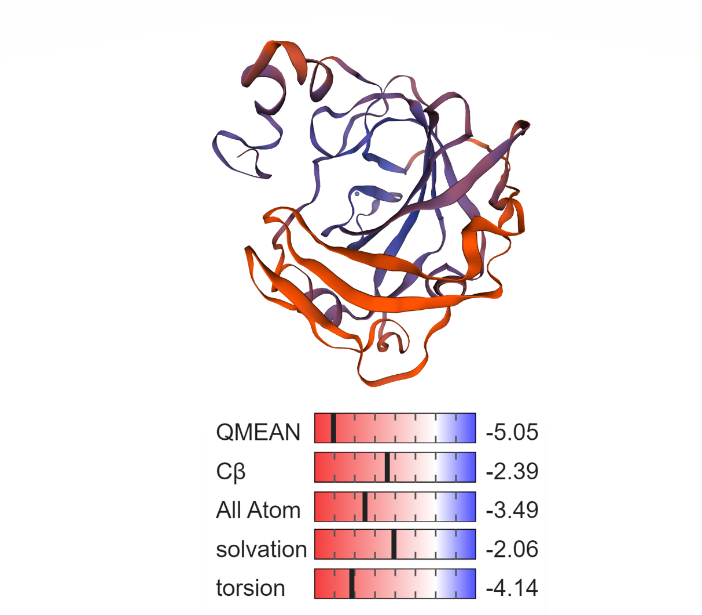
**

**Figure S4.** 3D structure of the homology model of EfCAα and related parameters calculated from SWISS-MODEL.


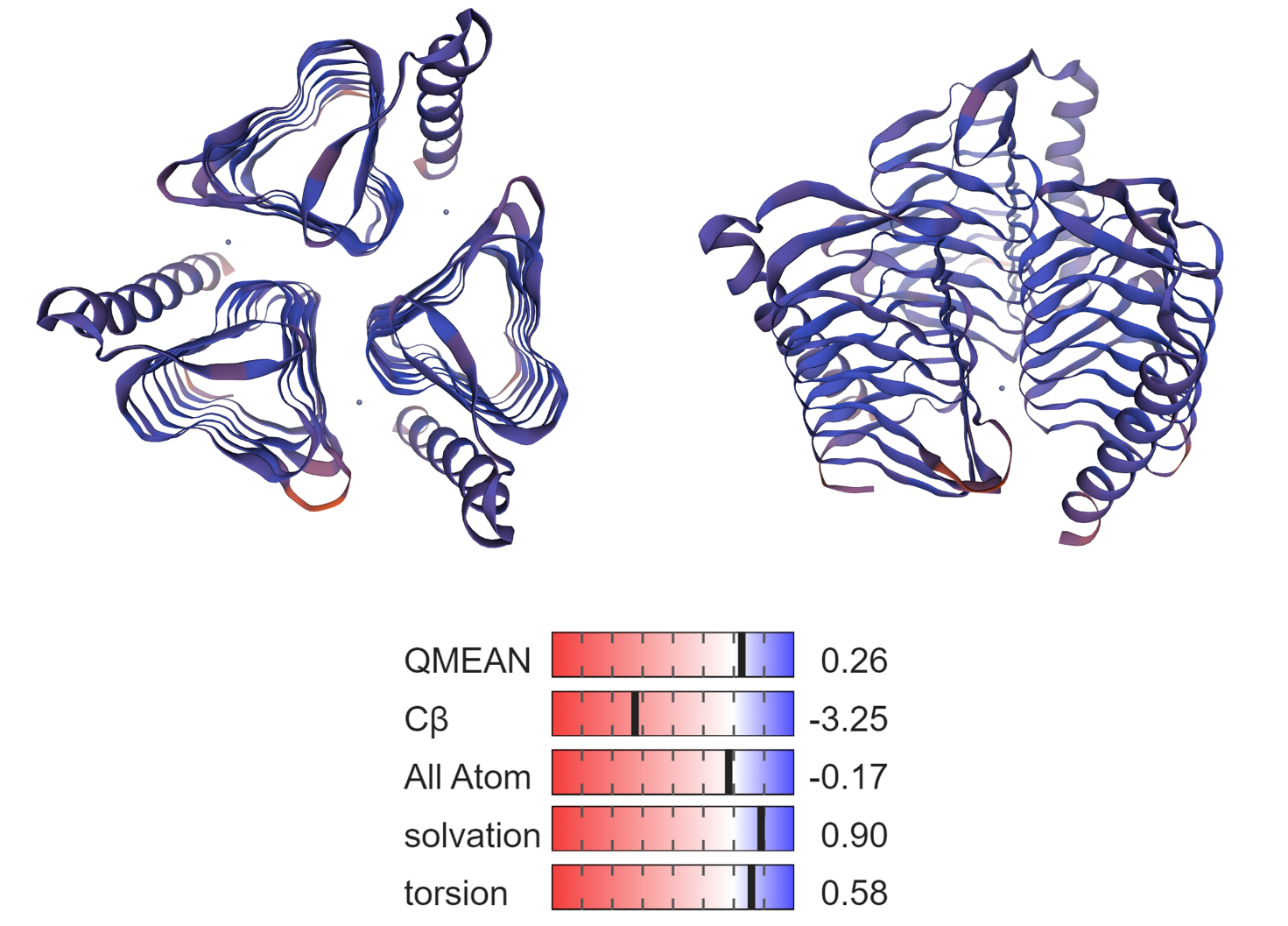

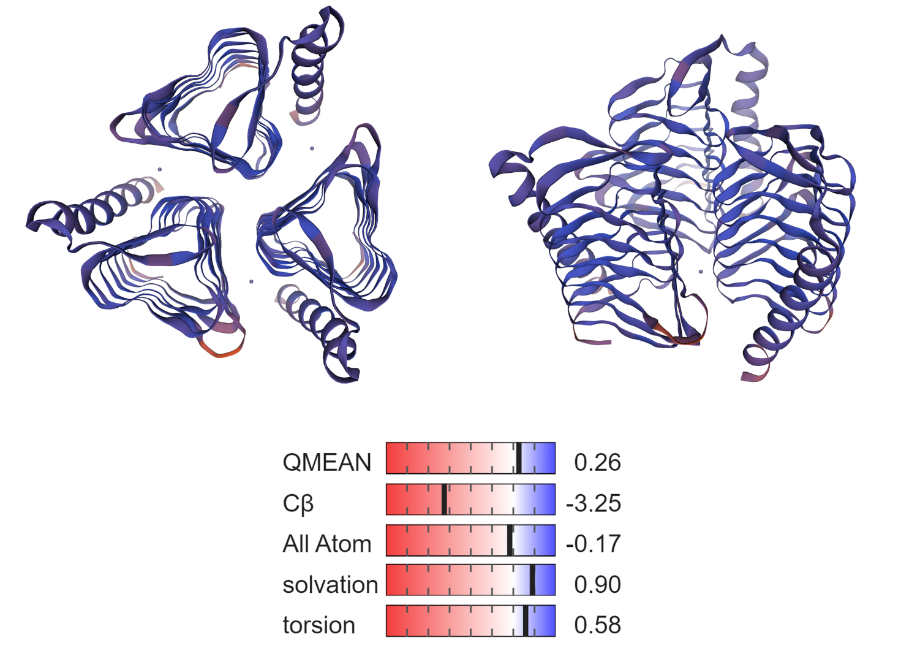


**Figure S5.** 3D structure of the homology model of EfCAγ and related parameters calculated from SWISS-MODEL.


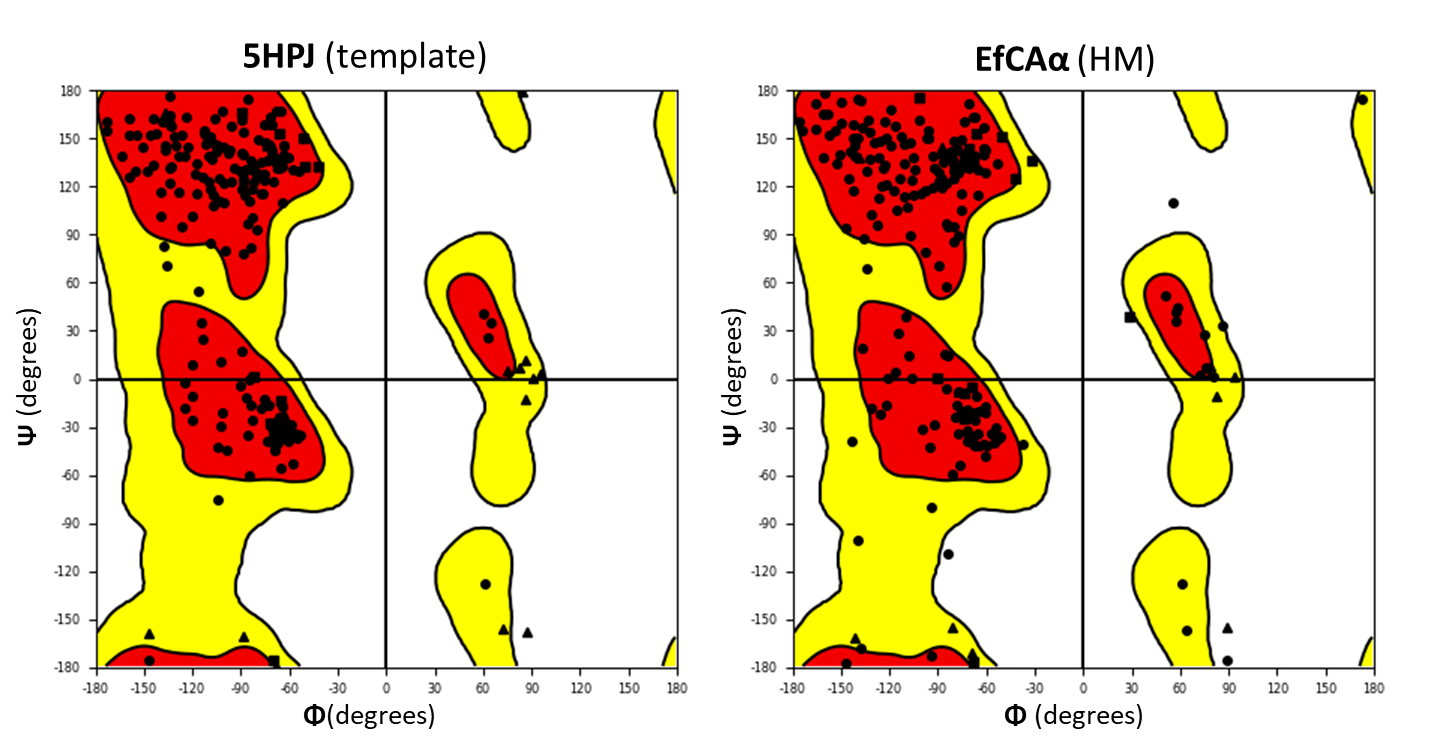


**Figure S6.** Ramachandran Plot of 5JHP (template) and EfCAα (HM).


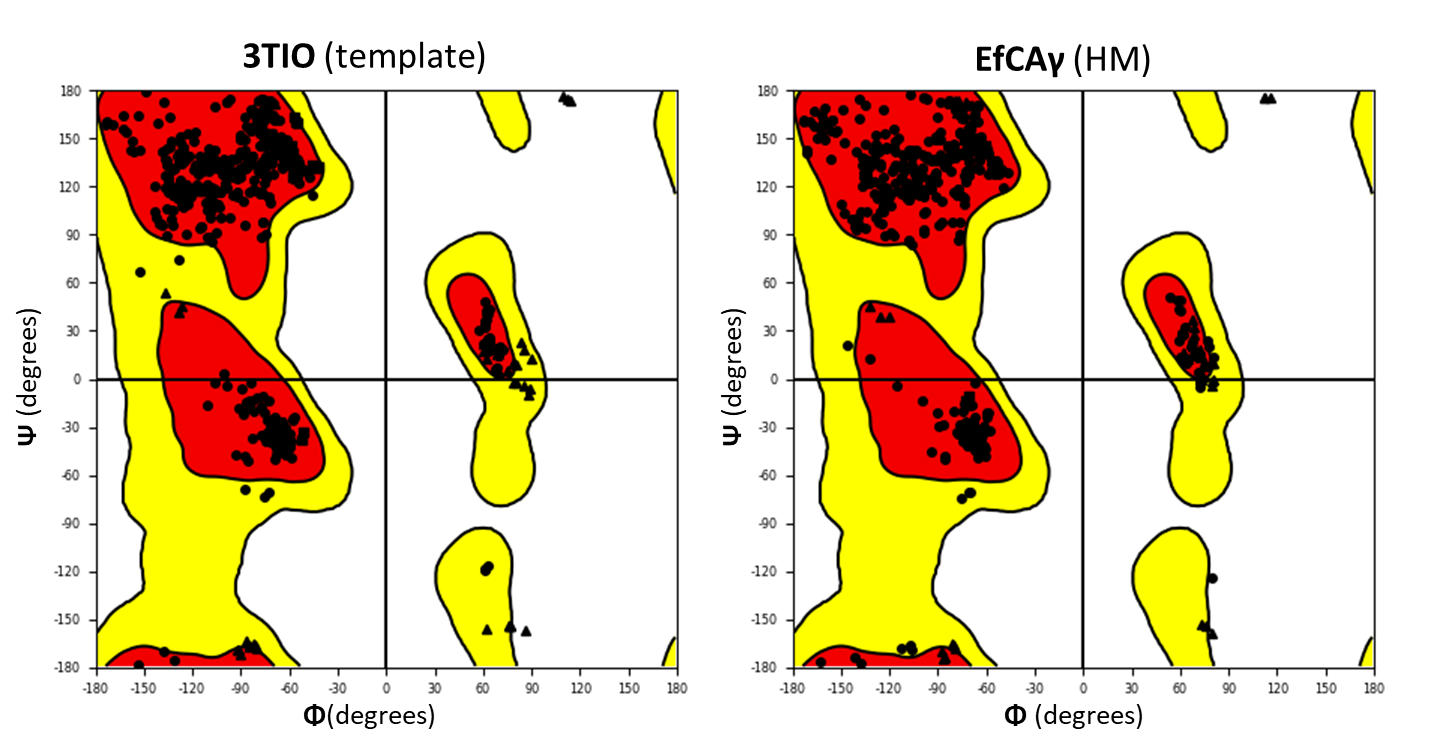


**Figure S7.** Ramachandran Plot of 3TIO (template) and EfCAγ (HM).

**Table S1.** Structural parameters of 5HPJ (template) and EfCAα (HM).

|  | **5HPJ** | **EfCAα** |
| --- | --- | --- |
| **MolProbity score** | 0.99 | 1.49 |
| **Clash score** | 0.59 | 2.00 |
| **Ramachandran Favoured** | 95.81 % | 91.20 % |
| **Ramachandran Outliers** | 0.00 % | 1.85 % |
| **Rotamer Outliers** | 0.00 % | 1.02 % |
| **C-Beta Deviations** | 0 | 10 |
| **Bad Bonds** | 5/1768 | 0/1881 |
| **Bad Angles** | 26/2415 | 32/2558 |
| **QMEAN** | 0.06 | -5.05 |
| **Cβ** | -0.33 | -2.39 |
| **All Atom** | -0.61 | -3.49 |
| **solvation** | -0.70 | -2.06 |
| **torsion** | 0.42 | -4.14 |

**Table S2.** Structural parameters of 3TIO (template) and EfCAγ (HM).

|  | **3TIO** | **EfCAγ** |
| --- | --- | --- |
| **MolProbity score** | 1.18 | 1.46 |
| **Clash score** | 1.47 | 3.67 |
| **Ramachandran Favoured** | 97.89 % | 95.69 % |
| **Ramachandran Outliers** | 0.00 % | 0.86 % |
| **Rotamer Outliers** | 2.22 % | 0.27 % |
| **C-Beta Deviations** | 0 | 4 |
| **Bad Bonds** | 2/4159 | 2/3588 |
| **Bad Angles** | 60/5649 | 44/4865 |
| **Cis Prolines** | 3/24 | 3/15 |
| **QMEAN** | 0.98 | 0.26 |
| **Cβ** | -2.39 | -3.25 |
| **All Atom** | -0.59 | -0.17 |
| **solvation** | 0.33 | 0.90 |
| **torsion** | 1.26 | 0.58 |

**
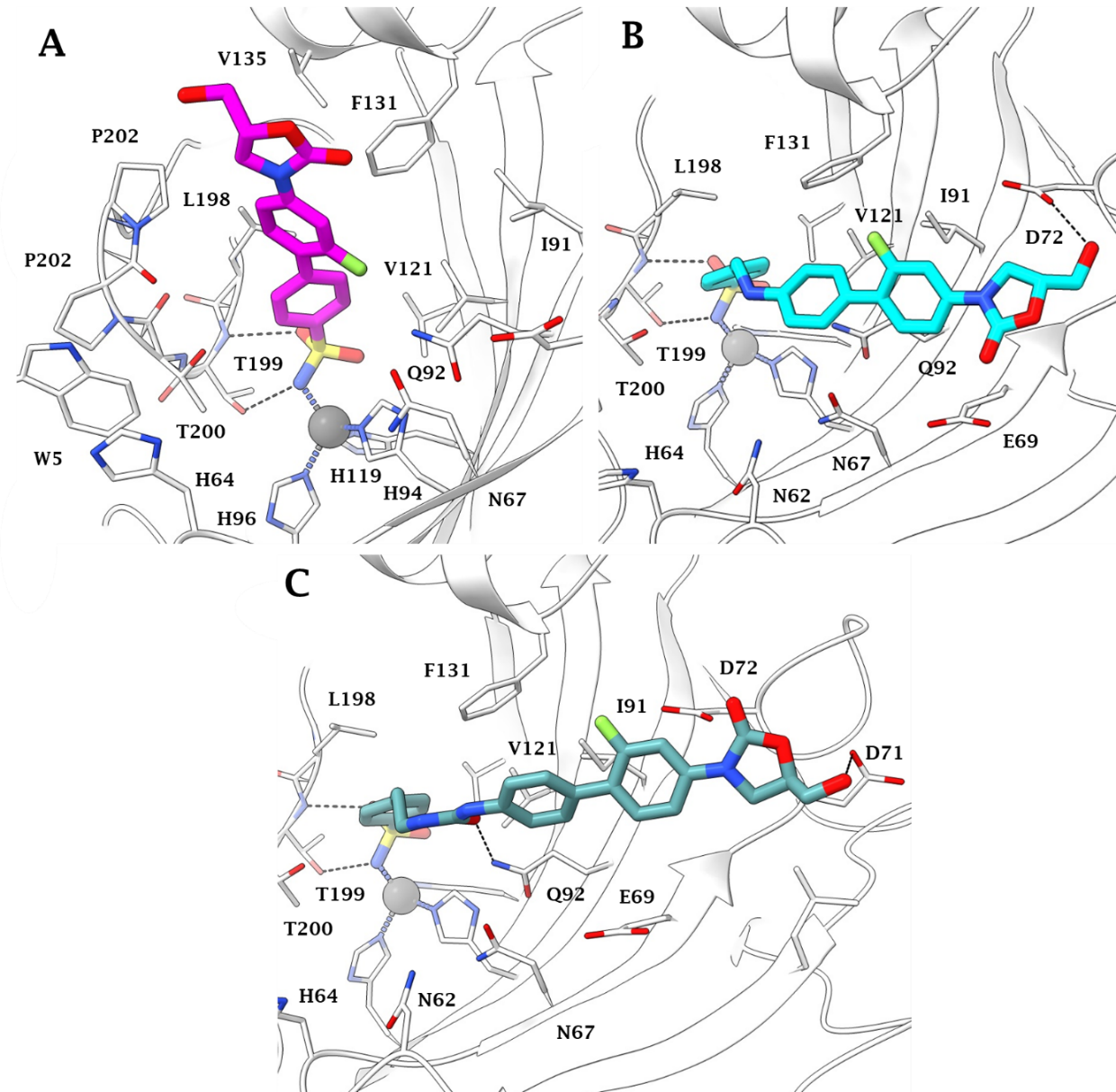
**

**Figure S8.** Binding mode of A) **8** (magenta), B) **17** (cyan) and C) **21** (green) in the hCA II active site predicted by docking/MM-GBSA. H-bonds are depicted as black dashed lines.

**Synthetic Procedures and Characterizations of Intermediates**

**2-Azido-*N*-(5-sulfamoyl-1,3,4-thiadiazol-2-yl)acetamide (E).** An ice-cold solution of 2-chloro-*N*-(5-sulfamoyl-1,3,4-thiadiazol-2-yl)acetamide (**S17**) (200 mg, 1 eq.) in anhydrous DMF under inert atmosphere was treated with sodium azide (2 eq.) and stirred at room temperature o.n. The reaction was quenched with slush, the readily formed precipitate was collected by filtration and used without further purification. White solid; yield 65%; silica gel TLC R*_f_* (MeOH/DCM 5% *v*/*v*) 0.26; δ*_H_* (400 MHz, DMSO-*d_6_*): 13.30 (s, 1H, exchange with D_2_O, CON*H*), 8.40 (s, 2H, exchange with D_2_O, SO_2_N*H*_2_), 4.35 (s, 1H, C*H_2_*); δ*_c_* (400 MHz, DMSO-*d_6_*): 169.48, 164.37, 161.18, 65.22.

**Phenyl (5-sulfamoyl-1,3,4-thiadiazol-2-yl)carbamate (J).** To an ice-cold solution of 5-amino-1,3,4-thiadiazole-2-sulfonamide (**S16**) (200 mg, 1 eq.) in anhydrous THF under inert atmosphere was dropwise added phenyl chloroformate (2 eq.). The white suspension was stirred in ice bath for 15’ then refluxed for 1h. The solvent was evaporated, and the residue was treated with slush. The readily formed precipitate was collected by filtration, washed with Et_2_O and used without further purification. White solid; yield 85%; silica gel TLC R*_f_* (MeOH/DCM 5% *v*/*v*) 0.38; δ*_H_* (400 MHz, DMSO-*d_6_*): 13.32 (s, 1H, exchange with D_2_O, CON*H*), 8.41 (s, 2H, exchange with D_2_O, SO_2_N*H*_2_), 7.51 (t, 2H, *J*=7.7 Hz, 2xAr-*H*), 7.35 (m, 3H, 3xAr-*H*); δ*_c_* (400 MHz, DMSO-*d_6_*): 164.80, 163.86, 152.83, 150.04, 129.71, 126.44, 121.68.

^1^H, ^13^C and ^19^F spectra of derivatives **8**-**26**.


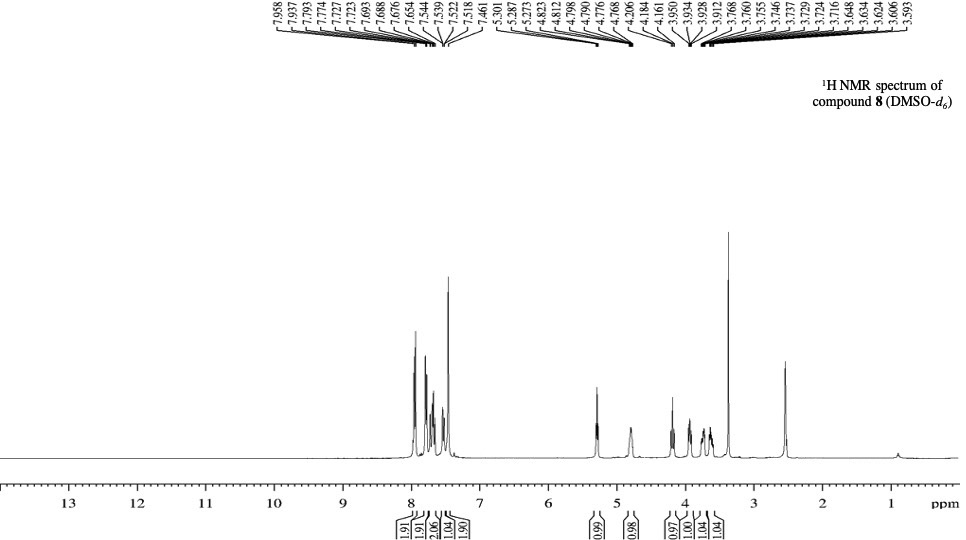


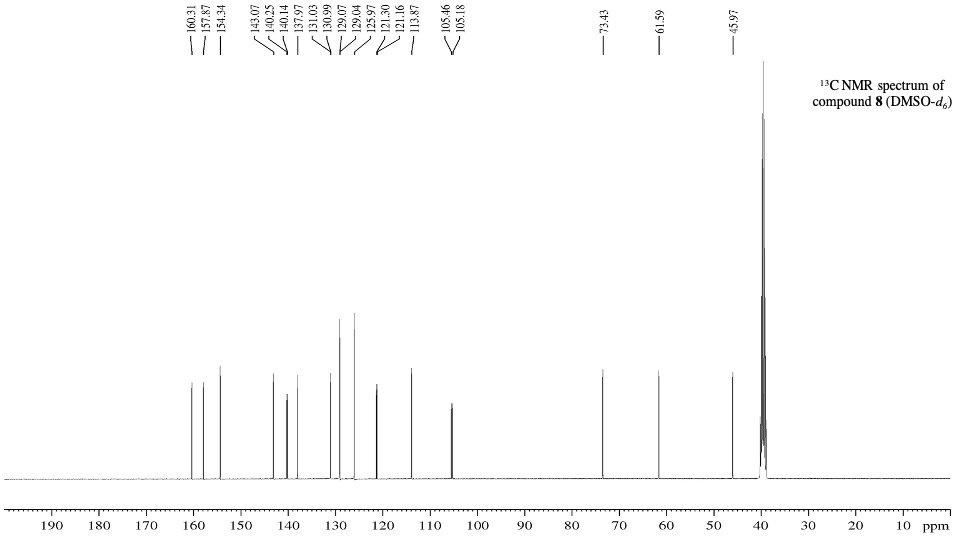


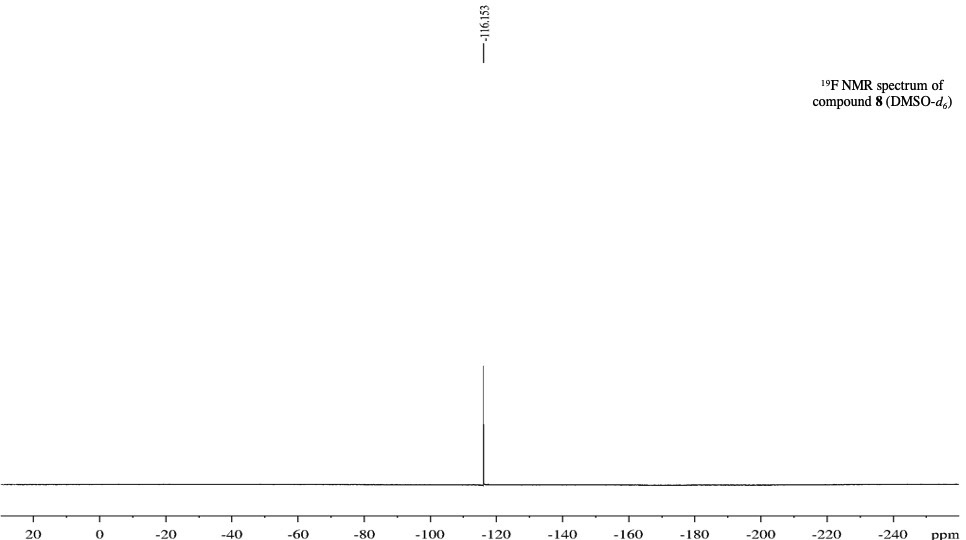


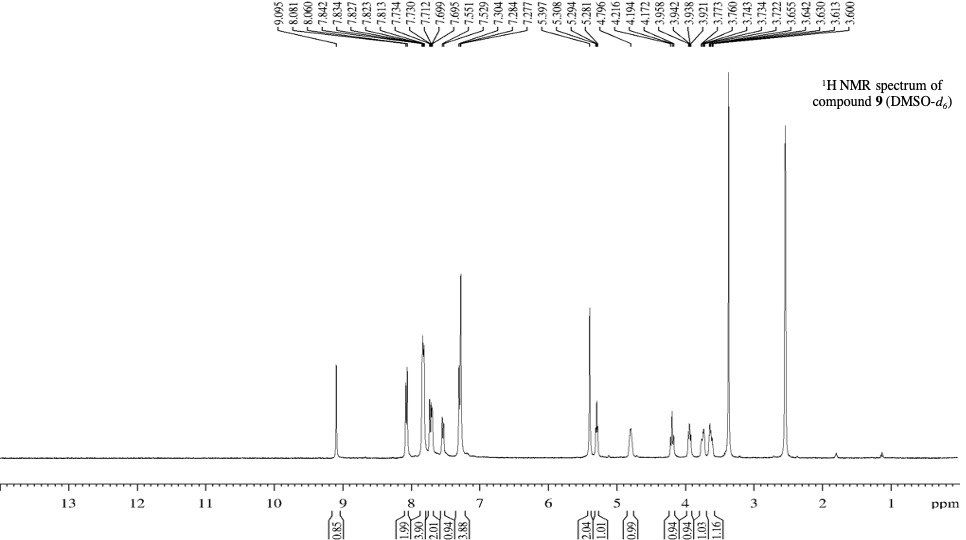


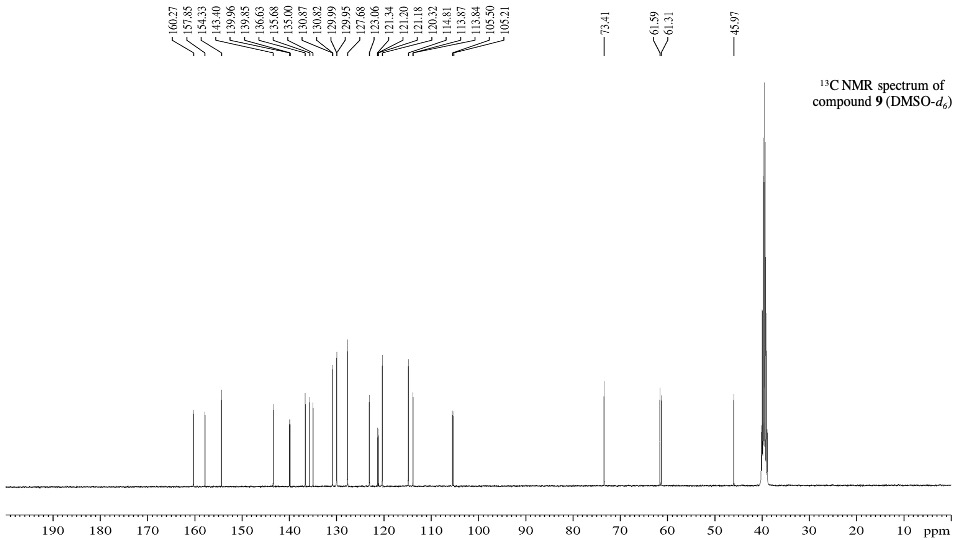


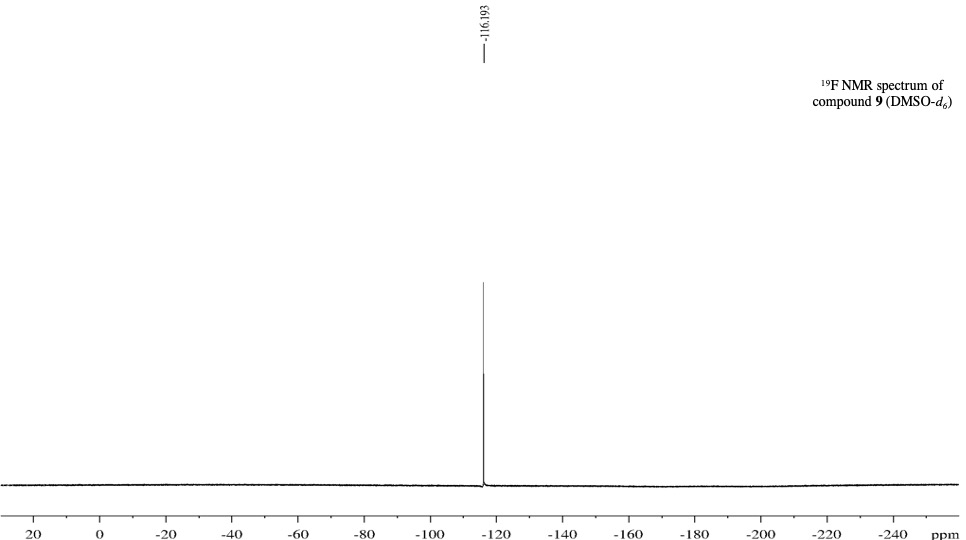


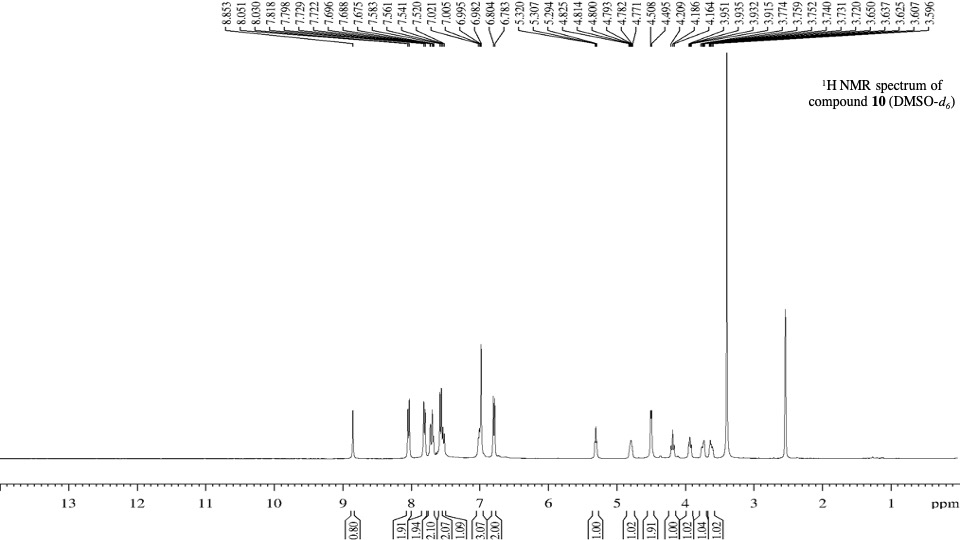


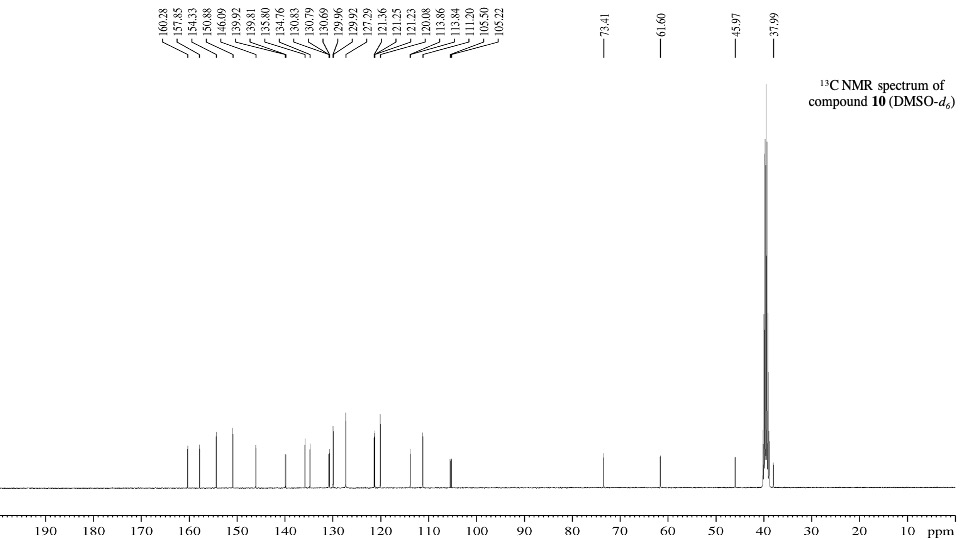


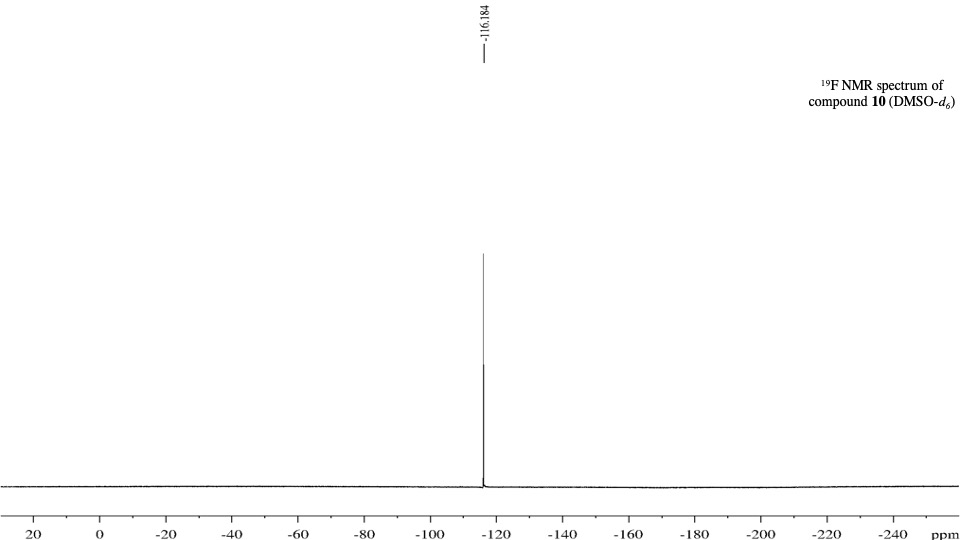


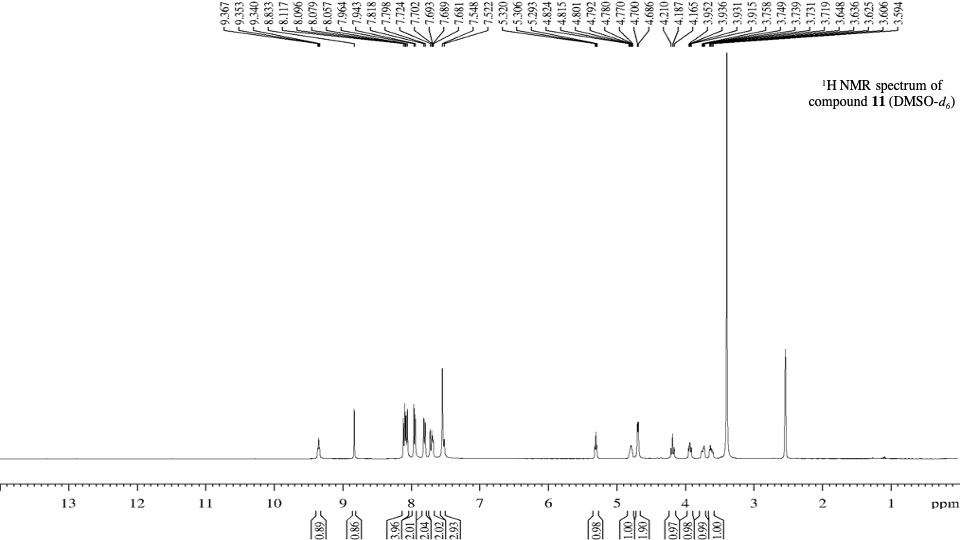


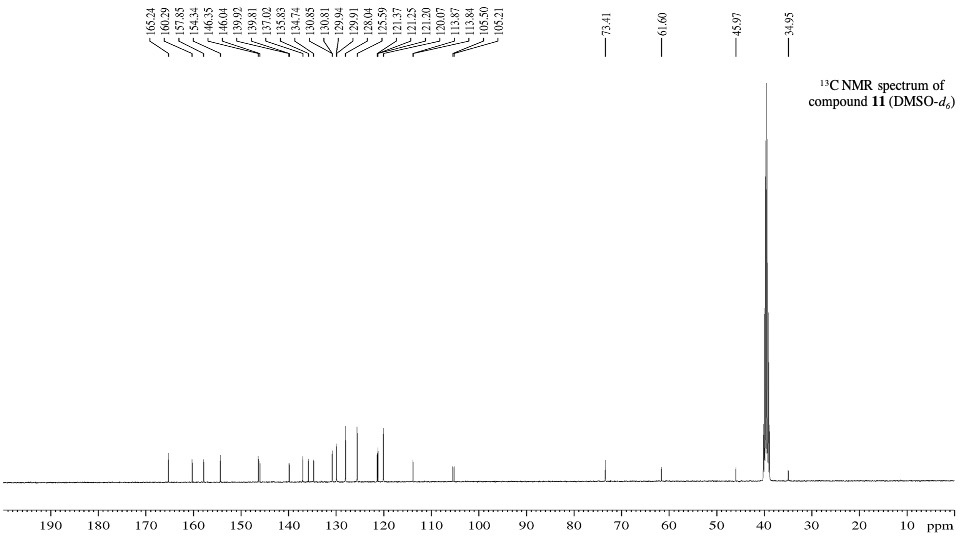


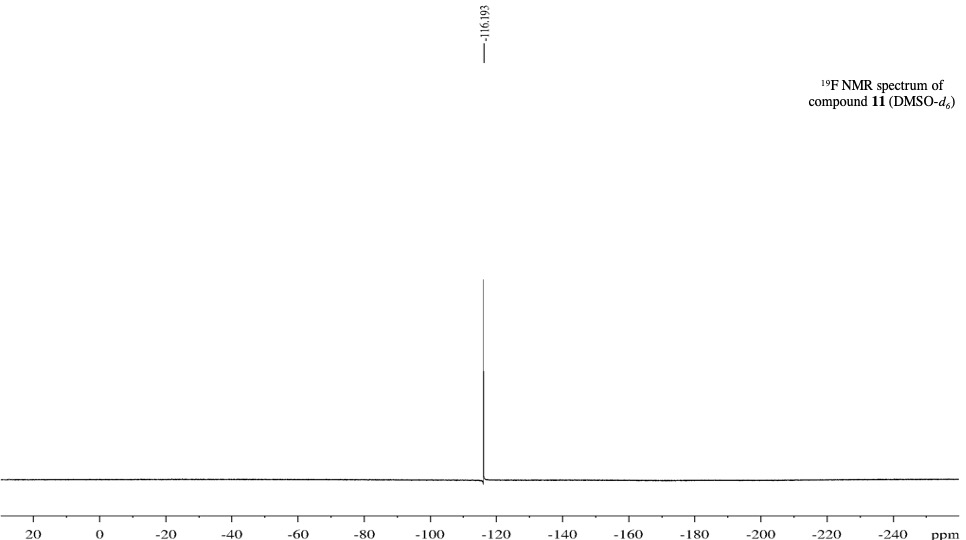


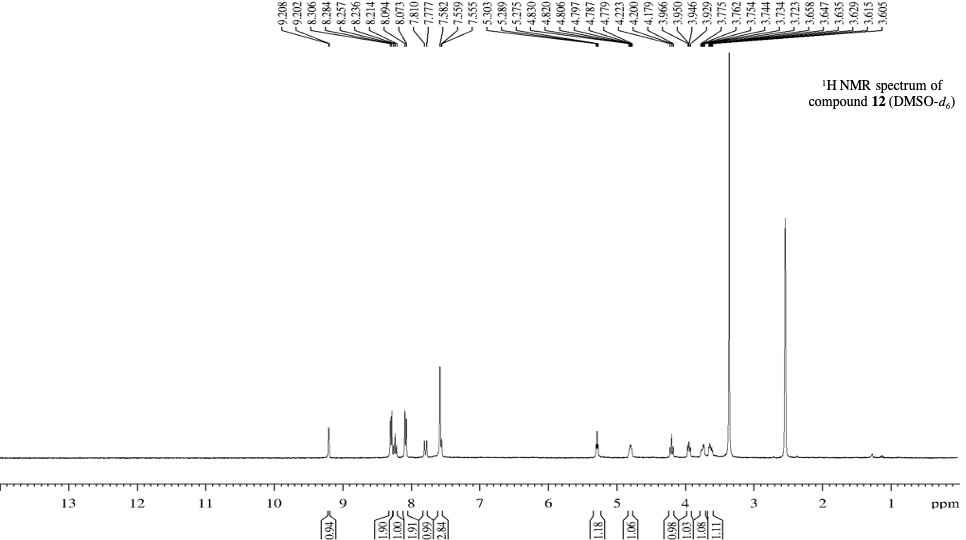


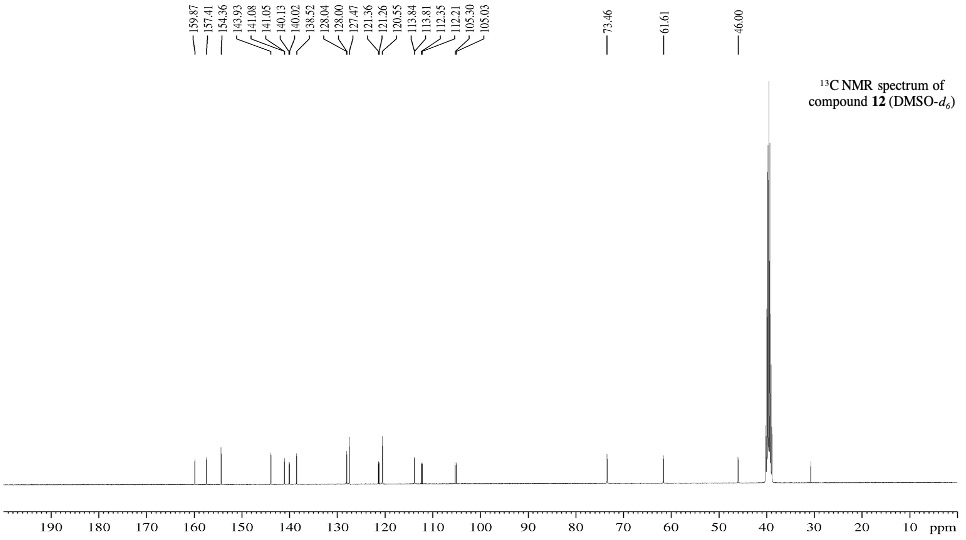


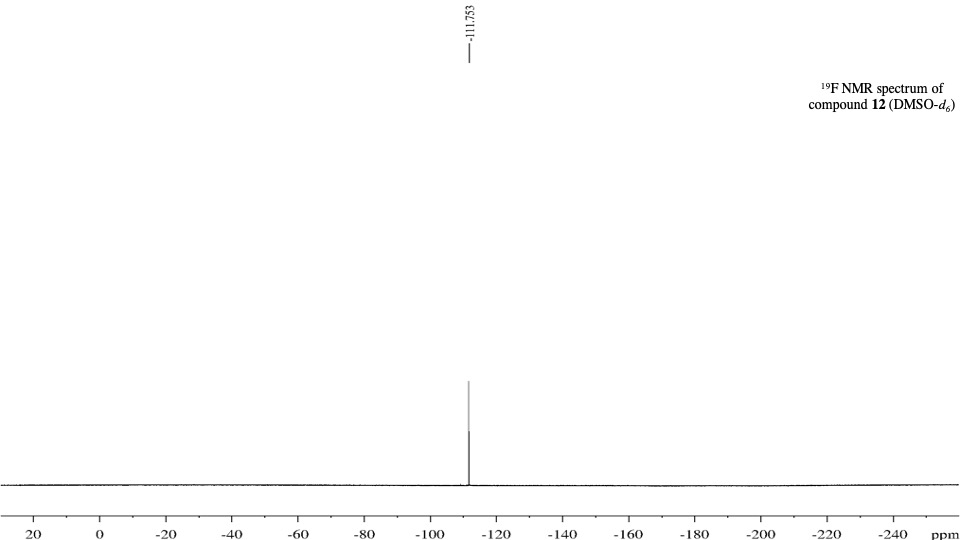


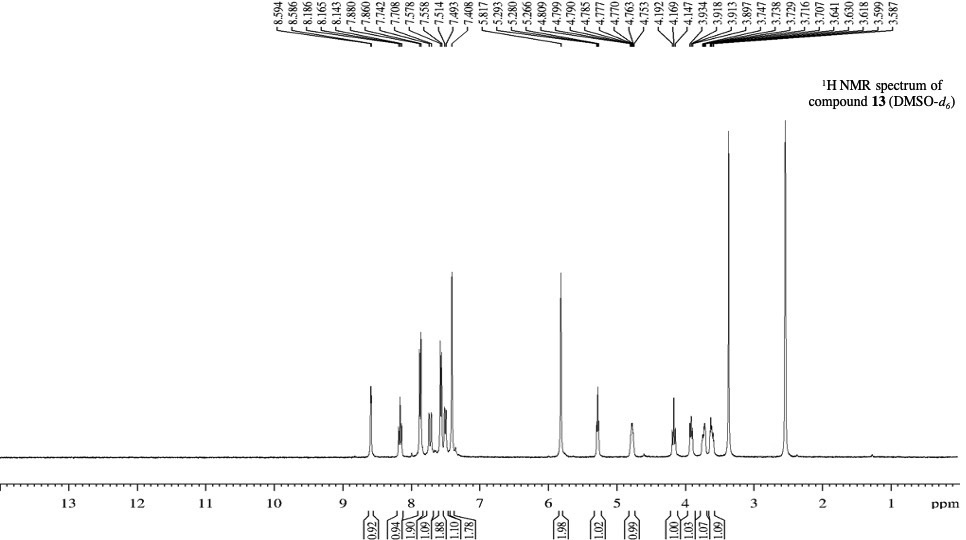


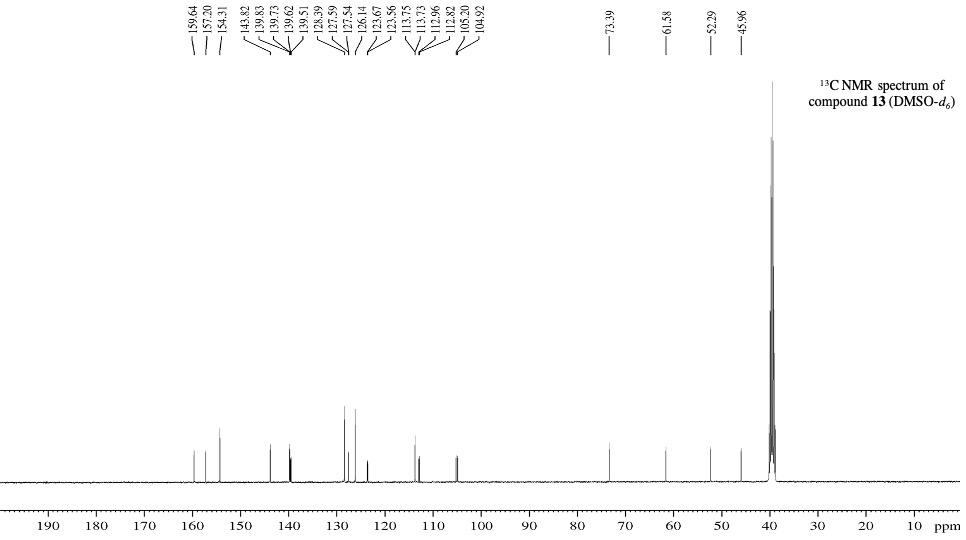


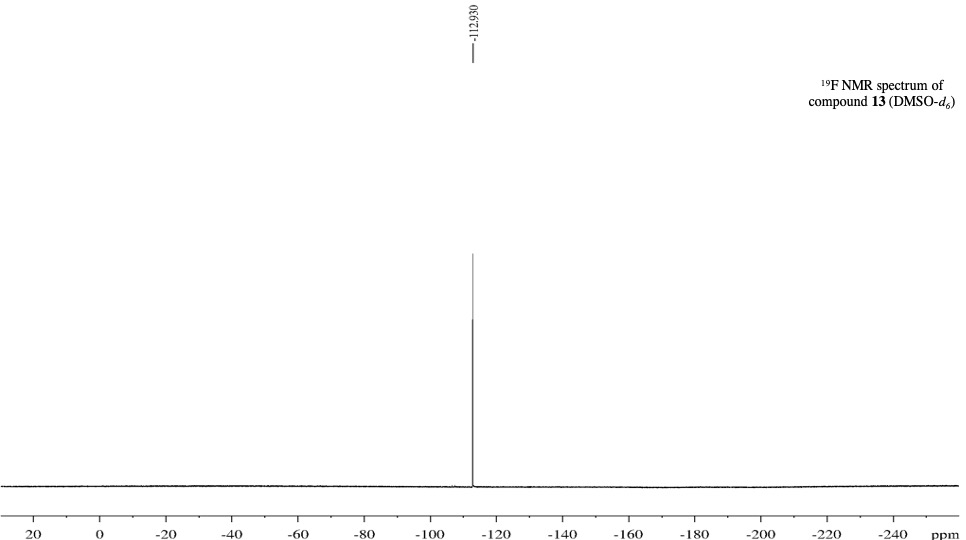


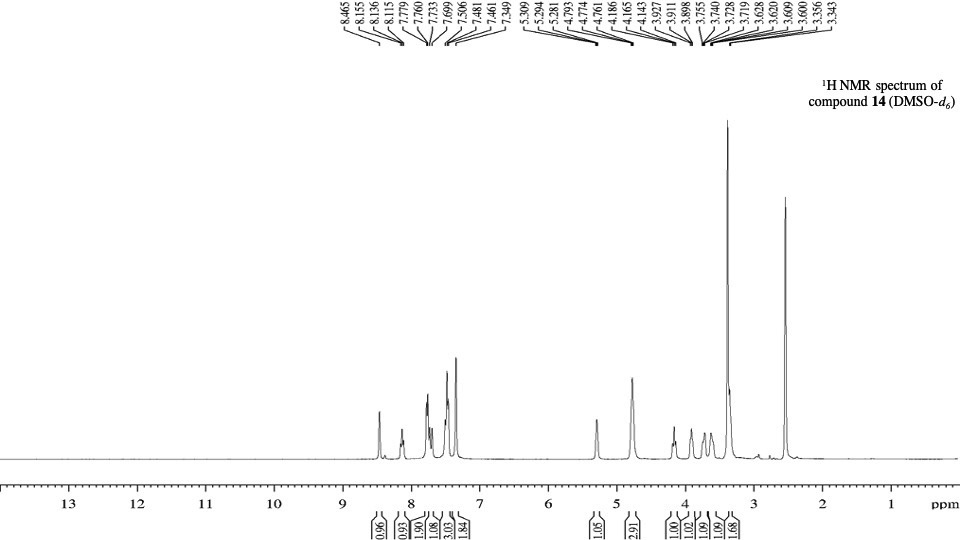


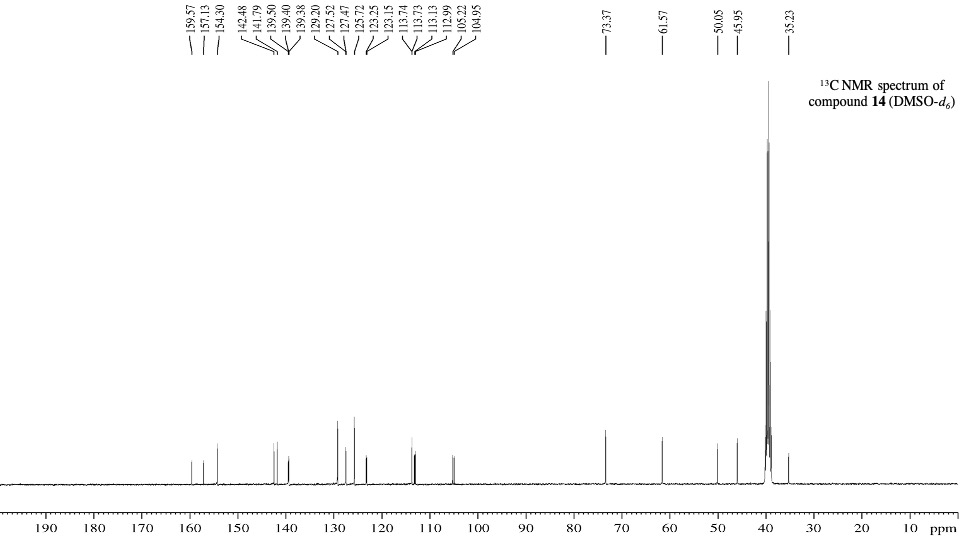


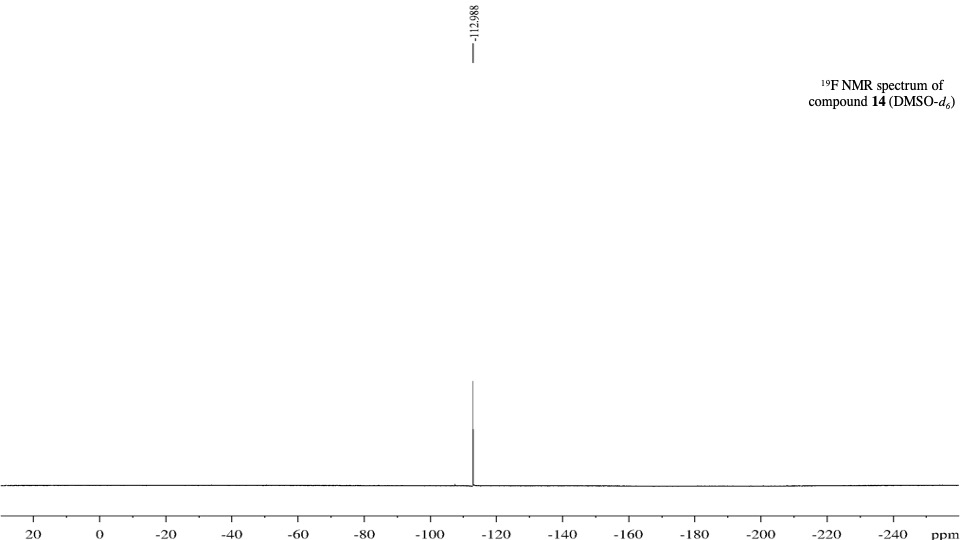


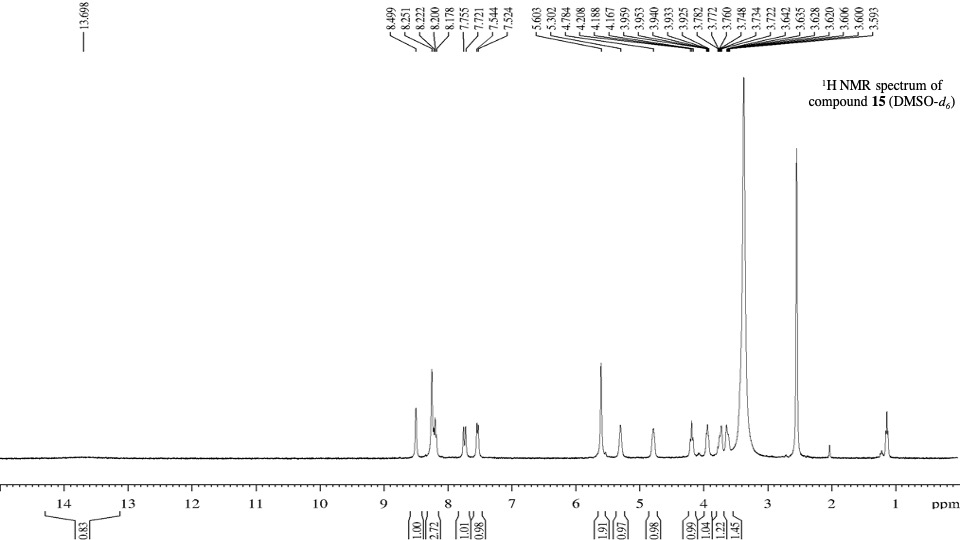


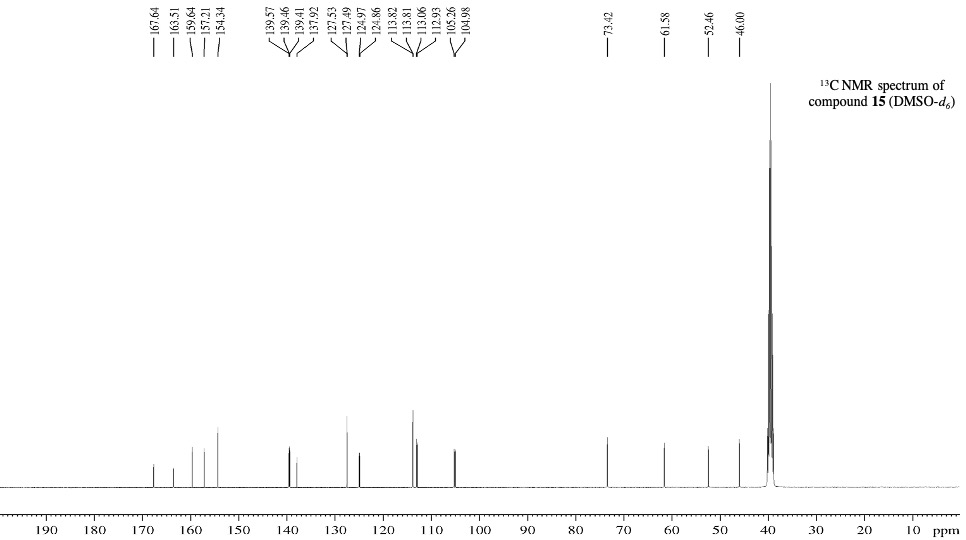


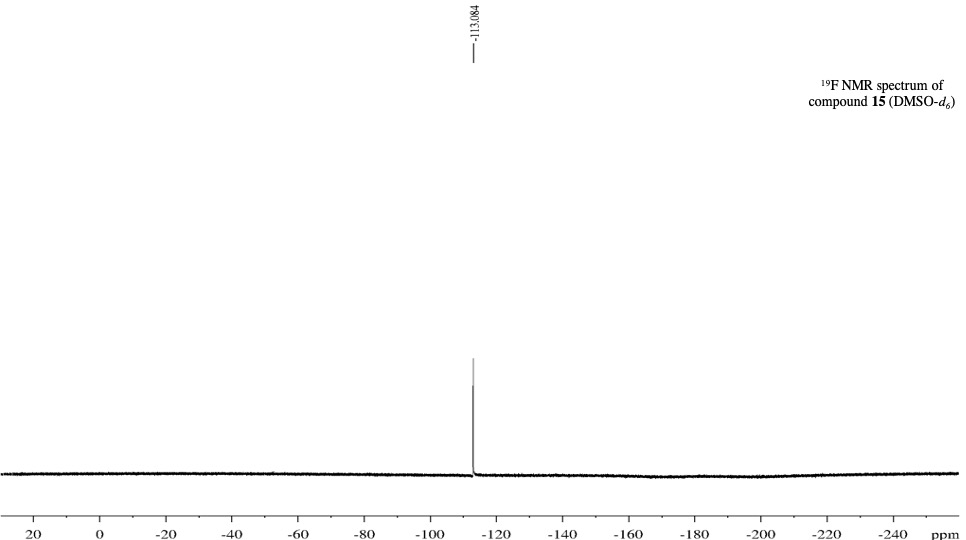


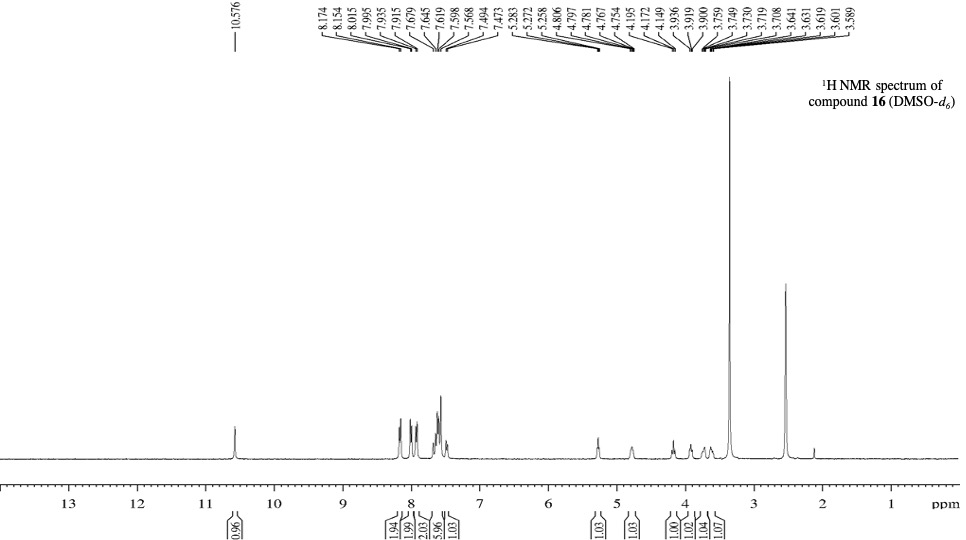


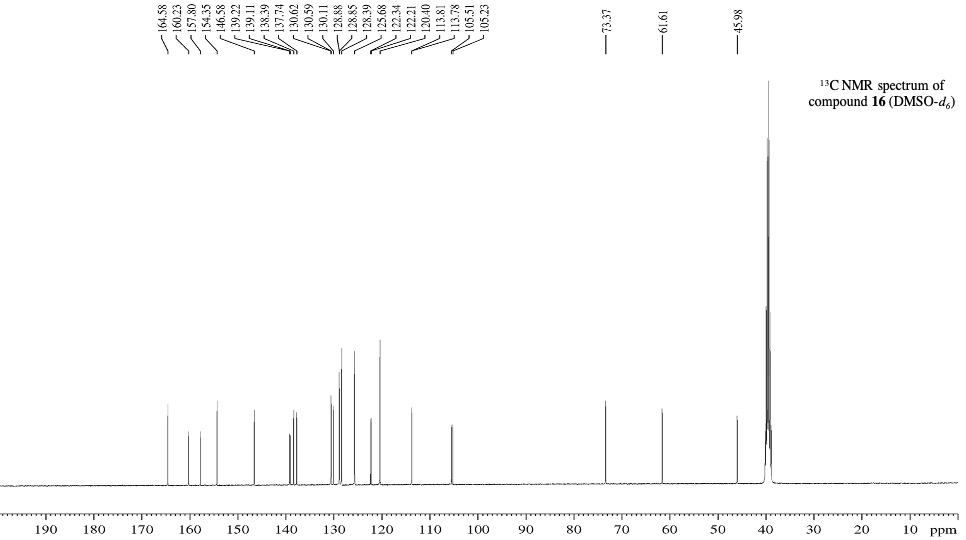


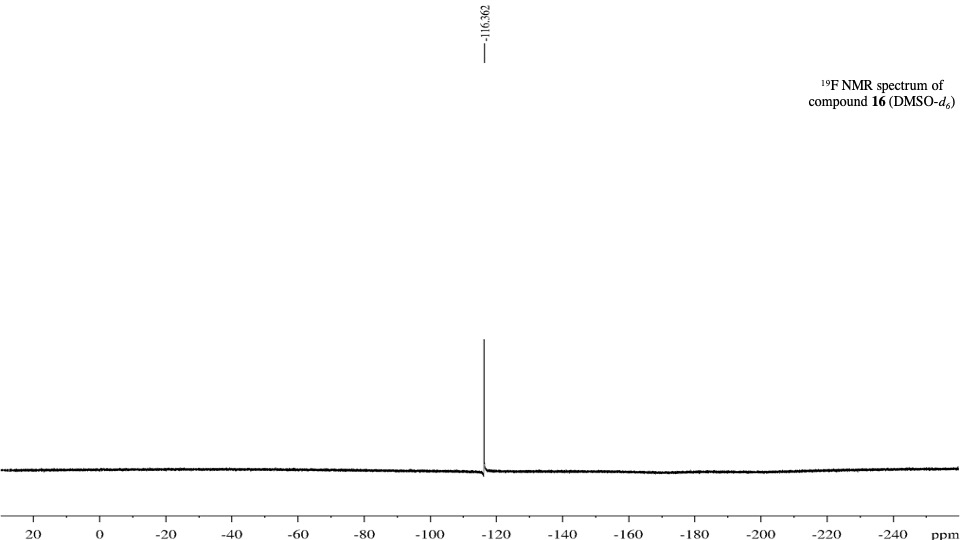


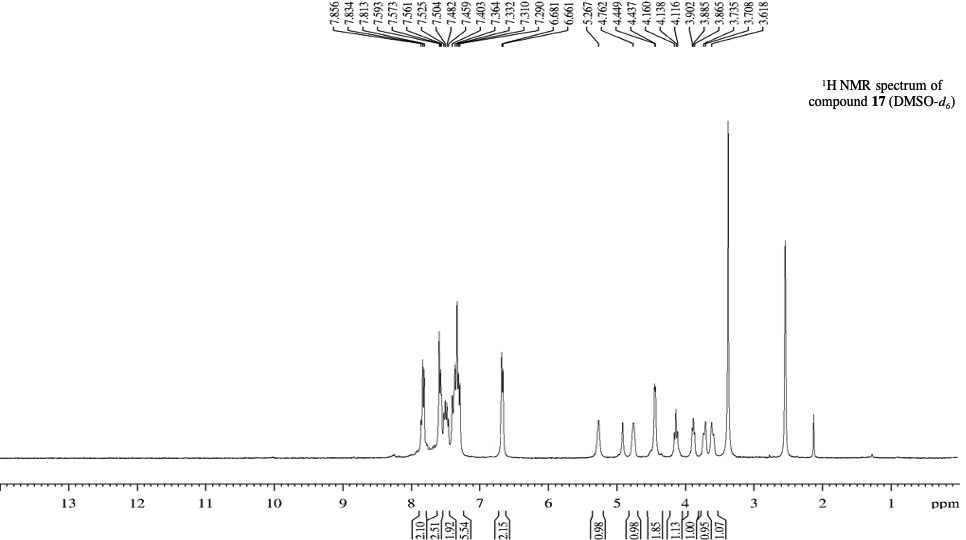


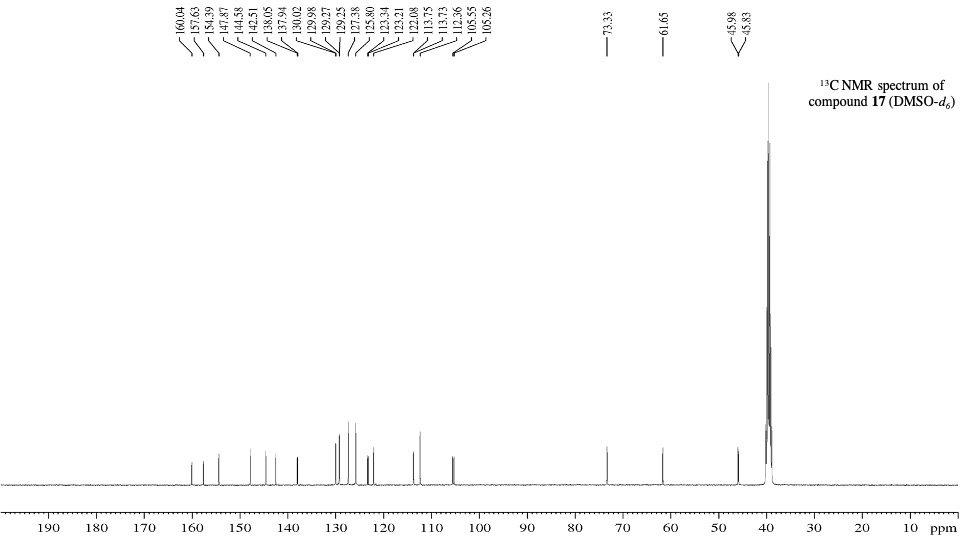


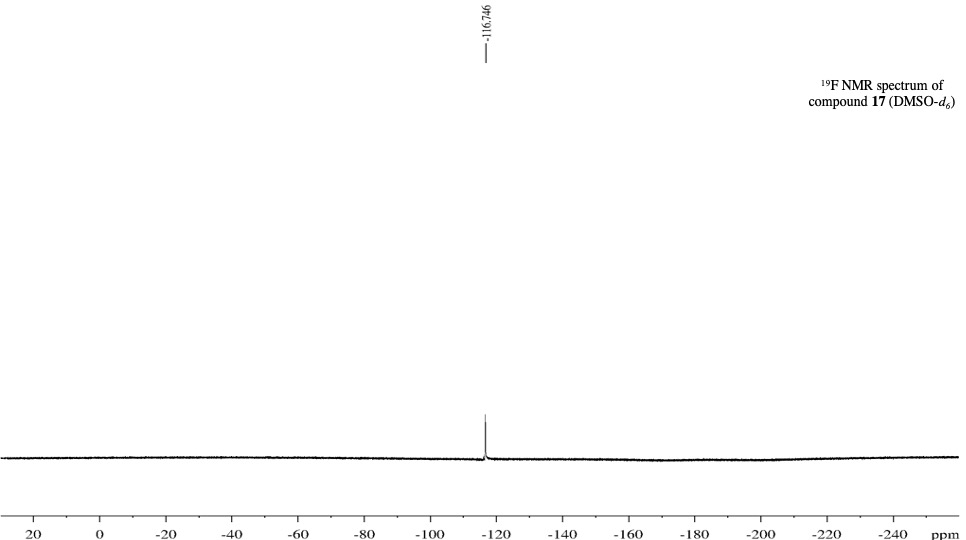


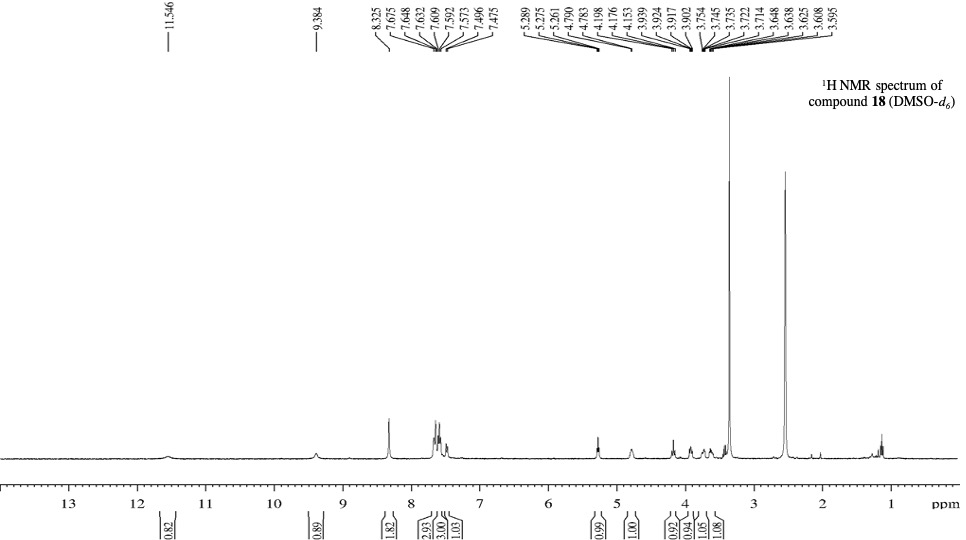


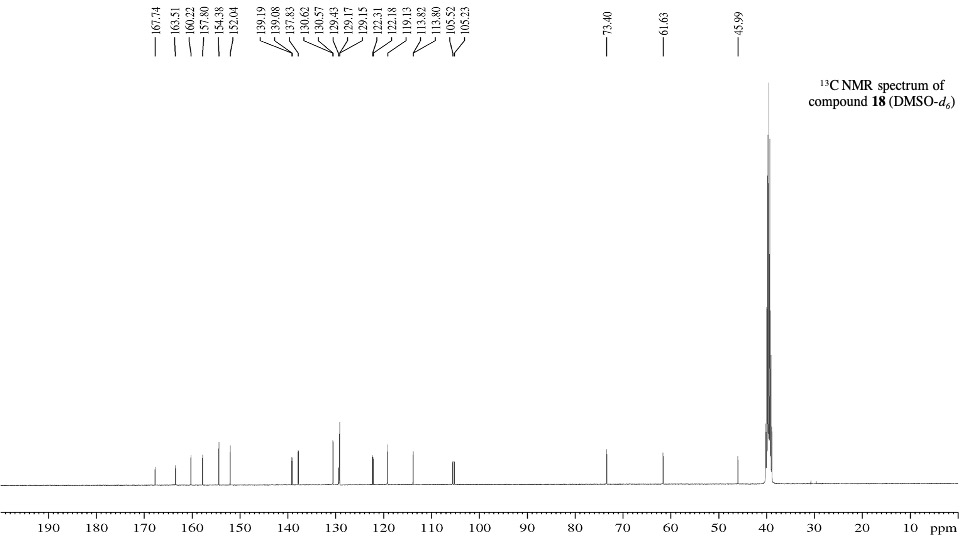


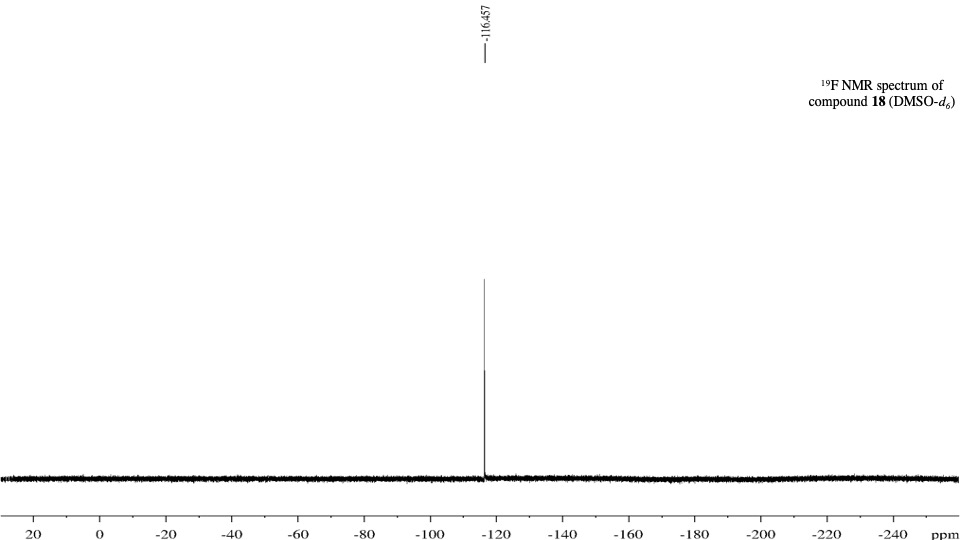


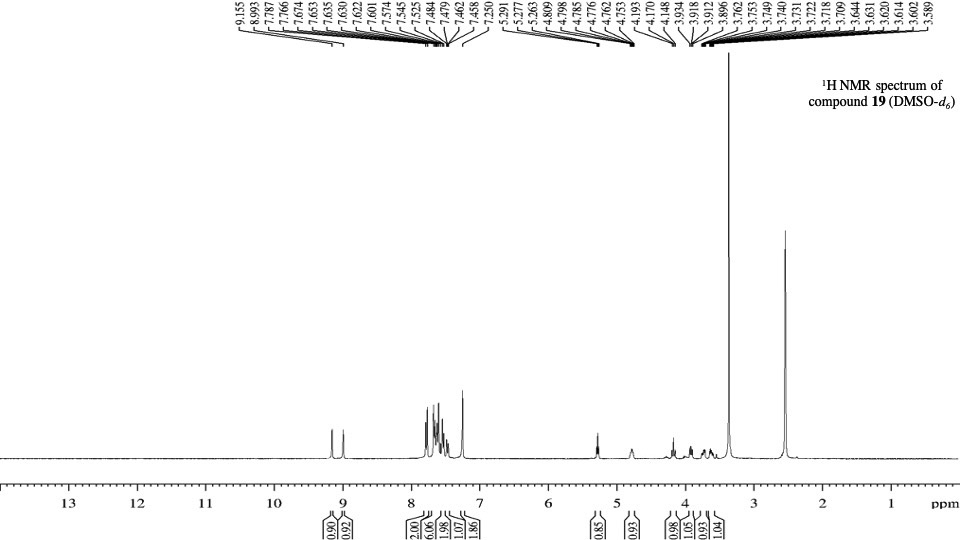


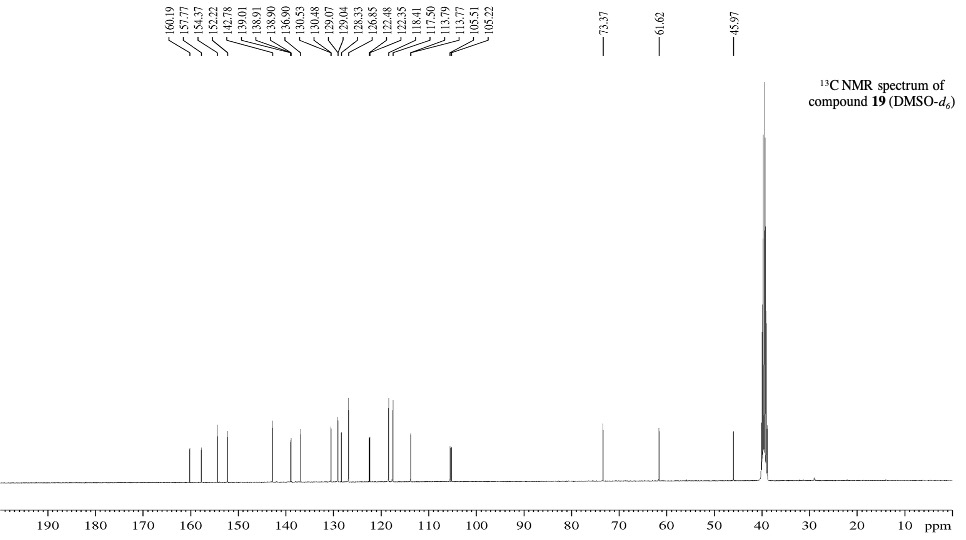


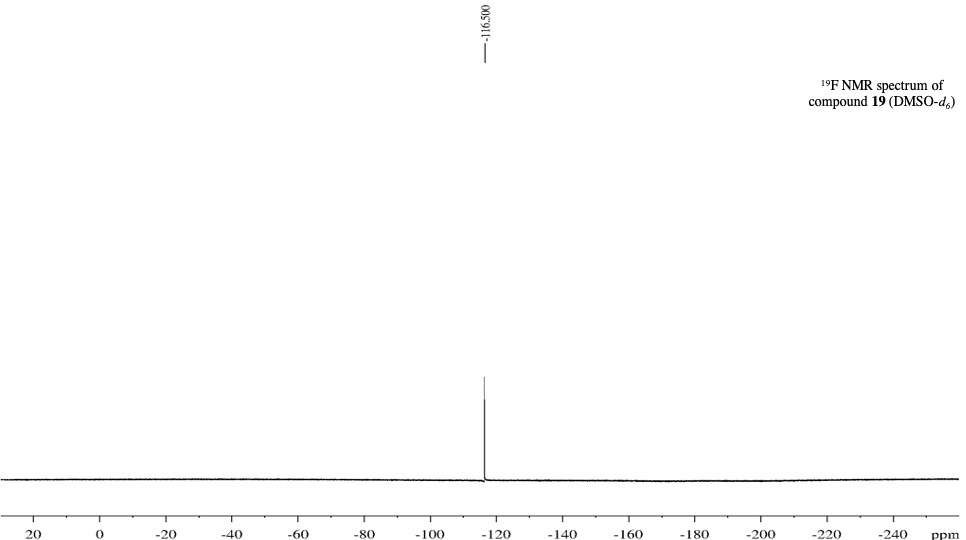


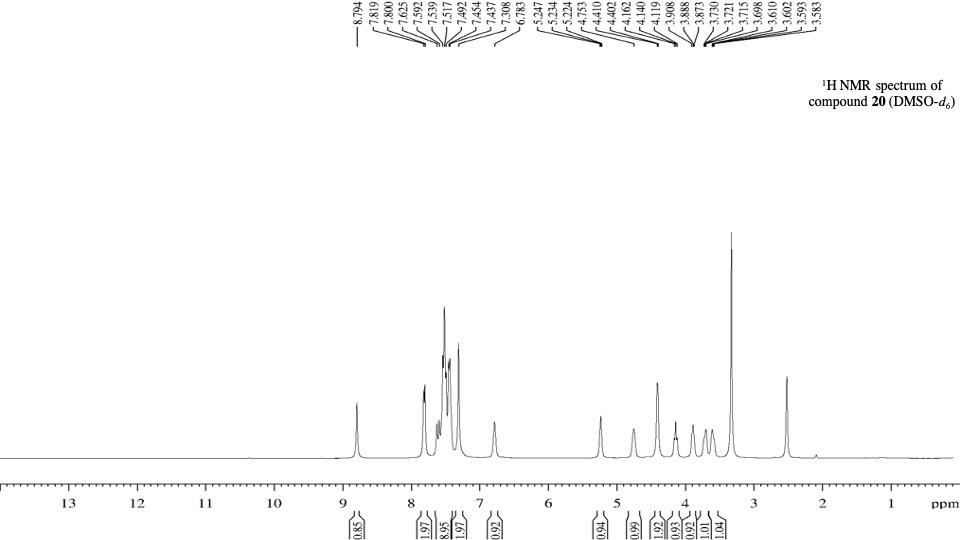


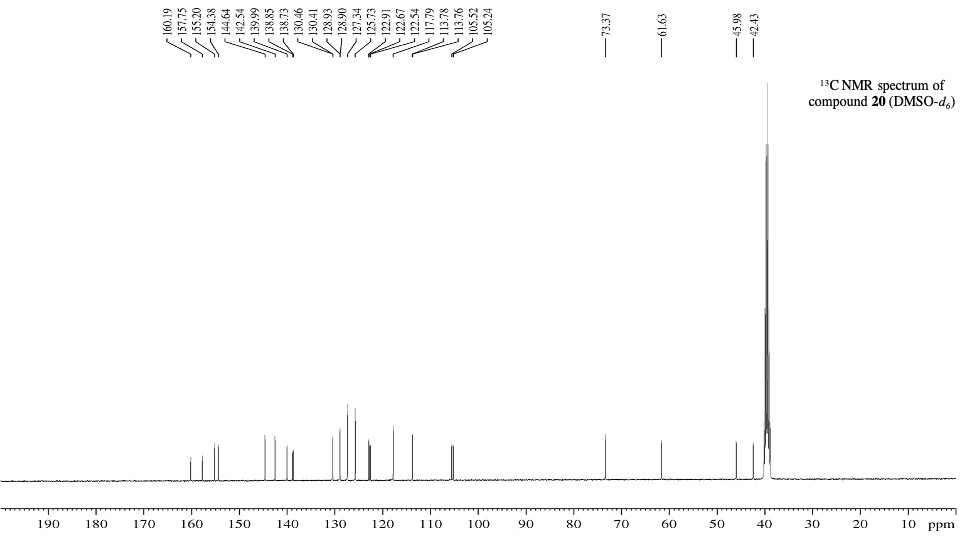


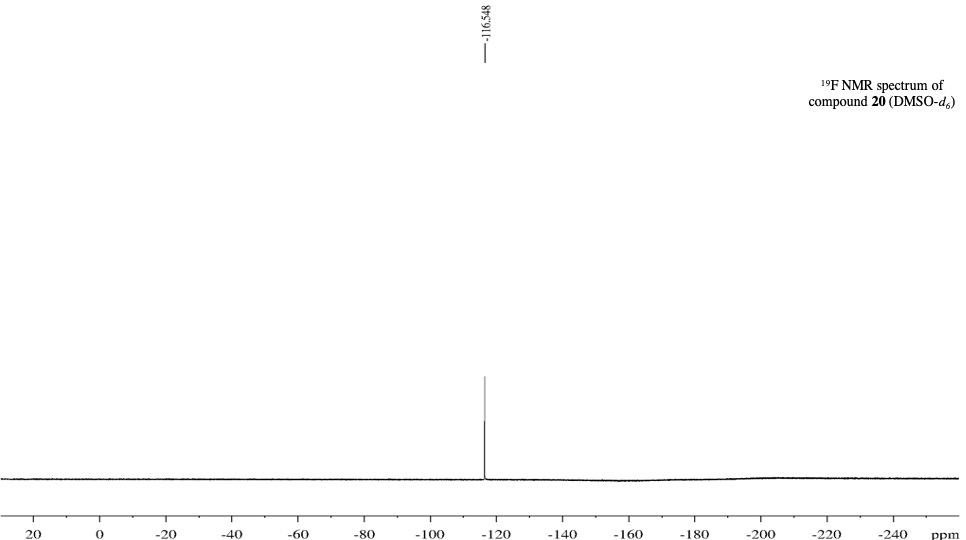


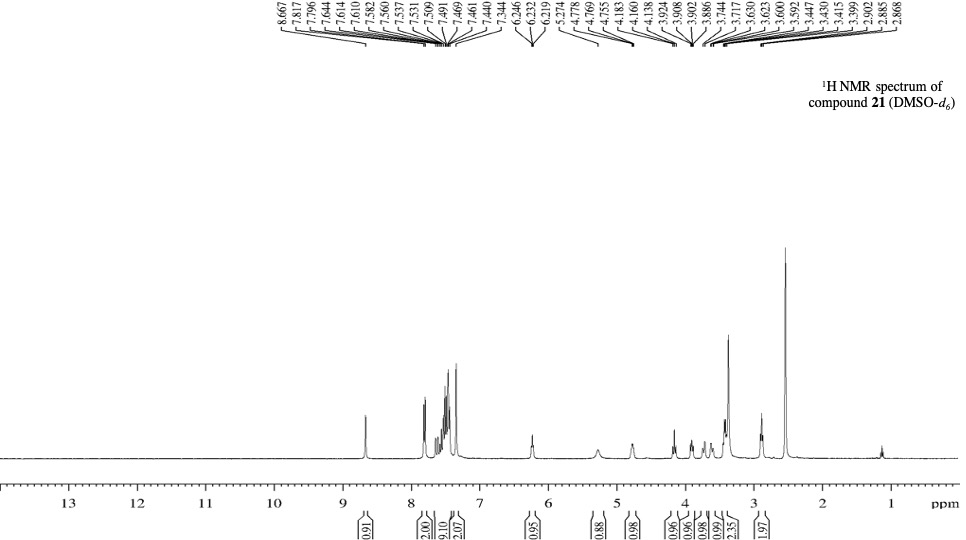


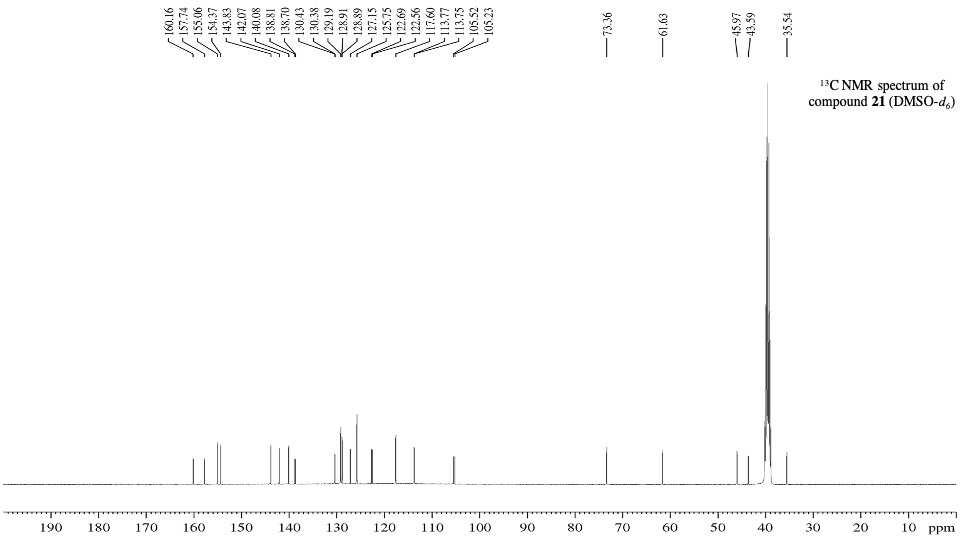


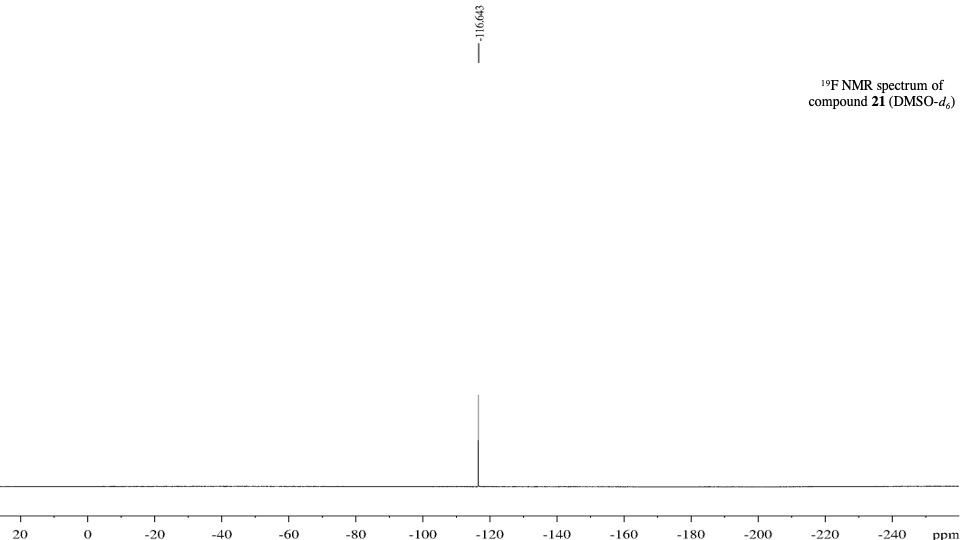


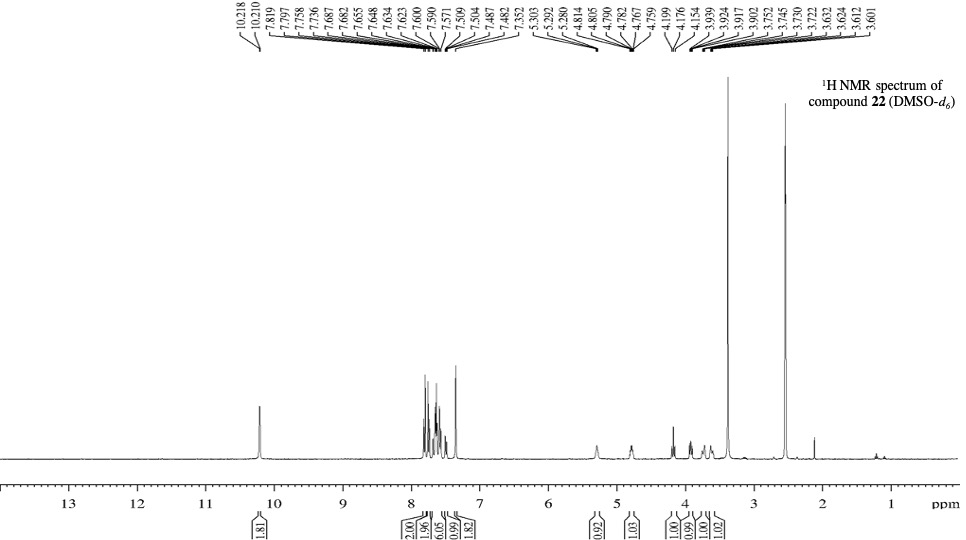


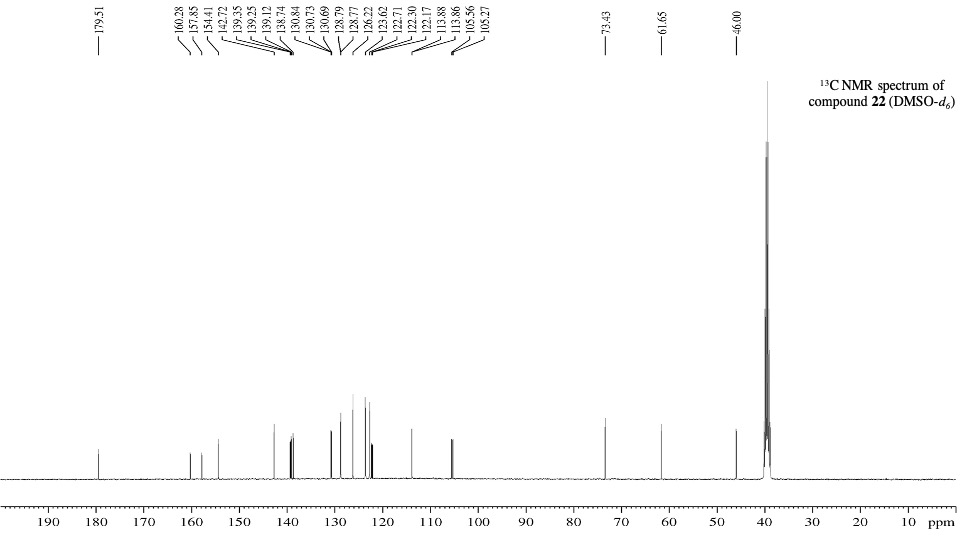


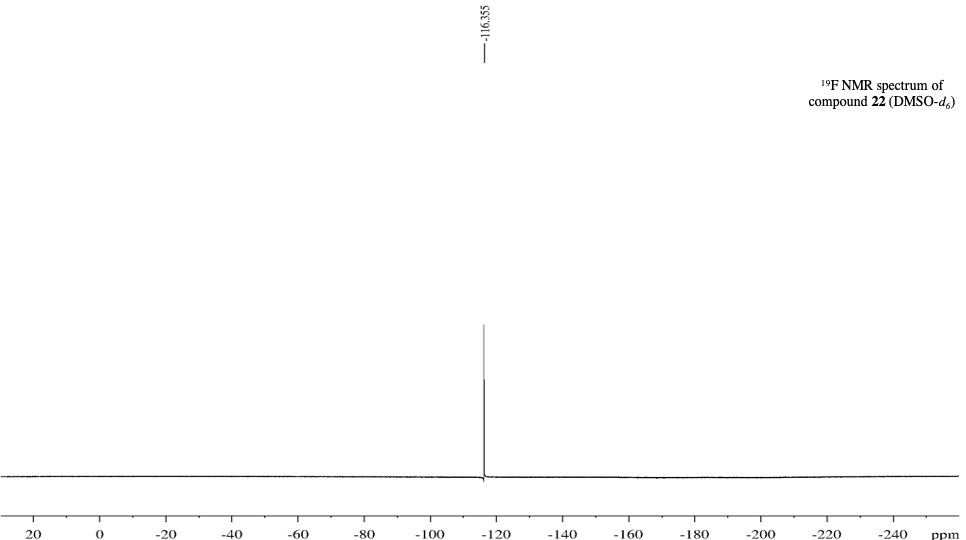


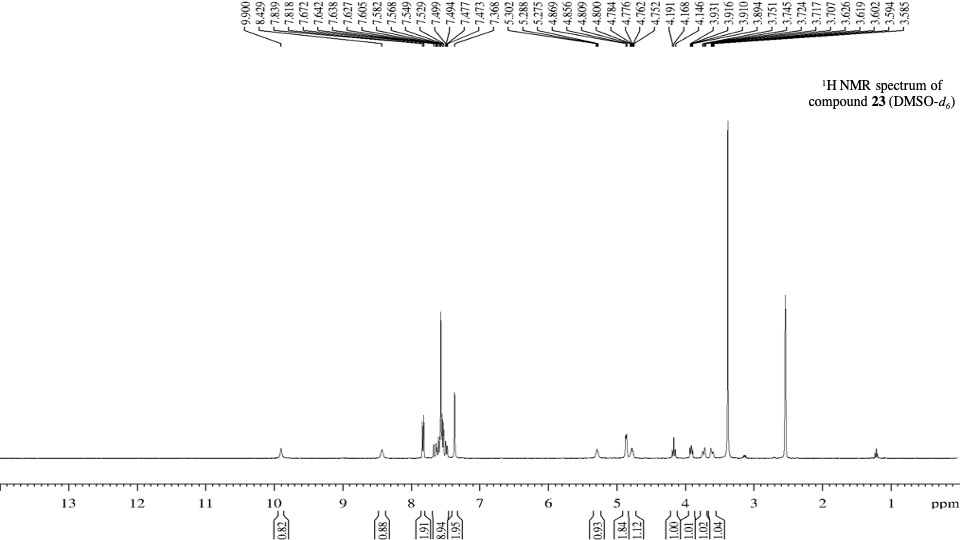


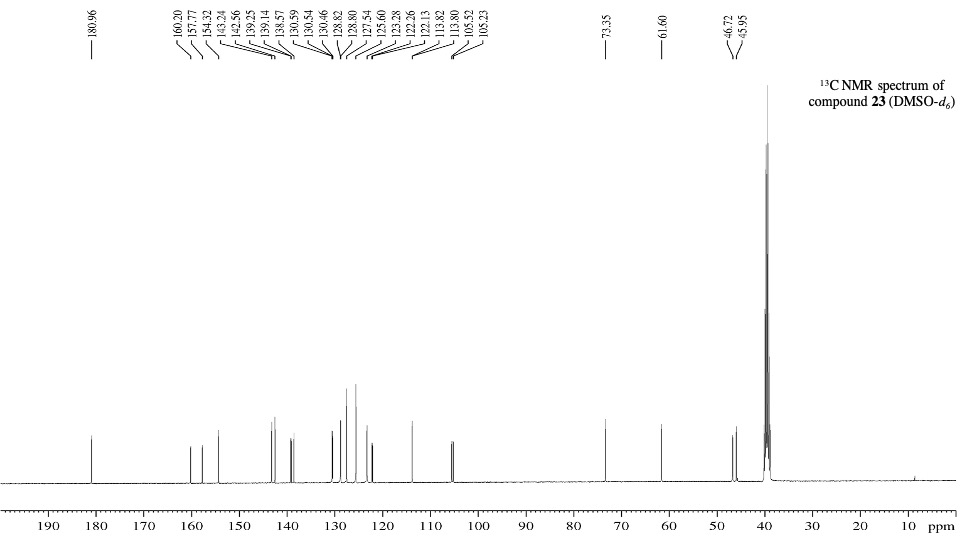


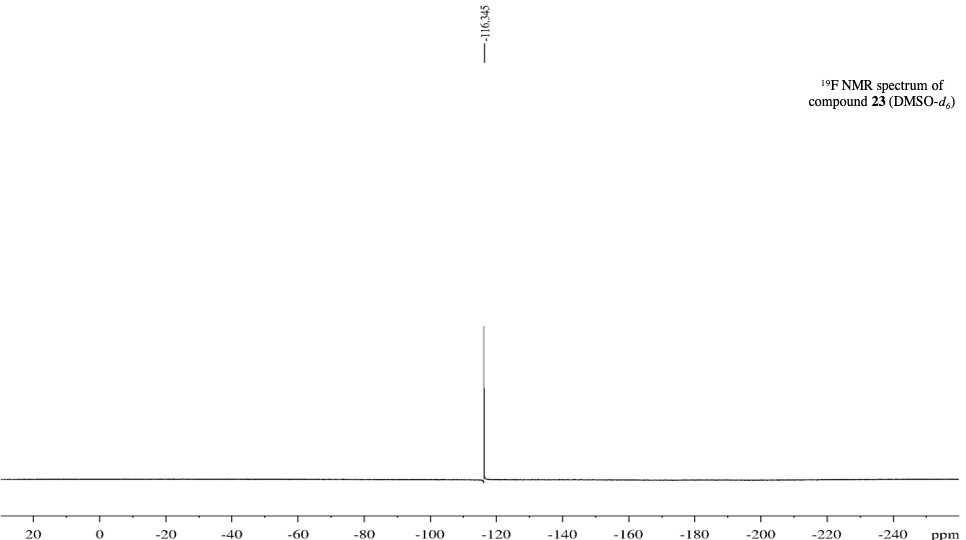


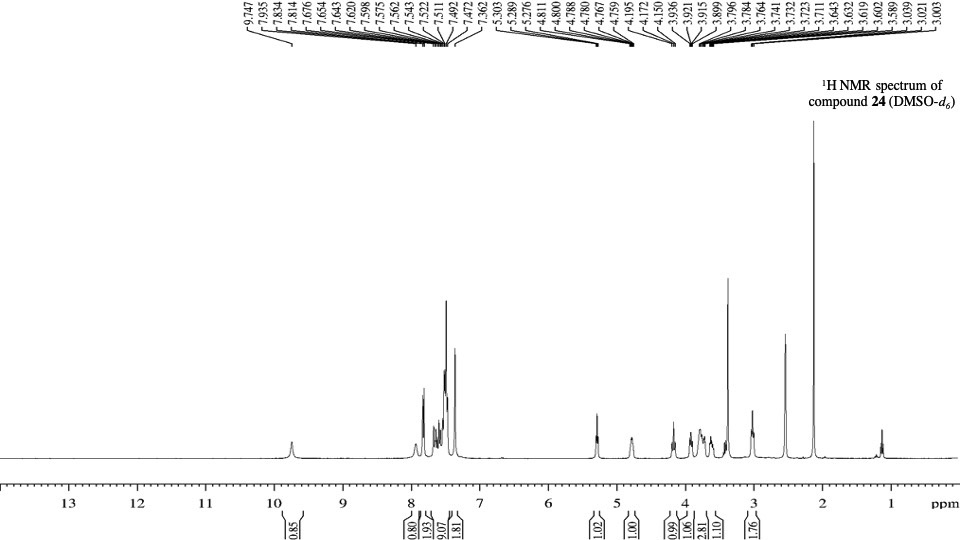


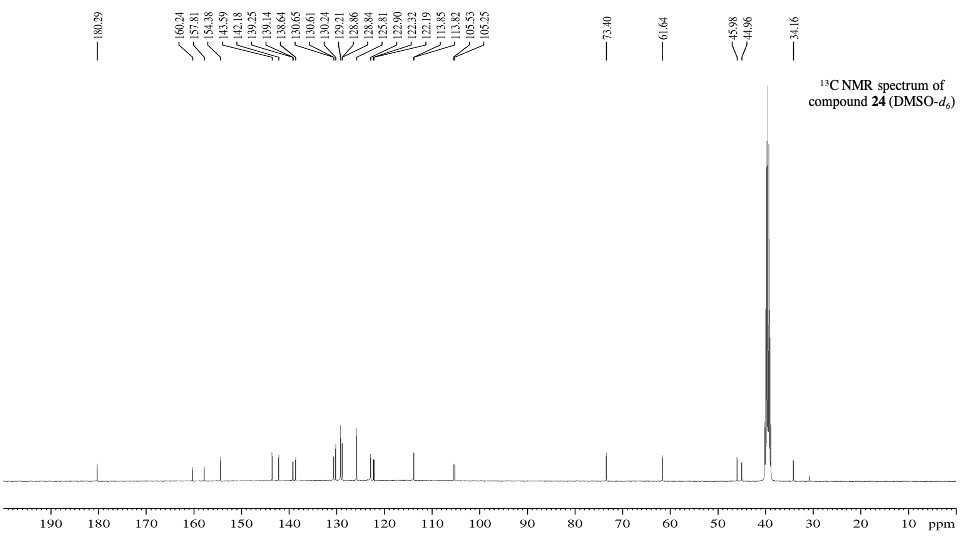


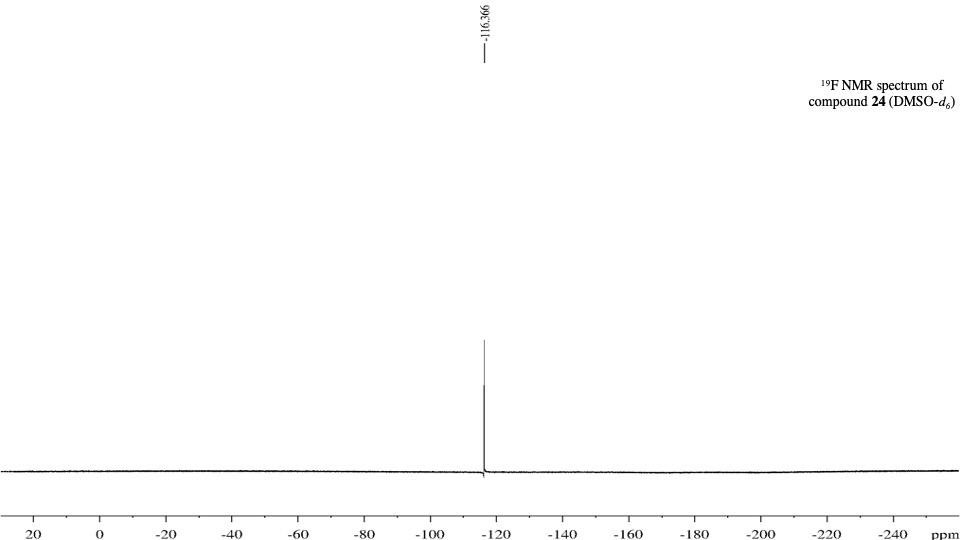


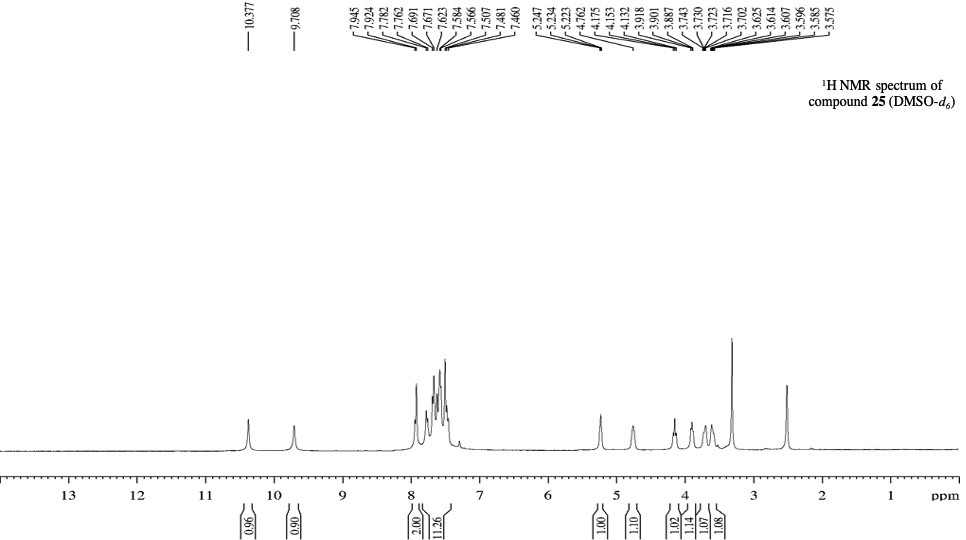


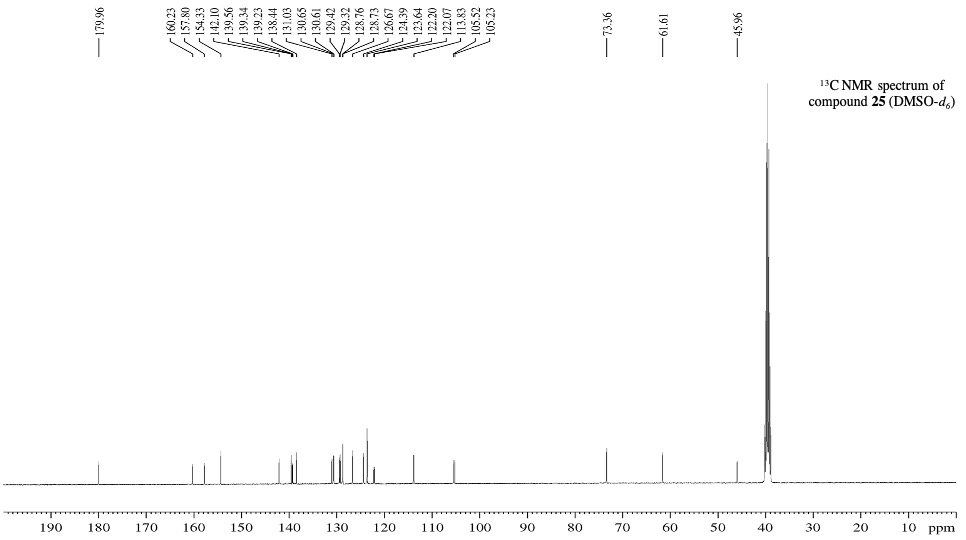


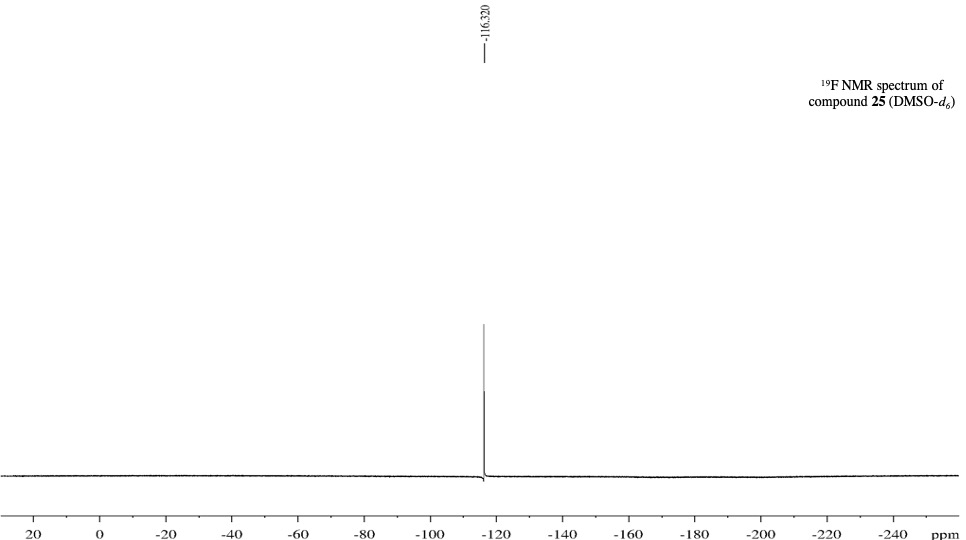


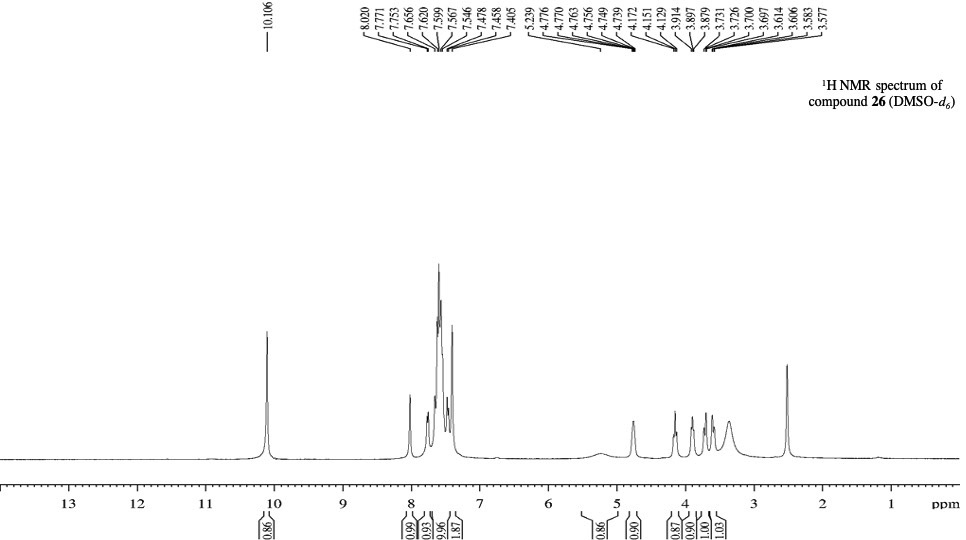


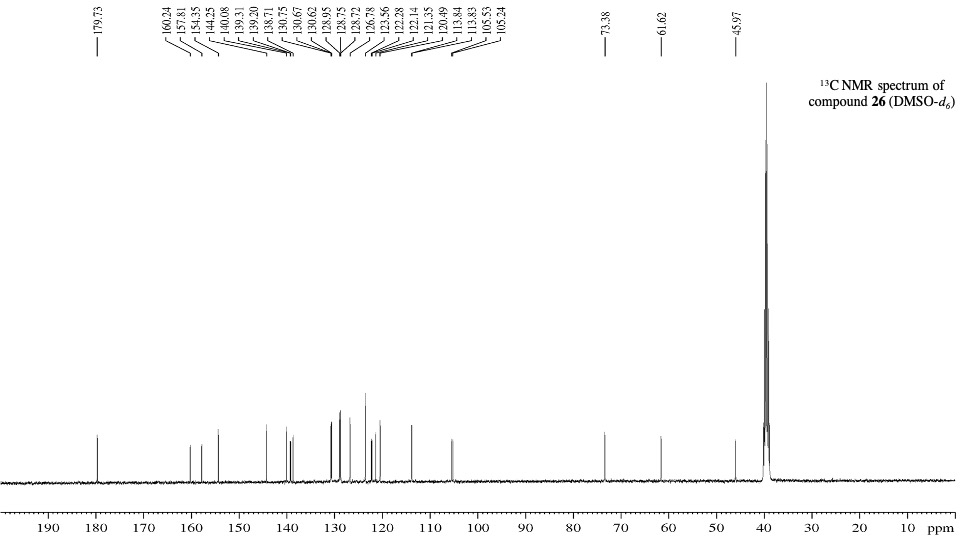


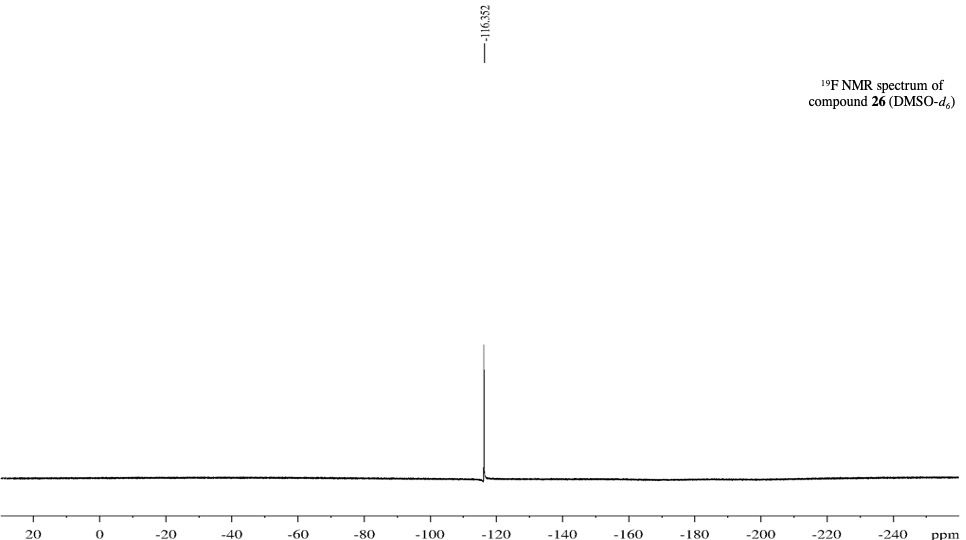

Supplement: SI [file NIHMS2082132-supplement-SI.docx]
